# A network of Notch-dependent and -independent her genes controls neural stem and progenitor cells in the zebrafish thalamic proliferation zone

**DOI:** 10.1242/dev.201301

**Published:** 2023-04-03

**Authors:** Christian Sigloch, Dominik Spitz, Wolfgang Driever

**Affiliations:** ^1^ Developmental Biology, Institute Biology 1, Faculty of Biology, University of Freiburg, Hauptstrasse 1, 79104 Freiburg, Germany; ^2^ Spemann Graduate School of Biology and Medicine SGBM, University of Freiburg, Albertstraße 19A, 79104 Freiburg, Germany; ^3^ Signalling Research Centres CIBSS and BIOSS, University of Freiburg, Schänzlestraße 18, 79104 Freiburg, Germany

**Keywords:** Neurogenesis, Neural stem cells, HES/Her transcription factors, Delta-Notch signaling, Neural proliferation zone, Thalamus, Zebrafish

## Abstract

Neural proliferation zones mediate brain growth and employ Delta/Notch signaling and HES/Her transcription factors to balance neural stem cell (NSC) maintenance with the generation of progenitors and neurons. We investigated Notch-dependency and function of her genes in the thalamic proliferation zone of zebrafish larvae. Nine Notch-dependent genes, *her2*, *her4.1-4.5*, *her12*, *her15.1-15.2*, and two Notch-independent genes, *her6* and *her9*, are differentially expressed and define distinct NSC and progenitor populations. *her6* prominently executes patterning information to maintain NSCs and the zona limitans intrathalamica Shh signaling activity. Surprisingly, simultaneous deletion of nine Notch-dependent her genes does not affect NSCs or progenitor formation, and *her4* overexpression only caused reduction of *ascl1b* progenitors. Combined genetic manipulations of Notch-dependent and -independent her genes suggest that *her6* in the thalamic proliferation zone prominently maintains NSCs and inhibits NSC-to-progenitor lineage transitions. The her gene network is characterized by redundant gene functions, with Notch-independent her genes better substituting for loss of Notch-dependent her genes than vice versa. Together, her gene regulatory feedback loops and cross-regulation contribute to the observed robustness of NSC maintenance.

## Teaser

**Summary:** Genetic analysis of a network of 11 Notch-dependent and -independent her genes reveals redundant activities to provide robust maintenance of neural stem cells in the zebrafish thalamic proliferation zone.

## INTRODUCTION

The zebrafish brain continually grows and adds new neurons throughout development and into adult stages, and has a high capacity to regenerate neurons after injury (Kishimoto et al., 2012; Zupanc, 2001). The source of these new neurons are neural stem cells (NSCs) in at least 16 adult neural proliferation zones (NPZs) mostly located at the ventricular wall (Adolf et al., 2006; Grandel et al., 2006; Zupanc et al., 2005). During development, the embryonic neuroepithelium establishes highly active late embryonic and early larval proliferation zones, which develop into neural stem cell niche-like adult proliferation zones. So far, the mechanisms that balance NSC maintenance and neurogenesis in these larval NPZs are not well understood.

Hairy and Enhancer-of-split related (HES/Her) factors in NPZs control neurogenesis by inhibiting proneural gene mediated differentiation (Ishibashi et al., 1994; Kageyama et al., 2007; Sasai et al., 1992). In mice, absence of the basic helix loop helix (bHLH) transcription factors HES1 and HES5 accelerates differentiation, accompanied by loss of radial glia and later-born cell types (Hatakeyama et al., 2004). In *Drosophila,* the expression of E(spl) genes is Notch-dependent (Jennings et al., 1994), while the expression of *hairy*, encoding a closely related bHLH factor, is directed by local patterning signals (Riddihough and Ish-Horowicz, 1991). In mouse, *Hes1* and *Hes5* are downstream targets of Notch signaling, although *Hes1* expression and stability are the result of additional Notch-independent input (Kageyama et al., 2007; Ohtsuka et al., 1999).

In zebrafish, the her gene family expressed in NPZs is significantly expanded compared with mammals (Zhou et al., 2012). Nine *Hes5* zebrafish homologs are located in two gene clusters, *her4.1*-*4.5* and *her12* on chromosome 23, and *her15.1*, *her15.2* and *her2* on chromosome 11. There are two *Hes1* homologs, *her6* and *her9*, and four *Hes6* homologs, *hes6*, *her8a*, *her8.2* and *her13*. *her4* and *her15*, *her6* and *her9*, as well as *her8a*, expression has been characterized in adult NSC zones (Chapouton et al., 2011; Zhou et al., 2012). Zebrafish homologs of *Hes1*, *Hes5* and *Hes6* have also been investigated in the neural plate during early embryonic development (Bae et al., 2005; Pasini et al., 2004; Shankaran et al., 2007; Takke et al., 1999; Webb et al., 2011). So far, however, little is known about potential cross-regulatory interactions or redundancies among her genes in NSCs and during neurogenesis in larval NPZs.

Here, we analyze the impact of Notch signaling and her family genes on expression of specific her genes in NSCs and neural progenitor cells (NPCs) to reveal the organization of her gene regulatory networks in neurogenesis. We focus on the highly active early larval thalamic proliferation zone (TPZ) at the ventricular wall of the diencephalon in the thalamus proper (dorsal thalamus), the zona limitans intrathalamica (ZLI) and the prethalamus (Mueller, 2012; Scholpp and Lumsden, 2010), together constituting the thalamic complex. Based on her gene expression, we define distinct NSC and NPC populations in the TPZ. Results from her gene loss-of-function and overexpression experiments reveal cross-regulatory interactions of Notch-dependent and -independent her genes in the TPZ. Our data indicate that Notch-independent her genes are important for NSC maintenance and patterning in the TPZ. Notch-dependent her genes, however, appear to be not strictly required for NSC maintenance, but might rather modulate NPCs.

## RESULTS

### Her family gene expression in the TPZs

**Fig. 1.**
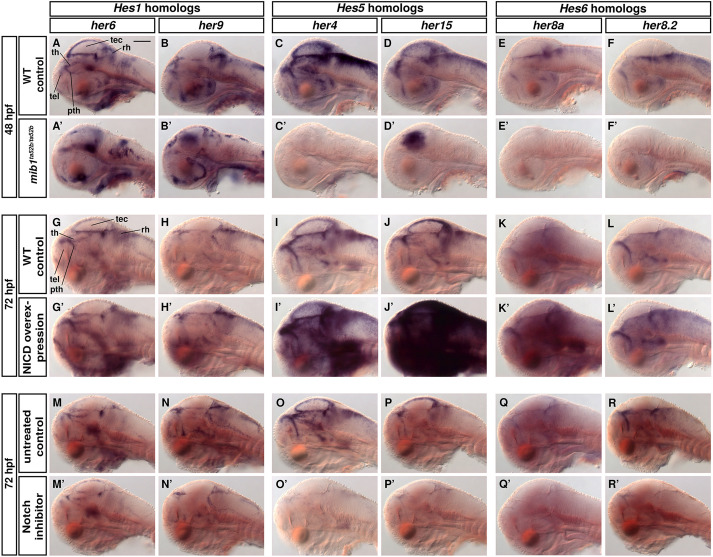
**Notch-dependent and -independent her gene expression. (A-R′)** *her6*, *her9*, *her4* (*her4.1-4.5* cluster probe), *her15* (*her15.1* and *her15.2* cluster probe), *her8a* and *her8.2* expression (WISH). 48 hpf WT sibling controls (*mib1^+/+^* or *mib1^+/ta52b^*) (A-F) and *mib1^ta52b^* mutants (A′-F′). (G-L′) Heat shock overexpression of NICD compared with heat-shocked WT siblings at 72 hpf. (M-R′) Notch inhibition by LY-411575 (64 to 72 hpf) in 72 hpf larvae and DMSO controls. Sagittal optical sections at midline (lateral views, single DIC image planes, anterior at left, dorsal at top). pth, prethalamus; rh, rhombencephalon; tec, tectum opticum; tel, telencephalon; th, thalamus. Scale bar: 100 µm.

Based on published information (www.zfin.org; Chapouton et al., 2011; Shankaran et al., 2007; Sieger et al., 2004; Zhou et al., 2012) on zebrafish her family genes (Table S1), we selected those with expression in the diencephalon, namely homologs of mammalian *Hes1* (*her6*, *her9*), *Hes5* (*her4;12* cluster and *her2;15* cluster) and *Hes6* (*her8a*, *her8.2*), for analysis of their expression patterns in the TPZ by whole-mount *in situ* hybridization (WISH; Fig. 1A-L; Fig. S1A-H). Genes within each cluster have high sequence similarity. Our *her4* probe was designed to detect expression of all five *her4* genes (*her4.1-4.5*) combined (subsequently referred to as *her4*). Similarly, our *her15* probe detects *her15.1* and *her15.2* (referred to as *her15*). We analyzed 48 h post fertilization (hpf) to 96 hpf embryos and early larvae, when the TPZ is a highly active proliferation zone with a diversity of neurogenesis mechanisms involving *neurog1*, *ascl1* and *olig2* (Scholpp et al., 2009; Virolainen et al., 2012; see also Fig. 7C and Fig. 11A,B).

In the TPZ, *her6*, *her9*, *her4*, *her2* and *her15* expression were detected with strong WISH signals, mostly restricted to the wall of the diencephalic ventricle and the adjacent lateral ventricle (Fig. S1A′-H′). *her2* has a similar expression domain as *her15.1-15.2* in the same cluster. *her12* expression appeared to be restricted to the hindbrain and later the midbrain-hindbrain boundary (Figs S1E,E′, S2A,G) and, thus, despite being in a cluster with *her4* genes, differs significantly in expression. The *Hes6* homologs *her8a* and *her8.2* are broadly expressed in NPZs at 48 hpf, but expression decreases, so that at 96 hpf we detected only low levels of *her8.2* and faint *her8a* expression in the TPZ (Fig. 1E,F,K,L; Fig. S1C-D′). In summary, while expression of the *Hes6* homologs *her8a* and *her8.2* decreases during early TPZ formation, *Hes1* (*her6*, *her9*) and *Hes5* (*her2*, *her4.1-4.5*, *her15.1-15.2)* homologs likely provide the major her gene activity in the maturing TPZ.

### Notch dependency of neural her gene expression

To assess Notch dependency of expression of *Hes1*, *Hes5* and *Hes6* homologs, we first analyzed *mind bomb 1* mutants (*mib1^ta52b^*) that lack Notch signaling (Itoh et al., 2003). Second, we globally overexpressed the Notch Intracellular Domain (NICD) by heat shock in 72 hpf larvae (Scheer and Campos-Ortega, 1999). Third, we treated larvae with the Notch inhibitor LY-411575 (Rothenaigner et al., 2011).

The *Hes1* homologs *her6* and *her9* are still expressed at largely normal levels in *mib1^ta52b^* mutant embryos (Fig. 1A-B′; with some ectopic *her9* signal in the midbrain), and NICD overexpression only slightly increased *her9* and hardly at all *her6* expression (Fig. 1G-H′). Upon Notch inhibitor treatment, *her6* and *her9* expression continued, albeit at slightly reduced levels (Fig. 1M-N′). These experiments demonstrate *her6* and *her9* expression to be Notch-independent at analyzed larval stages.

In contrast, the expression of *Hes5* (*her2*, *her4*, *her12* and *her15*) and *Hes6* (*her8a* and *her8.2*) homologs is lost in *mib1^ta52b^* mutants (Fig. 1C′-F′; Fig.S2A-D′). The ectopic *her15* expression in the tectum of *mib1* mutants is different from the expression in wild type (WT; Fig. 1D,D′) and, similar to *her9*, may reflect dysregulation. Expression of *Hes5* homologs was strongly induced by NICD, whereas the *Hes6* homologs showed only a moderate increase in expression (Fig. 1I-L′). Upon Notch inhibitor treatment, expression of *Hes5* and *Hes6* homologs was nearly absent (Fig. 1O-R′; Fig.S2G-I′), except *her2*, which was expressed at reduced levels (Fig. S2J,J′). Thus, *her2* may be only partly controlled by Notch.

Expression of *her6* and *her9* are hereafter referred to as Notch-independent. In contrast, *her4* and *her15* expression are strictly Notch-dependent. Given that *her8a* and *her8.2* have only low and declining levels of expression in the TPZ on the fourth day of development, we did not include these two genes in our further analysis.

### Differential expression of her genes in the thalamus proper, prethalamus and ZLI

**Fig. 2.**
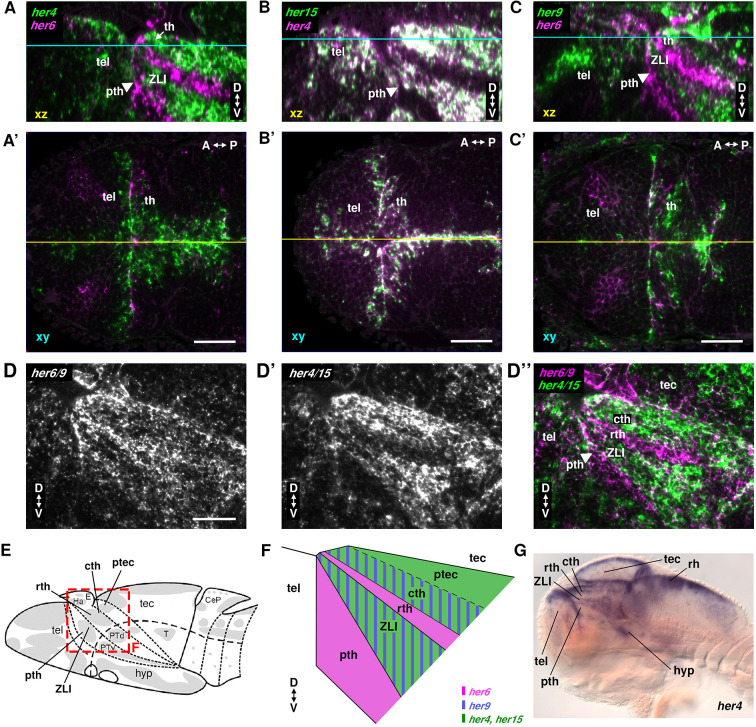
**Co-expression analysis of her genes in the thalamic complex.** (A-D″) Confocal imaging of double-fluorescent WISH at 72 hpf, probes and colors as indicated. (A-C) Lateral views of midline sagittal orthogonal reconstructions of dorsal view horizontal stacks (levels: yellow line in A′-C′). (A′-C′) Dorsal views, optical horizontal section level indicated in cyan in A-C. (D-D″) Lateral views of combined *her6/her9* (*her6* and *her9* probes mixed) and combined *her4/her15* (*her4.1-4.5* and *her15.1-15.2* probes mixed). (E) Schematic of a 3 dpf zebrafish larval brain (Mueller and Wullimann, 2016). Gray, proliferation zones; dashed line, alar-basal boundary. Red box indicates the thalamic complex, shown enlarged in F. (F) Schematic of her gene expression domains at the ventricular wall of the alar diencephalon. Striped areas indicate that genes are co-expressed or expressing cells intermingled. (G) *her4* WISH 72 hpf larva, sagittal optical section. A-P, anterior-posterior; CeP, cerebellar plate; cth, caudal thalamus; D-V, dorsal-ventral; E, epiphysis; Ha, habenula; hyp, hypothalamus; PTd, dorsal part of posterior tuberculum; ptec, pretectum; pth, prethalamus; PTv, ventral part of posterior tuberculum; rh, rhombencephalon; rth, rostral thalamus; T, midbrain tegmentum; tec, tectum opticum; tel, telencephalon; th, thalamus; ZLI, zona limitans intrathalamica. Scale bars: 50 µm.

We next investigated, using fluorescent WISH, whether Notch-dependent and Notch-independent her genes are differentially expressed in the TPZ (Fig. 2). High *her4* and *her6* expression was restricted to the periventricular TPZ wall and co-expressed with the NSC marker *sox2* (Fig. S3A-B″). At 24 hpf, *her6* is broadly expressed in the presumptive thalamic complex (Scholpp et al., 2009). Then, *her6* expression is progressively restricted, and at 72 hpf is largely absent from the ZLI but maintained in the rostral thalamus and prethalamus (Fig. 2A,A′,F; Scholpp and Lumsden, 2010). Absence of high levels of *her6* co-expression with *irx1b* (caudal thalamus) or with *shha* (ZLI) confirms restriction of the major *her6* expression to rostral thalamus and prethalamus (Fig. S3C,D). However, there is some minor co-detection of *shha* and *her6* at the border of the ZLI. At the ventricle along the midline, *her4* is strongly expressed where *her6* is absent, namely in the ZLI, caudal thalamus and the pretectum (Fig. 2A). In the caudal thalamus, *her4* is also expressed laterally adjacent to the ventricular wall (Fig. 2A′). *her4* and *her15* are mostly co-expressed in the ZLI, caudal thalamus and pretectum (Fig. 2B,B′,G). *her9* is expressed only weakly in the ZLI and the caudal thalamus. *her6* and *her9* are expressed in rather non-overlapping territories (Fig. 2C,C′).

We also directly compared the expression of Notch-dependent *her4* and *her15* combined with Notch-independent *her6* and *her9* combined, using pairwise mixed probes, and found that their expression domains together cover the whole ventricular zone of the thalamic complex (Fig. 2D-D″). *her4*, *her15* and, to a lesser extent, *her9* are co-expressed in the ZLI and the caudal thalamus, whereas *her6* is selectively expressed in prethalamus and rostral thalamus (Fig. 2E,F). *her4*, *her6* and *her9* triple fluorescent hybridization chain reaction (HCR) analyzed at cellular resolution (Fig. S4) revealed that domains of high expression of each gene at the ventricle are mostly exclusive at 72 hpf.

Distant from highly *sox2*-expressing ventricular NSCs, we detected more basally *her4* expression at a lower level in cells which may correlate to NPCs. To validate this hypothesis, we analyzed *sox2*, *ascl1b* and *neurog1* expression (Fig. S5; Movie 1) and found high *sox2*-expressing cells (Sox2^high^) at the ventricular walls largely devoid of *ascl1b* and *neurog1* expression, corresponding to NSCs. In addition, we found a low *sox2*-expressing domain (Sox2^low^) in the region corresponding to low *her4* expression, which also expresses *ascl1b* or *neurog1*, and thus are NPCs. Based on anti-phospho-Histone H3 (pH3) anti-Sox2 double immunofluorescence, we determined the pH3^+^ versus Sox2^+^ count ratios in WT. The Sox2^high^ NSCs have significantly higher pH3^+^ versus Sox2^+^ count ratios, and thus mitotic activity, compared with Sox2^low^ NPCs (*P*=0.000249; Fig. 7E and Table S7).

In the Sox2^low^ NPC region (Fig. S6), *neurog1* expression largely overlaps with *her4*, but not with *her6* and *her9.* Similarly, expression of *ascl1a/b* is largely exclusive from the *her6*-expressing regions (Fig. S6D). *ascl1a/b* expression overlaps widely with *dla* in the Sox2^low^ *her4* region (Fig. S6E), suggesting that Delta and Notch-dependent her genes control *neurog1* and *ascl1a/b* in these NPC regions. To investigate the relationship of Delta signaling and her genes in NSC and NPC regions, we analyzed expression of *dla* combined with *sox2*, *her4*, *her6* and *her9* (Fig. S7). Surprisingly, *dla* expression appears to be higher in the NPC Sox2^low^ region than in the Sox2^high^ ventricular zone (Fig. S7A). *her4* and *dla* expression largely overlap in the NPC region (Fig. S7B). In contrast, regions with highest levels of *her6* and *her9* expression are devoid of *dla* (Fig. S7C-D‴).

Our data reveal at least three fundamentally distinct her-expressing cell populations in the TPZ (Fig. 11A,B): (1) Sox2^high^ NSC type 1 at the ventricular wall with predominant *her6* but absent *dla* expression; (2) Sox2^high^ NSC type 2 at the ventricular wall with high *her4* and/or *her15*, low *her9* and, in most regions, also *dla* expression, but absent *her6* expression; (3) Sox2^low^ NPCs away from the ventricular wall with *dla*, either *ascl1b* or *neurog1*, and low-level expression of *her4*.

### Auto- and cross-regulation of Notch-independent her genes

**Fig. 3.**
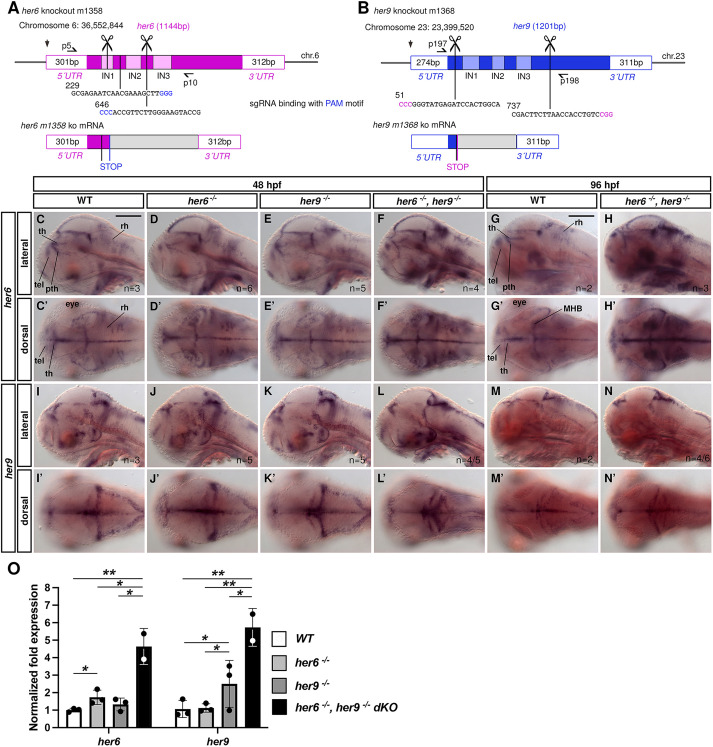
***her6* and *her9* single and double mutants reveal auto- and cross-regulation.** (A,B) CRISPR/Cas9 deletion of the bHLH domains in *her6* (A) and *her9* (B). Straight vertical lines, sgRNA binding sites; scissors, endpoints of the small deletion; half-arrows, genotyping primers. (C-N′) WISH for *her6* or *her9*, genotypes as indicated. (C-N) Lateral single sagittal optical planes. (C′-N′) Dorsal views. *n*/*n* indicate numbers of embryos with phenotype shown in panel/total analyzed. (O) qPCR analysis of *her6* or *her9* expression in *her6* and *her9* single and double 96 hpf mutants. Data are mean±s.d. *P<0.05, **P<0.01; two-tailed, two-sample equal variance *t*-test (Table S6). MHB, midbrain-hindbrain boundary; pth, prethalamus; rh, rhombencephalon; tel, telencephalon; th, thalamus. Scale bars: 200 µm.

We tested genetically whether *her6* and *her9* might cross-repress each other. We used CRISPR/Cas9 to delete large parts of the bHLH domains of *her6* and *her9*, and isolated mutant alleles with premature stop codons that leave the remaining aminoterminal peptides non-functional (Fig. 3A,B). Although *her6* and *her9* single mutant embryos have largely normal morphology, double mutants display an abnormal TPZ phenotype as well as malformations in eye development (Fig. S8A,A′), as reported for *Hes1* mutant mice (Nakamura et al., 2008, 2000; Tomita et al., 1996).

We analyzed *her6* and *her9* single and double mutants (Fig. 3C-N) using WISH probes that detect the shortened mutant transcripts. At 48 hpf, *her6* expression levels in several regions appear to be enhanced in *her6* mutants, and even stronger in *her6,her9* double mutants, whereas *her6* expression in *her9* mutants appears to be normal in most brain regions (Fig. 3C-F′). Upregulation of *her6* in double mutants is even stronger at 96 hpf (Fig. 3G-H′). *her9* expression levels in *her6* and *her9* single and double mutants at 48 hpf appear to not be strongly affected, whereas *her9* expression at 96 hpf is slightly increased in double mutants (Fig. 3I-N′). We analyzed *her6* and *her9* mRNA levels by qPCR on genotyped 96 hpf embryonic heads. We found a significant upregulation of *her6* transcripts in *her6* mutants and of *her9* transcripts in *her9* mutants (Fig. 3O; *P*-values see Table S6), indicating negative autoregulation. Upregulation of *her6* and *her9* transcripts in *her6*,*her9* double mutants was significantly stronger than in single mutants (Fig. 3O). We conclude that *her6* and *her9* exert negative cross-regulation on each other, partially redundant with negative autoregulation.

### Combined loss of *her6* and *her9* activity affects NSCs, NPCs and ZLI

**Fig. 4.**
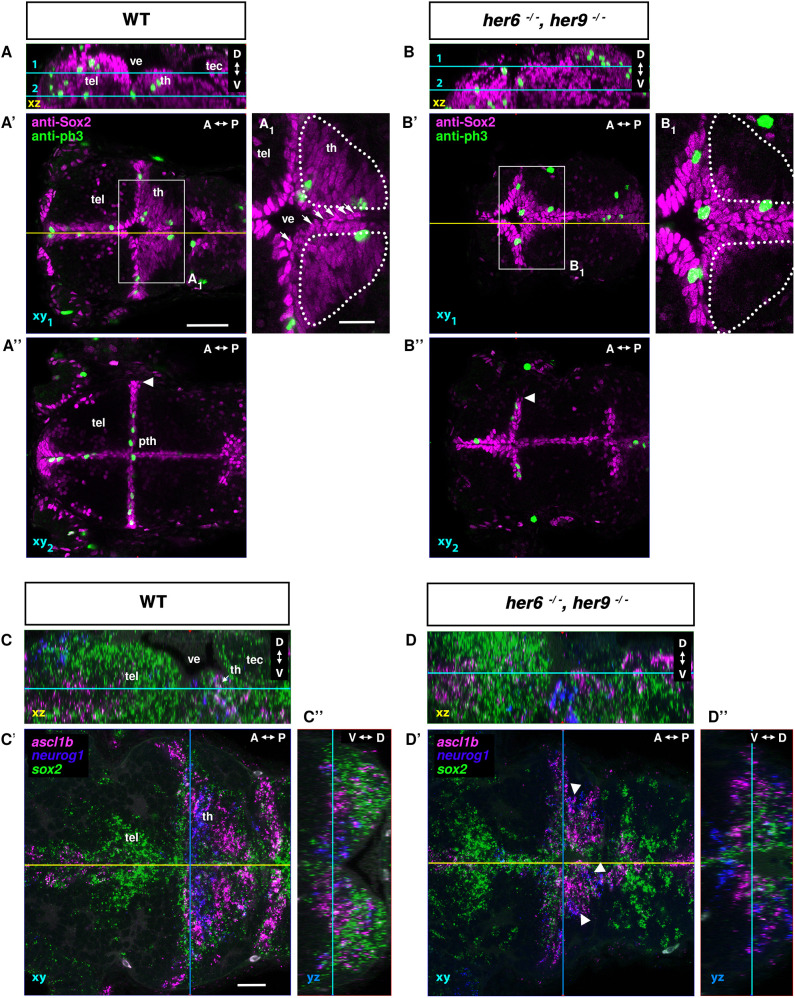
**NSC and NPC phenotypes in *her6*, *her9* double mutants.** (A-B″) Confocal image stacks of anti-Sox2 and anti-pH3 immunofluorescence of 72 hpf WT control (A) and *her6*, *her9* double mutants (B) (see Movie 2). (C-D″) HCR-RNA FISH for detection of *sox2*, *ascl1b* and *neurog1* expression. (A-D) Lateral view midline sagittal *xz* plane from orthogonal reconstructions. Cyan lines show dorsal view horizontal confocal planes 1 and 2 shown in A′-D′, A″,B″ (A′,B′ dorsal part of the diencephalon with the thalamus proper; A″,B″ more ventrally including the prethalamus). Yellow lines in A′-D′ show midline sagittal planes in A-D. A_1_ and B_1_ show magnifications of the boxed areas in A′ and B′. (C′,D′) Blue lines show level of frontal orthogonal reconstructions in C″ and D″ (at level of the lateral expansion of the ventricle). Arrowheads in A″ and B″ indicate the lateral expansion of ventricular Sox2^high^ cells. Arrowheads in D′ indicate the compacted expression domain of *neurog1* and *ascl1b* in NPCs of the mutant. A-P, anterior-posterior; D-V, dorsal-ventral; pth, prethalamus; tec, tectum opticum; tel, telencephalon; th, thalamus; ve, ventricle. Scale bars: 50 µm; 20 µm (A_1_,B_1_,C′).

In WT larvae, Sox2^+^ NSC nuclei line the thalamic ventricular walls, including the lateral ventricle away from the midline (Fig. 4A-D″; Movie 2), while Sox2^low^ nuclei with lower immunoreactivity are detected in subapical positions adjacent to the cell layer that forms the ventricular wall (Fig. 4A′,A_1_). *her9* and *her6* single mutants each have a largely normal distribution of Sox2 positive nuclei (Fig. S8F-H). In *her6*,*her9* double mutants, Sox2^low^ are largely absent from the subapical region (Fig. 4B′,B_1_). In addition, the Sox2^high^ nuclei along the lateral expansion of the ventricle were strongly reduced and missing at most lateral positions, indicating a loss of ventricular NSCs (Fig. 4A″,B″, white arrowheads; Movie 2). The number of subapical Sox2^low^ and Sox2^high^ cell nuclei were both significantly reduced in *her6*,*her9* double mutants (counts of nuclei in Table S7, Fig. 7E′). We used an anti-pH3 antibody to detect cells in mitosis. Along the lateral ventricular wall of *her6,her9* double mutants, where Sox2 expression is also missing, pH3^+^ nuclei were largely absent, and *ccnd1* expression, which accumulates during G1-to-S phase transition, was reduced (Fig. S8B-E′). Compared with WT, *her6,her9* double mutants in the TPZ had a similar distribution of pH3^+^ mitotic nuclei in the Sox2^high^ ventricular zone, and the count ratios of pH3^+^ among Sox2^high^ and Sox2^low^ cells were not significantly different from WT (Fig. 4A′,B′; Movie 2; Fig. 7E′; Table S7). Thus, despite reduction in Sox2^+^ cell number, the mitotic activity of the remaining NSCs and NPCs appears to not be significantly affected.

The strongest co-expression of *sox2* and *neurog1* or *ascl1b* is in Sox2^low^-expressing NPCs that are not in direct contact with the ventricle (Fig. S5A-F; Movie 1). *ascl1b* is expressed in a narrow stripe in the rostral thalamus, while *neurog1* is expressed in the caudal thalamus (Fig. 4C-C″; *ascl1a* is also expressed in prethalamus and pretectum, see also Fig. 11A-C). In *her6*,*her9* double mutants, *neurog1* and *ascl1b* cells are intermingled, and the combined domain is smaller and narrower (Fig. 4D-D″, arrowheads), indicating that the NPC domain is reduced. We also investigated *neurog1* and *ascl1a* expression using WISH from 24 hpf to 96 hpf at daily intervals (Fig. S9) and found enhanced *neurog1* expression at 24 hpf in *her6,her9* double mutants, but in most regions expression was comparable at 48 hpf, and reduced at 72 and 96 hpf compared with WT controls. *ascl1a* expression is enhanced in double mutants at 24 and 48 hpf, but similar to WT controls at 72 and 96 hpf. Thus, during early neurogenesis, premature differentiation is observed in *her6,her9* double mutants, but later the reduced number of NSCs may lead to reduced neurogenesis compared with WT.

**Fig. 5.**
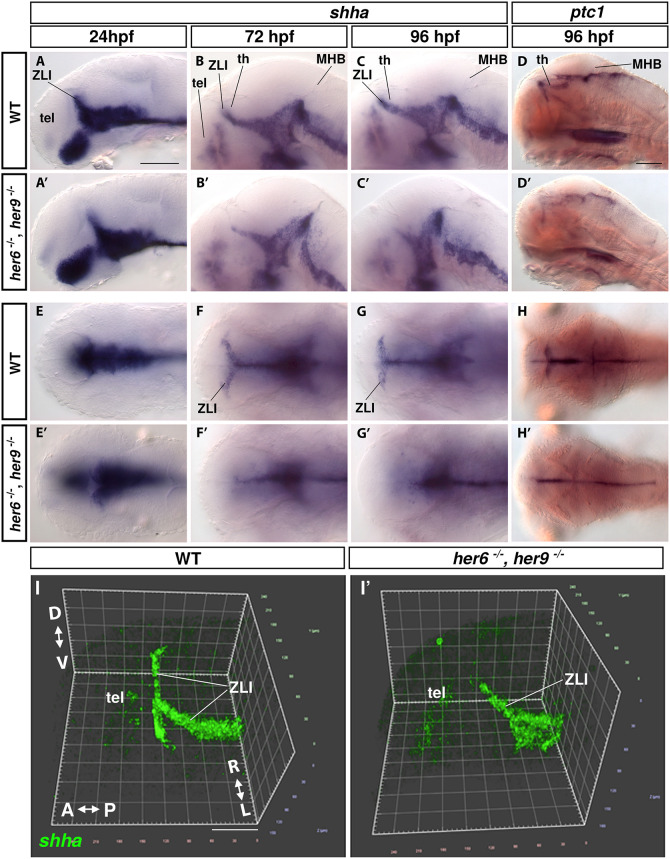
**Loss of ZLI *shha* expression in *her6*, *her9* double mutants.** (A-H′) WISH showing *shha* and *ptc1* expression in WT and *her6, her9* double mutants. For single mutants, see Fig. S10. (A-D′) Lateral views: single sagittal image planes at the midline. (E-H′) Dorsal views: single horizontal image planes at the level of the thalamus. (I,I′) 3D volume reconstruction of fluorescent WISH of *shha* in 72 hpf WT (I) and *her6*, *her9* double mutant (I′). A-P, anterior-posterior; D-V, dorsal-ventral; MHB, midbrain-hindbrain boundary; R-L, right-left; tel, telencephalon; th, thalamus; ZLI, zona limitans intrathalamica. Scale bars: 100 µm (A for A-C′,E-G′; D for D,D′,H,H′); 60 µm (I for I,I′).

In the WT thalamus, *shha* is expressed in the basal plate and the ZLI, where it extends along the walls of the lateral ventricle (Fig. 5A-D,I). Although at 24 hpf the *shha* expression in the ZLI appears to be largely normal in *her6,her9* double mutants (Fig. 5A,A′,E,E′), at 72 hpf and 96 hpf the ZLI *shha* expression domain is progressively lost both in its lateral and dorsal domains (Fig. 5). In *her6* single mutants *shha* expression along the ZLI lateral ventricular walls is variable and partially lost (Fig. S10, arrowheads). When we analyzed *shha* and *sox2* expression in *her6*,*her9* double mutants at 24 hpf, we found that *shha* expression was already reduced at stages when *sox2* was still broadly expressed in the neuroepithelium, and a distinct proliferation zone had not yet established (Fig. S11). At this stage, *her6* was still expressed in ZLI *shha* cells. We conclude that loss of Notch-independent *her* activity affects dorsal expansion of *shha* expression and establishment of *shha* in the ZLI temporally before loss of NSCs in the TPZ.

### Loss of the two Notch-dependent her gene clusters does not affect NSCs and NPCs

**Fig. 6.**
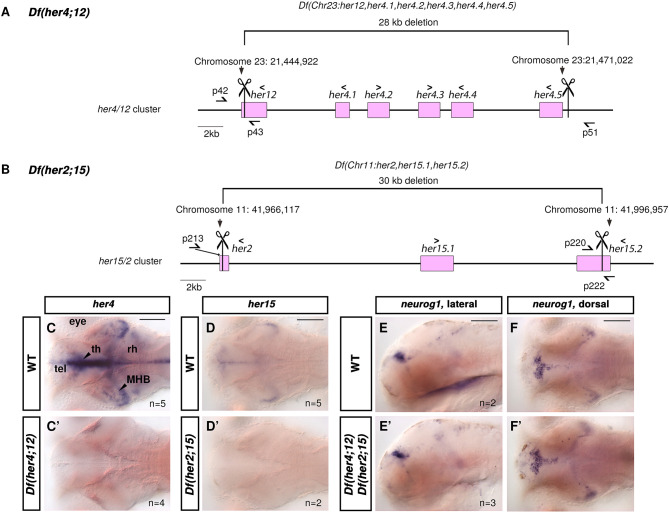
**Generation and characterization of *Df(her4;12)* and *Df(her2;15)* chromosomal deletions**. (A,B) Schematic of CRISPR/Cas9 strategy. Scissors, sgRNA positions; square brackets, deletion;>or<signs, 5′-to-3′ orientations of genes; half-arrows, binding sites genotyping primers. For details see Fig. S12. (C-F′) WISH for *her4*, *her15* and *neurog1* mRNAs in 96 hpf larvae, genotypes as indicated (C-D′,F,F′ dorsal view; E,E′ midsagittal optical section). n, embryos analyzed. MHB, midbrain-hindbrain boundary; rh, rhombencephalon; tel, telencephalon; th, thalamus. Scale bars: 100 µm.

The two *her4;12* and *her2;15* gene clusters active in the TPZ contain nine *Hes5* zebrafish homologs (Zhou et al., 2012) but no other genes (www.ensembl.org)*.* We generated large precise deficiencies of both chromosomal regions using CRISPR/Cas9 (Fig. 6A,B; Fig. S12): the 28 kb deficiency *Df(Chr23:her12,her4.1,her4.2,her4.3,her4.4,her4.5)m1364* or *m1365*, abbreviated as *Df(her4;12)*, and the 30 kb deficiency *Df(Chr11:her2,her15.1,her15.2)m1490*, abbreviated as *Df(her2;15)*. WISH for *her4* and *her15* demonstrated that there were no transcripts left in these mutants (Fig. 6C-D′).

**Fig. 7.**
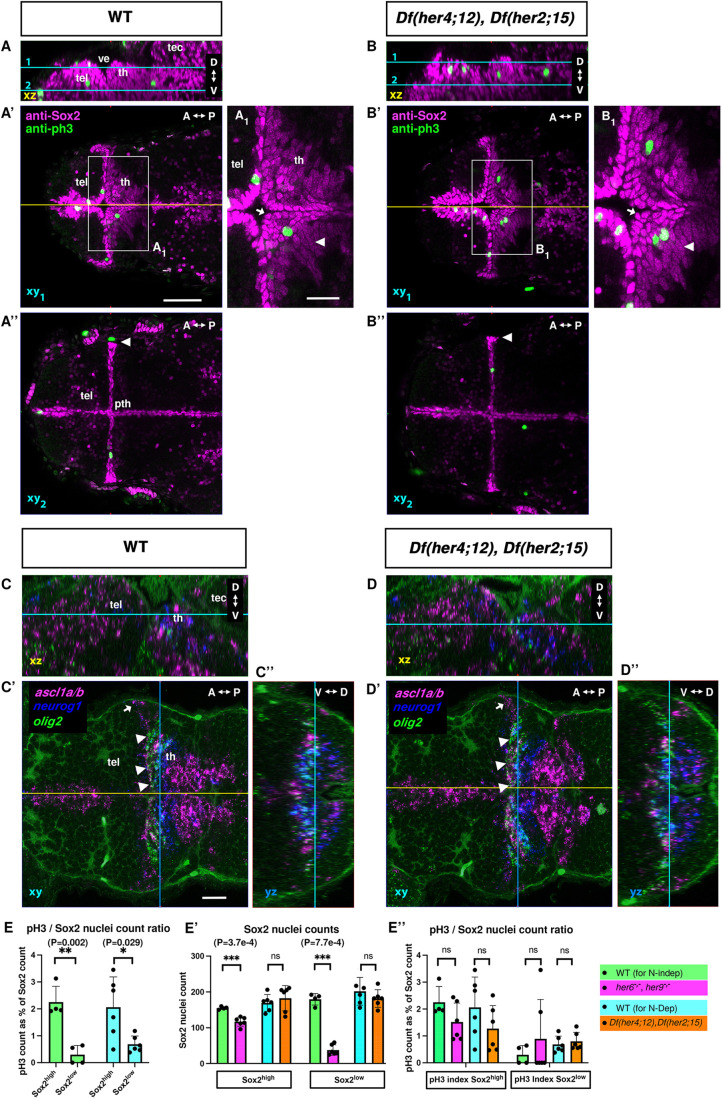
**NSC and NPC phenotypes in *Df(her4;12)*, *Df(her2;15*) double deficiency mutants.** (A-B″) Confocal image stacks of anti-Sox2 and anti-pH3 immunostaining in 72 hpf WT (A) and *Df(her4;12)*, *Df(her2;15)* double mutants (B). (C-D″) HCR-RNA FISH for detection of *ascl1a/b*, *neurog1* and *olig2* expression. (A-D) Lateral view midline sagittal *xz* plane from orthogonal reconstructions. Cyan lines show dorsal view horizontal confocal planes 1 and 2 shown in A′-D′, A″-B″ (A′-D′ dorsal part of the diencephalon with the thalamus proper; A″-B″ more ventrally including the prethalamus). Yellow lines in A′-D′ show midline sagittal planes in A-D. A_1_ and B_1_ show magnifications of the boxed areas in A′ and B′, with arrowheads indicating Sox2^low^-expressing cells. (C′,D′) Blue lines show level of frontal orthogonal reconstructions in C″ and D″ (at level of the lateral expansion of the ventricle). Arrowheads in A″ and B″ indicate the lateral expansion of ventricular Sox2^high^ cells. Arrowheads in C′ and D′ show *olig2* in parallel to the ventricular wall and adjacent to *neurog1*. Arrows show the Sox2^high^ NSC nuclei layer covering the ventricular wall. (E-E″) Anti-Sox2 and anti-pH3 nuclei counts for Notch-dependent (N-dep) and Notch-independent (N-indep) her mutants (Fig. 4; Table S8; Materials and Methods, ‘Microscopy and cell counts’). (E) pH3/Sox2^+^ count ratios in Sox2^high^ and Sox2^low^ cells. (E′) Sox2^high^ and Sox2^low^ nuclei counts in N-dep and N-indep her mutants. (E″) pH3/Sox2^+^ nuclei count ratios in Sox2^high^ or Sox2^low^ are not significantly changed in *her6,her9* double mutants or double deficiency mutants. Data are mean±s.d. Two sample, unequal variance two-tailed *t*-tests. Six biological replicates each except WT N-indep (four) replicates; single experiments. A-P, anterior-posterior; D-V, dorsal-ventral; ns, not significant; pth, prethalamus; tec, tectum opticum; tel, telencephalon; th, thalamus; ve, ventricle. A-P, anterior-posterior; D-V, dorsal-ventral; pth, prethalamus; tec, tectum opticum; tel, telencephalon; th, thalamus; ve, ventricle. Scale bars: 50 µm; 20 µm (A_1_,B_1_,C′).

We could not observe morphological abnormalities in *Df(her4;12)* or *Df(her2;15)* mutant larvae until 30 days post fertilization (dpf) or in fixed 96 hpf larvae (Fig. 6C′,D′). Therefore, we analyzed *Df(her4;12),Df(her2;15)* double mutants, which also appeared to be morphologically normal and have normal *neurog1* expression (Fig. 6E-F′). The Sox2^high^ NSC nuclei layer covering the ventricular wall of the thalamus proper (arrow in Fig. 7A_1_,B_1_) and the adjacent subapical Sox2^low^ NPC nuclei (arrowhead in Fig. 7A_1_,B_1_) appear to be normal. Both in WT and double mutants, the shape of the nuclei at the ventricular wall is spherical, and the subapical Sox2^low^ nuclei are elongated. Sox2^high^ nuclei line the lateral ventricle along its full lateral extent (Fig. 7A″,B″, arrowheads). The counts of Sox2^high^ and Sox2^low^ nuclei, the distribution of pH3^+^ nuclei (Fig. 7A-B″,E′; Movie 3), the count ratio of pH3^+^ to Sox2^high^ and Sox2^low^ cells (Table S7; Fig. 7E′,E″) and the expression of *ccnd1* (Fig. S8) appear to not be significantly different in *Df(her4;12)*,*Df(her2;15)* compared with WT, suggesting normal proliferation in the TPZ. Thus, NSCs and NPCs appear to develop normally in the TPZ devoid of Notch-dependent her genes.

We also analyzed her gene expression in *her6*,*her9* and in *Df(her4;12),Df(her2;15)* double mutants. *her6* and *her9* expression is largely unaffected in *Df(her4;12),Df(her2;15)* double mutants, in line with their Notch-independent regulation. *her4* and *her15* expression appears to be decreased in the NPC area of *her6* and *her9* double mutants (Fig. S13A′,B′). At the ventricular wall *her4* and *her15* expression do not invade into the *her6* domains in the rostral thalamus and prethalamus (Fig. S13A,B), suggesting that mechanisms other than repression by *her6* limit *her4* and *her15* expression in these domains.

We analyzed expression of *neurog1*, *ascl1a/b* and *olig2* in NPCs. *olig2* is expressed in a stripe of cells parallel to the ventricular wall, adjacent to *neurog1* (Fig. 7C-C″, arrowheads). Expression domains of all four genes appear to be normal in *Df(her4;12),Df(her2;15)* double mutants (Fig. 7D-D″). Although more subtle changes cannot be excluded, the NSC and the NPC compartments of the TPZ in the absence of Notch-dependent her gene activity appear to be very similar to WT.

### Her4 and Her6 overexpression reveal selective cross-regulation of her genes

**Fig. 8.**
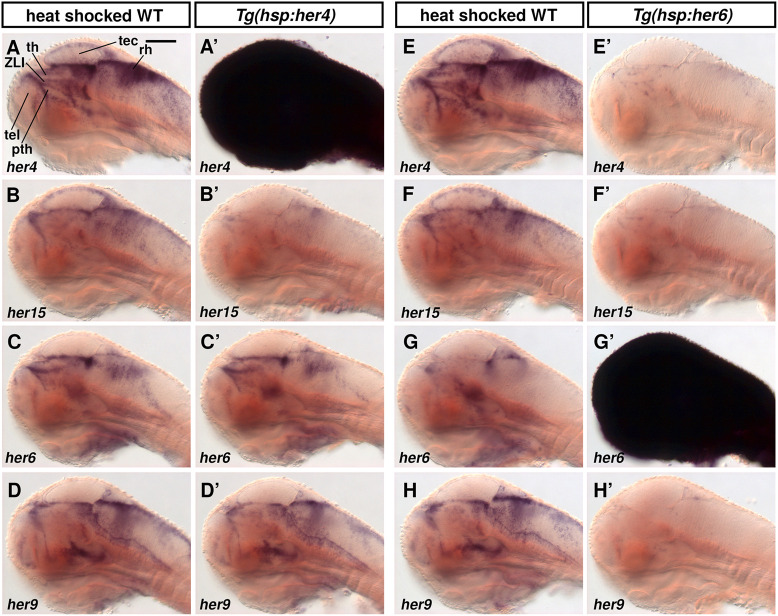
**Overexpression of Her4 and Her6 reveals differential cross-regulation of her genes.** (A-H′) WISH for her gene expression after heat shock-induced overexpression of Her4 or Her6. WT heat-shocked sibling controls (A-H), *Tg(hsp:her4-FLAG)* (A′-D′) and *Tg(hsp:her6-FLAG*) (E′-H′) transgenic embryos. Larvae were heat shocked at 70 hpf for 30 min and fixed at 72.5 hpf, except for G and G′ that were fixed at 71.5 hpf because *her6* transcript levels decrease faster after heat shock than *her4* transcript levels. *her4* probe detects *her4.1-4.5. her15* probe detects *her15.1* and *her15.2*. Sagittal optical sections close to midline, anterior at left, dorsal up. pth, prethalamus; rh, rhombencephalon; tec, tectum opticum; tel, telencephalon; th, thalamus; ZLI, zona limitans intrathalamica. Scale bar: 100 µm.

We next investigated potential cross-regulation between Notch-dependent and -independent her genes by heat shock-driven overexpression of Her4 or Her6 using *Tg(hsp:her4-FLAG)* and *Tg(hsp:her6-FLAG)* transgenic lines. When *Tg(hsp:her4-FLAG)* larvae were analyzed using WISH 2.5 h post heat shock, *her4* was confirmed to be overexpressed (Fig. 8A,A′), and we detected downregulation of *her15* expression (Fig. 8B,B′). Her4 overexpression, however, did not affect the expression levels of *her6* and *her9* (Fig. 8C-D′). In contrast, overexpression of Her6 strongly downregulated *her9* and both *her4* and *her15* (Fig. 8E-H′). This suggests that Notch-independent her genes negatively repress Notch-dependent her genes, while Notch-dependent her genes repress other Notch-dependent her genes, but do not regulate Notch-independent her genes.

### Overexpression of Her6, but not Her4, strongly downregulates proneural gene expression

**Fig. 9.**
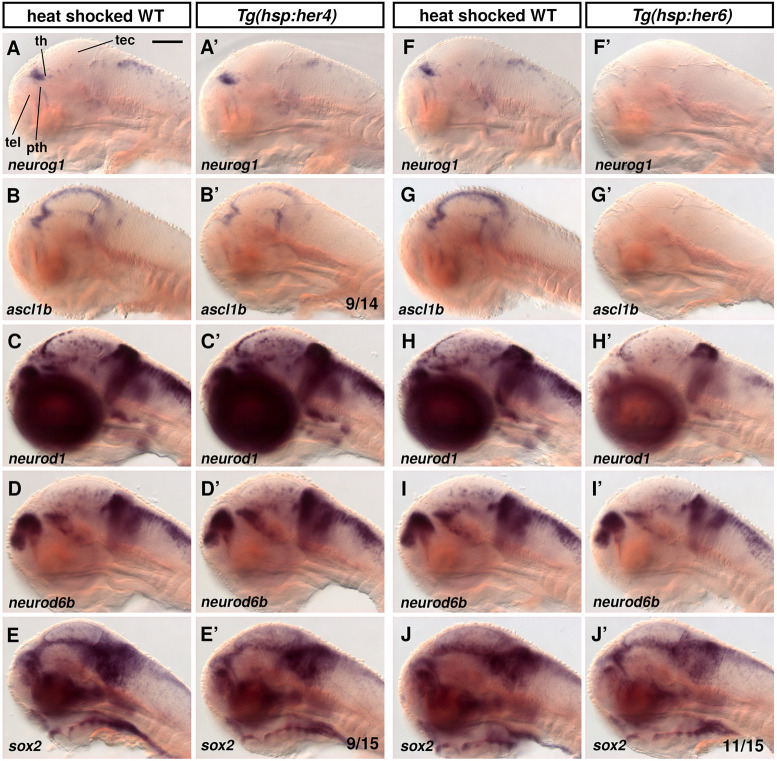
**Overexpression of Her4 and Her6 differentially affect proneural gene expression.** (A-J′) Expression of proneural genes and *sox2* after heat shock-induced overexpression of Her4 or Her6. *Tg(hsp:her4-FLAG)* (A′-E′), *Tg(hsp:her6-FLAG)* (F′-J′) and WT sibling controls (A-J) were heat shocked at 70 hpf for 30 min, embryos fixed at 72.5 hpf. See Table S5 for numbers of embryos analyzed. For B′, E′ and J′ the most representative pattern each are shown (number phenotype shown of total analyzed see bottom right). Sagittal optical sections close to midline, anterior at left, dorsal up. pth, prethalamus; tec, tectum opticum; tel, telencephalon; th, thalamus. Scale bar: 100 µm.

We tested whether Her4 or Her6 might differentially affect proneural gene expression. Upon Her4 heat shock-driven overexpression at 70 hpf and fixation 2.5 h after heat shock, *neurog1* expression appeared to be largely unchanged, whereas *ascl1b* was reduced in 9 out of 14 larvae (Fig. 9A-B′). Late progenitors, evaluated by *neurod1* and *neurod6b* expression, were unchanged 2 h after *her4* heat shock (Fig. 9C-D′). *sox2* expression was slightly reduced in 9 out of 15 *her4* heat-shocked larvae (Fig. 9E-E′). In contrast, overexpression of Her6 strongly downregulated *neurog1* and *ascl1b* expression, and to a lesser extent the late progenitor markers *neurod1* and *neurod6b* (Fig. 9F-I′). In addition, *sox2* expression appeared to be slightly reduced in 11 out of 15 Her6 heat-shock larvae (Fig. 9J-J′). We also tested whether overexpression of Her4 or Her6 might have a prolonged effect on proneural gene expression late after heat shock. We analyzed larvae 6 h after the beginning of the 30 min heat shock and found *neurog1* and *ascl1b* expression at normal levels (Fig. S14A-D′), despite their downregulation 2.5 h after heat shock. This suggests that the NSC/NPC regulatory network is robust against perturbations in Her6 expression level, and rapidly re-establishes normal activity. Together, our findings reveal a strong regulatory impact of Her6 on NPCs and NSCs, and indicate that Notch-independent Her6 might be a much more potent inhibitor of neurogenesis compared with Notch-dependent Her4.

### Combined loss of activity of 11 Notch-dependent and -independent her genes

**Fig. 10.**
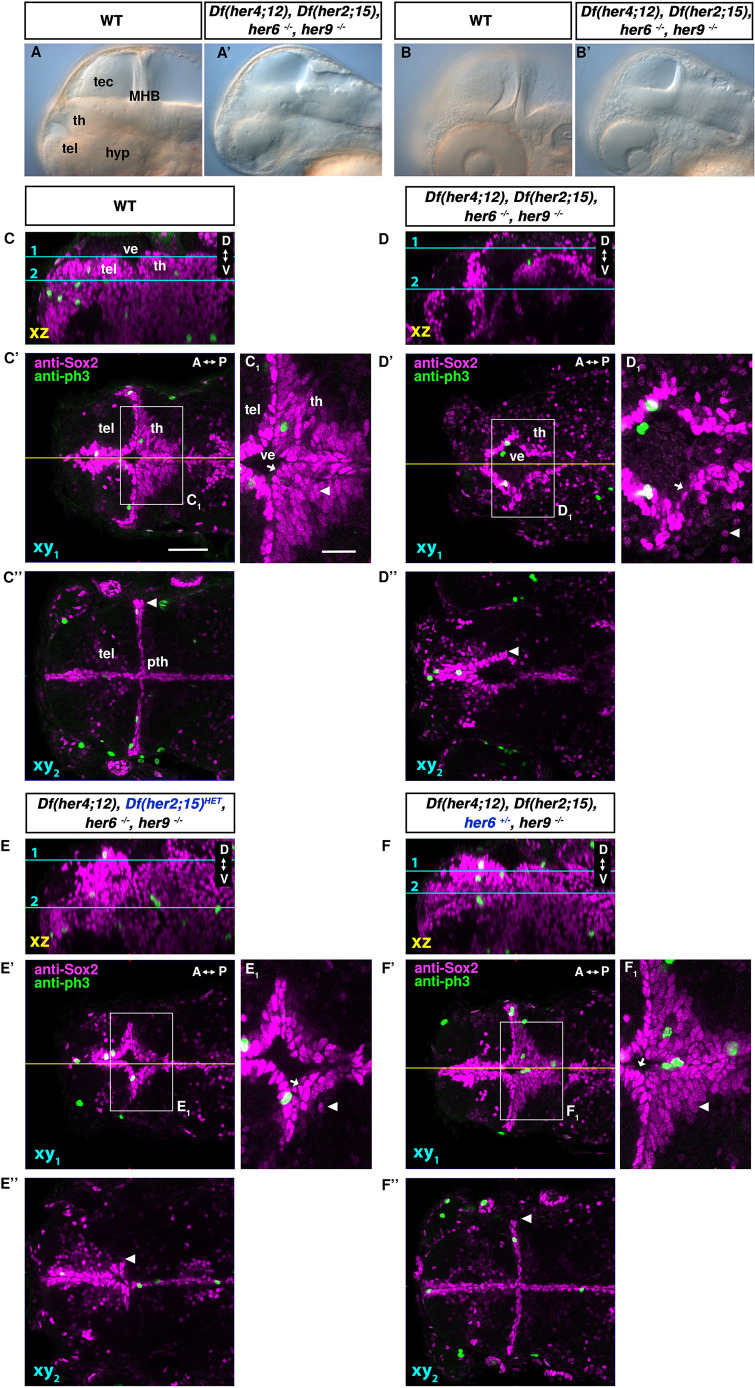
**Analysis of the her activity depleted *her*UDM mutant phenotype reveals prominent role of *her6*.** (A-B′) Live phenotype of WT and *Df(her4;12),Df(her2;15),her6,her9* mutant (*her*UDM) embryos, lateral views at 56 hpf; midsagittal optical section (A), parasagittal optical section at level of lens (B). (C-F″) Anti-Sox2 and anti-pH3 immunofluorescence in 72 hpf embryos, genotypes indicated. (C-F) Lateral view midline sagittal *xz* plane from orthogonal reconstructions. Cyan lines show dorsal view horizontal confocal planes 1 and 2 shown in C′-F′ and C″-F″, respectively (C′-F′ dorsal part of the diencephalon with the thalamus proper; C″,F″ more ventrally including the prethalamus). (C′-F′) Yellow lines show midline sagittal planes in C-F. C_1_-F_1_ show magnifications of boxed areas in C′-F′. Arrowheads in C_1_-F_1_ show Sox2^low^ cells. Arrowheads in C″-F″ indicate the lateral expansion of ventricular Sox2^high^ cells. Arrows show the Sox2^high^ nuclei layer covering the ventricular wall. For confocal stacks C/D and E/F see Movies 4 and 5. A-P, anterior-posterior; D-V, dorsal-ventral; tel, telencephalon; th, thalamus; ve, ventricle. Scale bars: 50 µm (C′ for C-F″); 20 µm (C_1_ for C_1_-F_1_).

We asked whether Notch-dependent and -independent her genes have partially redundant functions. We combined the two deficiencies, eliminating all nine *Hes5* homologs together with mutant alleles of *her6* and *her9*, and named embryos homozygous mutant for all 11 her genes ‘her undecimal mutants’ [*her*UDM; for *Df(her4;12),Df(her2;15),her6^−/−^,her9^−/−^*]. The brains of *her*UDM embryos appeared to be severely malformed, and embryos did not survive beyond 120 hpf. When analyzed at 56 hpf (Fig. 10A-B′), *her*UDM embryos had the most severe morphological defects in neural tissues that undergo significant expansion by proliferation on the second day of development, such as the cerebellum, the ventral part of the retina and the rhombic lip. Early forming neural tissues, however, appeared to be mostly normal, which might be because of compensation by other her genes expressed during early stages of development, including *her3*, *her8a* and *her8.2* (Onichtchouk et al., 2010; Webb et al., 2011)*.*

We analyzed Sox2 expression in *her*UDM embryos and observed a severe but variable phenotype, ranging from strong reduction of both the Sox2^high^ NSC and Sox2^low^ NPC compartments (Fig. 10D-D″) to extreme anatomical malformations rendering analysis of the TPZ difficult (not shown). In 72 hpf *her*UDM embryos, the layer of Sox2^high^ nuclei along the walls of the lateral ventricle was completely missing (Fig. 10C″,D″). The Sox2^high^-expressing nuclei along the medial ventricular wall appeared disorganized, and the ventricular layer of Sox2^high^ nuclei was interrupted (Fig. 10C-D″; Movie 4). Some Sox2^+^ nuclei were displaced from the ventricular wall, suggesting that the epithelial integrity of the ventricle may be impaired (Fig. 10C_1_,D_1_, arrows). Similar to *her6*,*her9* double mutants, the Sox2^low^ NPC nuclei were nearly absent (Fig. 10C_1_,D_1_, arrowheads). We still detected pH3^+^ nuclei at the ventricular walls of *her*UDM embryos (Fig. 10D′; Movie 4), suggesting that NSC proliferation is not completely eliminated. The phenotype of *her*UDM is much more severe than of *her6,her9* double mutants with respect to NSC maintenance.

A single *her2;her15* cluster WT allele in *Df(her4;12)* homozygous, *Df(her2;15)* heterozygous, *her6^−/−^,her9^−/−^* mutant larvae partially rescued the *her*UDM phenotype (Fig. 10E-E″; Movie 5, right). The integrity of the ventricular Sox2^high^ nuclear layer was restored and resembled the phenotype in *her6*,*her9* double mutants (Fig. 10E_1_, arrow, E″; Movie 5). As seen in *her6*,*her9* double mutants, the subapical Sox2^low^ nuclei were still absent (Fig. 10E_1_ arrowhead; Movie 5). In contrast, a single *her6* WT allele in *Df(her4;12),Df(her2;15),her6^+/−^,her9^−/−^* mutant larvae fully restored the Sox2^high^ nuclear layer along the lateral ventricular wall (Fig. 10F″ arrowhead; Movie 5, left). The ventricular wall appeared to be intact (Fig. 10F_1_, arrow), and the subapical Sox2^low^ NPC nuclei were present and of normal elongated shape (Fig. 10F′,F_1_, arrowhead). Individual effects of *her9* or *her4,12* were not tested, because they are tightly linked on the *her9 ^m1505^,Df(her4;12)* chromosome 23 we used. When we analyzed *her*UDM rescue in *Df(her4;12)* heterozygous, *Df(her2;15)* homozygous, *her6^−/−^,her9^+/−^* by a single chromosome with WT *her9* and WT *her4;12*, we observed a rescue of the dorsolateral Sox2^high^ nuclei, but only a partial rescue of the Sox2^low^ NPCs in the thalamus (Fig. S15). Thus, a single *her6* allele rescues the *her*UDM phenotype more efficiently than a single WT chromosome with *her9* and the *her4;12* cluster, or the *her2;15* cluster.

We determined whether combined loss of Notch-dependent her genes and of *her9* activity may cause *her6* upregulation in anatomical regions in which it may be cross-repressed in WT. We found that in *Df(her4;12),Df(her2;15),her9* combined mutants *her6* is indeed expressed more broadly along the ventricular wall of the TPZ (Fig. S16). The rescue activity of *her6* in *her*UDM embryos may thus be caused by compensatory upregulation of *her6* expression in areas that in WT do not express high *her6*. In search for the potential origin of these *her6* cells, we analyzed the TPZ at higher resolution, and detected few *her6* cells intermingled in predominantly *her4* and *her9* thalamic regions in WT (Fig. S4D′). A small number of dispersed *her6* expressing cells remains in the ZLI and caudal thalamus even at 48 hpf, when the early broad *her6* expression has recessed from *her4* and *her9* expression domains (Fig. S4F). Expanded *her6* expression in *her*UDM may thus reflect persistence of early *her6* expression, or expansion of small *her6* clones. We hypothesize that much of the compensatory expansion of *her6* expression in *her*UDM happens early in development, given that inhibition of Notch signaling from 64-72 hpf (Fig. 1M) did not cause an expansion of *her6* expression.

In summary, the severe loss of NSCs and NPCs in *her*UDM revealed that, first, the 11 her genes analyzed provide most, if not all, Her activity in the TPZ at 72-96 hpf. Second, Notch-dependent and -independent her genes appear to act partially redundant in NSC maintenance. Third, the Notch-independent *her6* may substitute for other her activity in NSC and NPC maintenance and expansion in the TPZ.

## DISCUSSION

Delta/Notch signaling and HES/Her transcription factors control NSC maintenance and regulate NPC progression in neurogenesis (Kageyama et al., 2007). Their activities differ during primary neurogenesis in the early neuroepithelium of anamniotes, during embryonic brain growth in neural proliferation zones, and in adult neural stem cell niches (Alunni et al., 2020; Kriegstein and Alvarez-Buylla, 2009). Here, we analyzed Her activities in the zebrafish TPZ.

### Notch-dependent and -independent her genes define distinct NSC and NPC populations

We find expression of the *Hes1* homologs *her6* and *her9* to be largely independent of Notch signaling, in line with previous reports on earlier stages (Bae et al., 2005; Hans et al., 2004; Latimer et al., 2005). In contrast, *Hes1* in mice is predominantly regulated by Notch signaling (Hsieh et al., 1997; Jarriault et al., 1995, 1998; Nishimura et al., 1998), but also by Notch-independent input (Kageyama et al., 2007; Ohtsuka et al., 1999). For the zebrafish *Hes5* homologs *her4* (Takke et al., 1999; Zhou et al., 2012), *her12* (Gajewski et al., 2006), *her2* and *her15* (Cheng et al., 2015; Shankaran et al., 2007), we find expression to depend on Notch signaling. Similarly, *Hes5* expression in mice is Notch-dependent (Ohtsuka et al., 1999). In the TPZ, expression of the zebrafish *Hes6* homolog *her8a* depends on Notch signaling, whereas in the neural plate Notch-independent expression has been reported (Webb et al., 2011). *her8.2* appears to be regulated in TPZs by both Notch-dependent and -independent mechanisms.

**Fig. 11.**
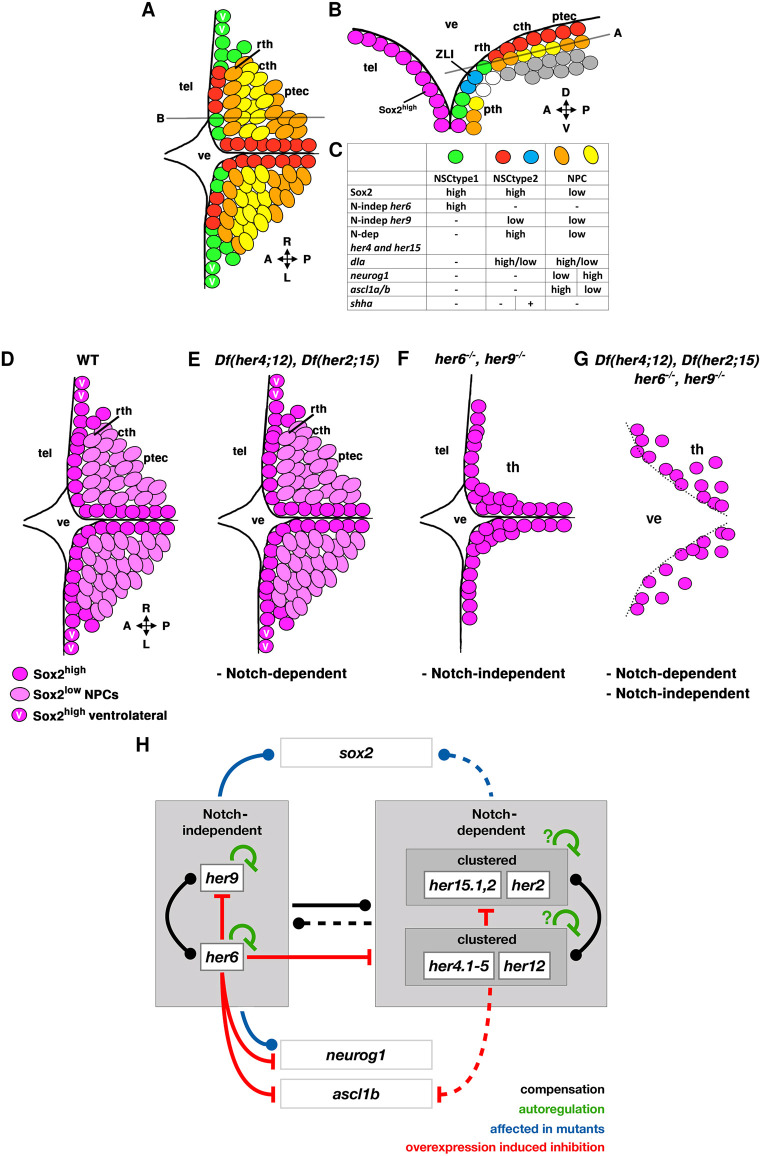
**Cellular organization, her phenotypes and her genetic interactions in the TPZ.** (A-C) Cellular organization in the TPZ. (A) Horizontal plane dorsal view, plane indicated in B, which shows parasagittal section indicated in A. (C) Marker expression. Magenta cells, Sox2^high^ nuclei of the telencephalic ventricular wall, her expression not determined. White cells not analyzed for markers. Gray cells, differentiating early and mature neurons, some of which express neurod family members*.* (D-G) Schematic of TPZ Sox2 expression in WT and mutants as indicated. Sox2^high^ (NSCs) in dark magenta, Sox2^low^ (NPCs) in light magenta. ‘V’ magenta, Sox2^high^ -and *her6* high-expressing cells only present in lateral ventricle wall. Black lines, ventricular surface. Dotted line in G, impairment of ventricle wall integrity. (H) Postulated her gene network interactions. Boxes show analyzed genes and gene expression. Lines indicate how mutations or overexpression influence expression of other genes, interactions may not be direct. Black lines, functional compensation (dot indicates directionality). Dashed black line, partial compensation of *her6* and *her9* loss. Green inhibition signs, autoregulation of *her6* and *her9*, and postulated negative autoregulation in the *her4;her12* and *her2;her15* clusters. Blue influence signs, changed Sox2 and *neurog1* expression in *her6,her9* double mutants. Blue dashed influence sign, loss of Notch-dependent her activity in *Df(her4;12)*,*Df(her2;15)* only affects Sox2 expression in *her*UDMs. Red inhibition signs, downregulation of genes upon overexpression of *her6* or *her4*. Dashed red inhibition sign indicates mild downregulation of *ascl1b* by *her4* overexpression. A-P, anterior-posterior; cth, caudal thalamus; D-V, dorsal-ventral; ptec, pretectum; pth, prethalamus (ventral thalamus), rth, rostral thalamus; tec, tectum opticum; tel, telencephalon; th, thalamus; ve, ventricle.

Differential expression of Notch-dependent and -independent her genes reveals distinct NSC populations in the TPZ (Fig. 11A). We designate Sox2^high^ NSCs expressing high *her6* as NSC type 1, and those expressing high *her4* and *her15* as NSC type 2. Both NSC type 1 and 2 are proliferative, indicating that quiescent versus active NSC populations may only diversify later in development (Than-Trong et al., 2020). The different NSC types emerge during maturation of the thalamic complex, as early *her6* expression comprises the entire presumptive thalamic complex, but becomes gradually restricted (Scholpp et al., 2009). The complementary expression of *Hes1* and *Hes5* homologs in zebrafish is consistent with previous findings on *Hes1* and *Hes5* expression in mice (Hatakeyama et al., 2004).

The role of *her4* during neural plate stages was reported to be similar to Notch-dependent *Drosophila* E(spl) genes, as both mediate repression of neurogenesis in the neuroepithelium by lateral inhibition (Nakao and Campos-Ortega, 1996; Takke et al., 1999). We found that, in the larval TPZ, *her4* and *her15* continue to depend on Notch signaling and are largely co-expressed, but in domains distinct from *her6* expression. A similar dichotomy was observed for the expression of Notch-dependent E(spl) genes versus Notch-independent *hairy* in the fruit fly (Fisher and Caudy, 1998; Nakao and Campos-Ortega, 1996). In the zebrafish neural plate, prepatterning genes mark the inter-proneural domains in a Notch-independent manner (Stigloher et al., 2008). For example, *her3* is expressed in two elongated stripes along the anteroposterior axis, flanked by *neurog1*^+^ progenitor pools (Bae et al., 2000), and *her5* inhibits neurogenesis in the intervening zone at the prospective midbrain-hindbrain boundary (Geling et al., 2004; Ninkovic et al., 2005). These homologs of *Drosophila hairy* appear to act by restricting the proneural domains in the neuroectoderm independent of Notch signaling, similar to *Drosophila hairy* (Fisher and Caudy, 1998; Geling et al., 2004). Similarly, *her6* and *her9* may execute prepattern information to maintain NSCs.

### Her genes in TPZ patterning

Domains of NSC type 1 expressing Notch-independent *her6* and NSC type 2 expressing *her4*, *her15* and *her9* show a strict spatial organization in the TPZ. A central stripe of NSC type 2 cells coincides with the ZLI. Anterior and posterior directly adjacent to the ZLI are narrow stripes of NSC type 1 cells in the prethalamus and rostral thalamus. Further posterior, there is again a stripe of NSC type2 cells in the caudal thalamus. The NSC type 1 *her6* stripes correlate with the *her6-*expressing domain reported to generate *ascl1a* gabaergic progenitors, which has been described both for zebrafish (Scholpp et al., 2009; Scholpp and Lumsden, 2010) and mice (Virolainen et al., 2012). In contrast, the NSC type 2 caudal thalamic domain correlates with the domain generating *Ngn2* glutamatergic progenitors in mice (Virolainen et al., 2012). Shh signaling organizes these domains of the thalamic complex (Scholpp et al., 2006). In *her6* mutants, we find a severe reduction, and in *her6,her9* double mutants complete absence of *shha* expression along the ZLI lateral ventricular walls, while the *her9* mutant ZLI appears normal. Despite *her6* and *her9* being expressed in distinct cell populations, they thus can partially compensate for each other in the ZLI. *her6* and *her9* may execute patterning information to maintain ZLI signaling activity and NSCs. Our analysis of *shha* and *sox2* expression during early development of *her6*,*her9* double mutants reveals that *her6* and *her9* activity are required for dorsal expansion of *shha* expression and establishment of *shha* in the ZLI, which precede later reduction of *sox2* expression in the mutant TPZ. *her6* morphant embryos have been shown to lose *shha* expression in the mid-diencephalic organizer, which was explained by premature differentiation of organizer cells accompanied by the loss of the boundary-characteristic *shha* expression (Scholpp et al., 2009). A similar *Shh* phenotype was observed in *Hes1,Hes5* double mutant mice (Baek et al., 2006). In mice, *Hes1* is expressed at high levels in neural boundaries and organizing centers (Baek et al., 2006), which resulted in a model of two distinct *Hes1* functions. In so-called boundaries, high levels of Hes1 constitutively represses neurogenesis, whereas in compartments its expression oscillates and regulates progression of neurogenesis (Kageyama et al., 2007). Along this concept, *her6* may execute boundary activities. How both types, NSC type 2 *her4*- and *shha*-expressing ZLI organizer cells themselves, and adjacent *her6*-expressing NSC type 1, work together to maintain the organizer should help to understand the complexity of the ZLI.

We can only speculate on upstream regulation of Notch-independent *her6*, but note that *her6* co-expression in high level Sox2-expressing cells suggests a regulatory link to transcriptional networks maintaining NSCs. Further, although *her6* is not expressed in *shha* ZLI organizer cells itself, based on its position it may be subject to Shh and Wnt signaling from the ZLI (Martinez-Ferre et al., 2013). Thus, we hypothesize that *her6* may be controlled by combined NSC regulatory networks and ZLI signaling.

### Notch-dependent and -independent her functions in NPCs

Surprisingly, in *Df(her4;12),Df(her2;15)* double mutants, Sox2^high^ and Sox2^low^ NPCs, as judged by expression of *ascl1a/b*, *neurog1* and *olig2* in the TPZ, appear to be largely normal. Thus, in the TPZ, these nine Notch-dependent her genes together appear to be expendable for the initiation of proneural gene expression in NPCs. Our *her4* overexpression experiments support this interpretation: in *her4* heat-shocked embryos, expression of *neurog1* and *neurod1/6b* are largely normal, and of *ascl1b* in most embryos only slightly reduced. Together, these findings are surprising, because a strong effect of Notch-dependent her genes on proneural gene expression would be expected (Imayoshi et al., 2013; Takke et al., 1999).

The lack of a phenotype suggests that Notch-dependent her gene activity either may have only a minor contribution to NSC maintenance or be substituted by Notch-independent her gene activity. The rather normal development is also in stark contrast to the severe phenotypes observed previously in studies using other methods for inhibition of canonical Notch signaling. *mib1* mutants are a paradigm for loss of Delta signaling and show a severe phenotype (Itoh et al., 2003). However, MIB1-mediated ubiquitylation has been shown to target also other cellular functions, including ciliogenesis (Wang et al., 2016). LY-411575 inhibits gamma-secretase, which has more than 80 targets other than Notch (Haapasalo and Kovacs, 2011). The severe *mib1* and LY-411575 phenotypes may thus be enhanced by effects on mechanisms other than Notch signaling. It also needs to be considered that our combined Notch-dependent her mutants target the larval TPZ, but do not affect other Notch-dependent her genes broadly expressed in earlier development, like the *Hes6* homologs *her8a* and *her8.2.* Finally, inhibition of Notch signaling inhibits all Notch targets, whereas our study selectively investigates those effects mediated by her genes. Thus, our combined analysis of Notch-dependent her genes is likely to be valid for larval TPZ, but does not address earlier embryonic stages or other Notch signaling activities.

In contrast, loss of Notch-independent *her6* and *her9* results in early premature differentiation and smaller and disorganized domains of *ascl1a/b*, *neurog1* and *olig2* in the 72-96 hpf TPZ. Overexpression of *her6* eliminates *neurog1* and *ascl1b* expression, and causes a reduction of *neurod1/d6b* expression, revealing an influence on NPCs entering neuronal differentiation. Similar to our observations, *Hes1* single and *Hes1,Hes5* double mutants have much stronger neurogenesis phenotypes than *Hes5* mutants (Hatakeyama et al., 2004). Our findings so far suggest a strong contribution of Notch-independent her gene activity to control of NPC development, either at the level of generating new NPCs from NSCs or NPC differentiation. As *her6* is not expressed in NPCs, we favor the hypothesis that Notch-independent her genes mostly affect generation of new NPCs. This could be by maintaining or increasing the NSC pool, or by controlling NSC-to-NPC transition.

### Notch-dependent and -independent her functions in NSCs

In contrast to combined deficiencies of Notch-dependent her genes, *her6,her9* double mutants exhibit a strong NSC phenotype with absent Sox2^high^ NSC nuclei in the lateral extensions of the ventricle, and a significant overall reduction in the total NSC count in the TPZ (Fig. 11D-F). Even more severely, combined inactivation of Notch-dependent and -independent her genes in *her*UDMs causes loss of most NSCs and essentially all NPCs in the TPZ, and loss of ventricular wall integrity (Fig. 11G). The *her*UDM phenotype in severity appears similar to the *Hes1,Hes5* double mutant phenotype in mice with premature differentiation and stem cell depletion (Baek et al., 2006; Hatakeyama et al., 2004; Ohtsuka et al., 1999). The severe *her6,her9* double mutant phenotype reveals a prominent role in NSC maintenance or proliferation. Our pH3 analysis reveals that loss of Notch-dependent or -independent her genes does not affect NSC proliferation, supporting a prevalent role of Notch-independent her genes in NSC maintenance. The efficient suppression of proneural genes, as observed after *her6* overexpression, may be crucial for NSC maintenance.

### Her gene network interactions

*her6* and *her9* show negative feedback auto-regulation and cross-regulation; however, the latter was largely masked in the presence of functional autoregulation, such that the expression of both genes is most strongly upregulated in their double mutants (Fig. 11H). Overexpression of *her6* repressed *her9*, *her4* and *her15*, revealing that Notch-independent *her6* repression in a sense is dominant over these her genes. Due to the cluster deletions, we could not investigate autoregulation of Notch-dependent her genes in mutants. *her4* overexpression downregulates *her15*, but did not affect *her6* and *her9* expression*,* confirming that these two genes are indeed independent of Notch signaling. Accordingly, *her6* and *her9* expression is largely unaffected in *Df(her4;12),Df(her2;15)* double mutants. Redundancy of her genes in zebrafish is a recurring motif that has been shown for *her1*, *her7* and *her13.2* in somitogenesis (Henry et al., 2002; Oates and Ho, 2002; Sieger et al., 2006), as well as *her5* and *her11* (*him*) at the mid-hindbrain boundary (Ninkovic et al., 2005), but also for *Hes1* and *Hes3* in mice (Hirata et al., 2001).

When we analyzed individual her genes with respect to their rescue potential in *her*UDM mutants, a single WT allele of *her6* rescued depletion of the activity of 21 other her gene alleles in *her*UDM embryos*.* This special activity of *her6* may be explained by two mechanisms: First, we observed that in *Df(her4;12),Df(her2;15),her9* mutants, *her6* expression in the TPZ is not restricted to the rostral thalamus and prethalamus, which may be interpreted as persistent expression of *her6* from the broad early thalamic complex *her6* domain (Scholpp et al., 2009). Second, although in WT high level expression domains of *her6* versus *her4/her9*-expressing subregions of the TPZ are largely exclusive, we observed few high and some low *her6*-expressing cells also intermingled in *her4* territories. Thus, in the *her6*-rescued *her*UDM, *her6*^+^ clones may expand. The prominent role of *her6* may thus be rooted in its early broad expression in the TPZ*.*

### Conclusions

In summary, the expanded number of *Hes1/Hes5* homologous genes in zebrafish goes along with a clear separation of Notch-dependency for different her genes. Our genetic analysis demonstrates that the her gene network is largely redundant within the Notch-dependent and -independent her groups, and that the latter may better substitute for the loss of Notch-dependent genes than vice versa. The observed robustness of the network is supported by several regulatory feedback loops and cross-regulation, and makes NSC maintenance robust against severe perturbations of her activity. In the TPZ, Notch-independent her genes predominantly regulate NSC maintenance and contribute to ZLI formation and *shha* expression. With respect to neurogenesis, we hypothesize that *her6* may control lineage transitions, e.g. from NSC to NPC, whereas *her4* may rather modulate progression of neurogenesis.

## MATERIALS AND METHODS

### Zebrafish maintenance and breeding

ABTL strain zebrafish (*Danio rerio*) were kept and bred under standard conditions (Westerfield, 2000). Experiments were performed in accordance with the German Animal Welfare Guidelines. All animal experiments were approved by the ethics committee for animal experiments at the state authority Regierungspräsidium Freiburg (permits 35-9185/G-16/123, 35-9185.81/G-19/19 and 35-9185.81/G-19/54).

Zebrafish embryos were obtained through natural breeding and embryos were raised in E3 medium (5 mM NaCl, 0.17 mM KCl, 0.33 mM CaCl_2_, 0.33 mM MgSO_4_, supplemented with 2 ppm Methylene Blue). We added 0.2 mM N-Phenylthiourea (PTU, Sigma-Aldrich) to the E3 medium to prevent pigmentation. Embryos were staged as previously described (Kimmel et al., 1995).

### Embryo fixation and storage

Embryos were incubated in E3 (containing 0.2 mM PTU) supplemented with 1× tricaine methanesulfonate (MS-222) and fixed in 4% paraformaldehyde (PFA) for 4 h at room temperature (RT) or overnight at 4°C. Embryos were washed several times with PBST (137 mM NaCl, 2.7 mM KCl, 10 mM Na_2_HPO_4_, 1.8 mM KH_2_PO_4_, 1% Tween-20) and dehydrated stepwise with 25%, 50% and 75% MeOH in PBST, and stored at −20°C in 100% MeOH.

### Genotyping

DNA was isolated from embryos or tail biopsies of fixed larvae. Samples were heated 5 min to 95°C in 60 µl TE buffer, after addition of 3 µl Proteinase K (20 mg/ml), and digested for at least 4 h at 55°C. After heat-inactivation of Proteinase K for 10 min at 95°C, 3 µl of the DNA sample was used in 30 µl PCR volume. The primer concentration was 0.3 µM unless stated otherwise. PCR programs are supplied in Table S4. For genotyping of embryos used in the qPCR, an alkaline lysis was performed. Tails were cut and transferred to 40 µl 50 mM NaOH and incubated for 45 min at 95°C. The lysis was stopped by addition of 4 µl 1 M Tris-HCl (pH 7.5).

### Generation of transgenic lines

New transgenic lines were generated using the Tol2 kit vector system (Kwan et al., 2007). For heat shock-driven *her4* and *her6* overexpression, *hsp:her4.1-FLAG* and *hsp:her6-FLAG* were cloned by combining a 1.5 kb *hsp70 l* heat shock promoter fragment (Chi-Bin Chien, Genbank AF158020) with the *her4.1* (ENSDART00000079274.4) or *her6* (ENSDART00000023613.9) cDNA sequence (from EZRC: kit numbers KIT001605 and KIT000178; www.ezrc.kit.edu) with a FLAG-tag (DYKDDDDK or 5′-GACTACAAAGACGATGACGACAAG-3′) in a vector containing Tol2 sites and a green heart marker (*clmc2:EGFP*). The *her4.1* gene was chosen for overexpression of *her4* activity because the *her4.1* open reading frame (ORF) represents the consensus amino acid sequence of all five *her4* cluster genes (alignment of *her4.1* NP_001096598, *her4.2* AAH49296, *her4.3* NP_001154880, *her4.4*, NP_001121862, and *her4.5* NP_571165). The sequence of the Her4.1 protein we cloned diverges from NP_001096598 only at amino acid 49 valine, which is Her4 consensus and shared with Her4.2 and 4.5. Thus, we assume that the Her4.1 ORF in our transgene has full Her4 activity. The following alleles were used in this study: *Tg(hsp70l:her4.1-FLAG)m1541, Tg(hsp70l:her6-FLAG)m1492.*

### Cas9 mRNA and sgRNA generation

Cas9 mRNA was transcribed from the hspCas9 plasmid (Ansai and Kinoshita, 2014) and Cas9-nanos from the Cas9-nanos-3′-UTR plasmid (Moreno-Mateos et al., 2015) using the SP6 mMessage mMachine kit (Thermo Fisher Scientific), and a polyA tail was added (PolyA tailing kit from Thermo Fisher Scientific). sgRNAs were designed with CRISPRscan (Moreno-Mateos et al., 2015) and ordered as oligonucleotides to generate DNA templates (Gagnon et al., 2014). Annealing of the constant oligonucleotide with the variable oligonucleotide was carried out by cooling from 95°C down to 25°C in 1 h in a PCR machine. Annealed oligonucleotides were filled with T4 DNA Polymerase (New England Biolabs) for 20 min at 11°C. sgRNAs were transcribed for 3-4 h at 37°C using the MEGAscript T7 kit according to protocol (Thermo Fisher Scientific) and dissolved in 15 µl H_2_O.

### Targeted induction of knockouts and large deficiencies

#### *her6 m1358* mutant allele

Two sgRNAs (transcribed from p3-Oligo04 and p4-Oligo05) were used to generate the *her6* knockout allele *m1358*. The intragenic deletion generates a frameshift that truncates the protein after amino acid 36 before the bHLH domain. Lower case letters are deleted in the mutant allele *m1358*: 5′TATAGTGCTTCAAGAagtgattagacagattgactcattttgtttttattttgcattttagtcttctaaacccattatggagaaaagaagaagagcgagaatcaacgaaagcttgggtcagctgaaaacgttaatcttggatgctctgaaaaaagatgtaagtaccgaaagtccgactgagtctttcagttaggctatatctggctggaatattaatgcatttctttatatagccgcatatctaaatcagtctctttcttcactctcagagctccagacactctaaacttgagaaagccgacATCCTGGAGATGACA3′.

#### *her9 m1368* mutant allele

Two sgRNAs (transcribed from p186-Oligo26 and p187-Oligo27) were used to generate the *her9* knockout allele *m1368.* The intragenic deletion generates a frameshift that truncates the protein after amino acid 18 before the bHLH domain. Lower case letters are deleted in the mutant allele *m1368*: 5′ATTGCTGGTGCCCCTgccagtggatctcatactcctgacaagccaaagaatgccagcgagcatagaaagtcttcaaagccaatcatggaaaagcgccgcagagcgagaatcaacgagagccttgggcagctgaagactctcattcttgatgctcttaaaaaagatagctccagacactctaaattggagaaagctgatattctggagatgacagtcaagcacctgcgcaatttacaacgtgttcagatgagcgcagccttgtcagctgacacaaacgtcctcagcaagtaccgcgcaggattcaacgagtgcatgaacgaggtgactcgatttctctctacctgcgagggagtgaatacagaggtcagatcgcgacttcttaaccacctgtCCGGTTGTATGGGAC3′.

#### *Df(her2;15)* m1490

Two sgRNAs (transcribed from p211-Oligo38 and p219-Oligo43) were used to generate the 30 kb deficiency *Df(Chr11:her2,her15.1,her15.2)*, which we abbreviate to *Df(her2;15)* (Fig. S12). We injected 1 nl of the injection mix (6.5 µl total volume), which consisted of 1 µl of each sgRNA (transcribed from Oligo p211 and Oligo p43) plus 4.5 µl Cas9-nanos mRNA (500-800 ng/µl), into one-cell-stage zebrafish embryos. Single embryos at 24 hpf were genotyped using PCR with two primers p213+p222 (0.3 µM each) to test for knockout events (Table S2). The PCR was performed using MyTaq polymerase (Bioline; PCR program in Table S4). If embryos showed the desired deletions, siblings were raised and crossed to ABTL fish. F1 embryos carrying deletions were identified by PCR and siblings were raised to adulthood. Adult F1 fish were fin clipped and genotyped with primers p213+p222 (0.3 µM each)+p220 (0.08 µM) (Tables S2 and S4). Stable heterozygous F2 lines were established and appeared to grow normally.

#### *Df(her4;12)* m1364 and m1365

Two sgRNAs (transcribed from p39-Oligo12 and p47-Oligo16) were used to generate the 28 kb deficiency *Df(Chr23:her12,her4.1,her4.2,her4.3,her4.4,her4.5)*, which we abbreviate to *Df(her4;12)* (*m1364* and *m1365* are shown in Fig. S12). We injected 1 nl of the injection mix (8.3 µl total volume), which consisted of 1 µl of each sgRNA (transcribed from Oligo12 and Oligo16) plus 6 µl hspCas9 mRNA (600-1200 ng/µl), into one-cell-stage zebrafish embryos. Then 24 hpf single embryos were genotyped by PCR with p42+p51 (0.3 µM each) to test for knockout events (Table S2). The PCR was performed using MyTaq polymerase (Bioline; PCR program in Table S4). If embryos showed deletions, siblings were raised and crossed to ABTL fish. F1 embryos were identified by PCR and siblings were raised to adulthood. Adult F1 fish were fin clipped and genotyped with primers p42+p43+p51 (0.3 µM each) (Tables S2 and S4). Stable heterozygous F2 lines were established and appear to grow normally.

#### Her activity depleted *Df(her2;15), Df(her4;12), her4, her6* (herUDM) strain

The *her9* gene (chromosome 23: 23,399,520-23,401,305) is only 1.9 Mb away from *her4.5* (chromosome 23: 21,469,203-21,471,022), which is its closest neighbor in the *her4* gene cluster. To obtain the combined *her6*,*her9*, *her4;12* cluster and *her2;15* cluster mutant strain (*her*UDM), *Df(her4;12) m1364* heterozygous embryos were injected with sgRNAs (transcribed from p186-Oligo26 and p187-Oligo27) to target *her9* on the *Df(her4;12)* chromosome. For this mutant, 1 nl of the injection mix (5 µl total volume), which consisted of 1 µl of each sgRNA and 3 µl Cas9-nanos mRNA (500-800 ng/µl), was injected into one-cell-stage zebrafish embryos. The establishment of stable F2 lines was similar to that described for the large deficiency mutants. The PCRs were performed with primer p197 and p198 and the program can be found in Table S4. The allele was named *her9 m1505.* Lower case letters are deleted in the mutant: 5′TGCTGGTGCCCCTGCcagtggatctcatactcctgacaagccaaagaatgccagcgagcatagaaaggtaaaaatcacttataatgcgattgcatattgttttagaagaatgacagctgcatcagttattctaaaaaaagagagaatgtcttttaattacgtaaattaattaattttccatttcatctgcagtcttcaaagccaatcatggaaaagcgccgcagagcgagaatcaacgagagccttgggcagctgaagactctcattcttgatgctcttaaaaaagatgtaagttttatctacaactcttgtcatgcttcagtcacccgacgttagtaattctgaaacagtttctaaccgaattctgctcattcacagagctccagacactctaaattggagaaagctgatattctggagatgacagtcaagcacctgcgcaatttacaacgtgttcagatgagcggtaagttgcaagtcagattcctcaagatgataaacttttaacgtgcttttaaaaacgcaatttaatttcctgaatacacaatctaaatacgttttatctttgtttaccagcagccttgtcagctgacacaaacgtcctcagcaagtaccgcgcaggattcaacgagtgcatgaacgaggtgactcgatttctctctacctgcgagggagtgaatacagaggtcagatcgcgacttcttaaccacctGTCCGGTTGTATGGG3′. When breeding the *her9 m1505, Df(her4;12)* chromosome, we also checked for unwanted crossing over events by PCR for the *Df(her4;12)* allele.

### LY-411575 treatment

For pharmacological inhibition of Notch signaling by LY-411575 (Rothenaigner et al., 2011), embryos were incubated in E3 supplemented with 0.2 mM PTU until 64 hpf. Then embryos were incubated in 1× E3 supplemented with 0.2 mM PTU, 2% DMSO, 10 µM LY-411575 for 8 h. Control embryos were treated in 1× E3 supplemented with 0.2 mM PTU and 2% DMSO for 8 h in the same six-well plate as the LY-treated embryos. Embryos were checked for vital functions and fixed at 72 hpf in 4% PFA.

### NICD overexpression

For the overactivation of the Notch signaling pathway, *Tg(hsp:Gal4;UAS:RFP) kca4Tg* were crossed with *Tg(UAS:NICD)kca3Tg* (Scheer and Campos-Ortega, 1999).This allowed heat shock-controlled overexpression of NICD. At 64 hpf, embryos were heat shocked for 30 min at 40°C in pre-heated E3 (with 0.2 mM PTU). Heat-shocked embryos were then sorted for the heat shock-induced RFP marker. Non-fluorescent embryos were used as negative controls. Embryos were tested for the *UAS:NICD* element by PCR (Tables S2 and S4).

### Whole-mount immunofluorescence

Fixed and stored embryos were rehydrated with 75%, 50%, 25% MeOH in PBST and washed several times with PBST. Embryos were digested with Proteinase K (10 µg/ml) in PBST for 45 min at RT. Embryos were re-fixed with 4% PFA for 20 min at RT and then washed several times with PBST. Larvae were blocked in blocking solution [1% bovine serum albumin (BSA), 5% goat serum, 1% DMSO in PBST] for 1 h and incubated with primary antibodies diluted in blocking solution. The anti-Sox2 antibody (20G5, Abcam, ab171380, LOT GR3253929-5; cross-reacts with both zebrafish Sox2 and Sox3 proteins; Westphal et al., 2022) and the anti-phospho-Histone H3 (Ser10) antibody (Merck, 06-570, LOT 3319395) were diluted 1:400. Embryos were incubated in primary antibodies overnight at 4°C and then washed several times for 30 min each with PBST supplemented with 1% DMSO. Embryos were incubated with the secondary antibodies 1:1000 in PBST supplemented with 1% DMSO and 1% blocking reagent (Roche, 1096176) overnight at 4°C. Secondary antibodies were Alexa 555 goat anti-mouse IgG (Thermo Fisher Scientific, A-11001) and Alexa 488 goat anti-rabbit IgG (Thermo Fisher Scientific, A-11070). Embryos were washed several times in PBST, transferred to 80% glycerol in PBST and stored at 4°C protected from light. The embryos were recorded soon after staining.

### RNA detection by HCR

Whole-mount *in situ* hybridization by HCR was performed as described in the HCR v3.0 protocol for zebrafish embryo and larvae (Choi et al., 2018) and by Molecular Instruments. The following changes to the protocol were made. In preparation stage 11, 72 hpf larvae were treated with proteinase K (30 µg/ml) for 30 min. In detection stage 1, 15 larvae were used in 2 ml tubes. After amplification stage 6, embryos were washed twice in PBST for 5 min and stored in 80% glycerol in PBS. Probes for *neurog1* (ENSDART00000078563.5), *asc1a* (ENSDART00000056005.5), *ascl1b* (ENSDART00000183550.1), *sox2* (ENSDART00000104493.5) and *her6* (ENSDART00000023613.9) were ordered and designed by Molecular Instruments.

We designed probe sets for *her4* (ENSDART00000079274.4), *her9* (ENSDART00000078936.4), *her15* (ENSDART00000055707.6) and *olig2* (ENSDART00000060006.5) (Table S3). For example, the *olig2* probe set consists of ten probe pairs which are evenly distributed along the mRNA sequence (Table S3). One probe pair consists of two gene-specific 25 bp sequences each with a GC content of 37-85% and a Tm of 55-77°C. The two sequences were separated by a gap of two nucleotides. The B1 specific spacer and initiator sequences were added to the reverse complement of the two gene-specific sequences. The complete probe set was ordered from Sigma-Aldrich. Equal volumes of all 20 single probes were mixed and used as probe mixture containing 5 µM of each probe in detection stage 3.

### Generation of *in situ* hybridization probes

Specific her probes were designed to avoid cross-hybridization of conserved domains. For the generation of *her6*, *her9*, *her4.1-her4.5*, *her15.1-15.2*, *her2* and *her12* whole-mount *in situ* hybridization probes, sequences spanning the last exon and/or 3′ UTR of the respective genes were amplified by PCR (all primers for probe generation are given in Table S2). The PCRs were performed on WT genomic DNA with PfuUltra II (Agilent). The 50 µl PCR mix contained 6-10 µl dNTP mix (2.5 mM each), 5 µl 10× Pfu buffer, 0.2 mM of each primer, 2 µl DNA and 1 µl PfuUltra II Polymerase. Before cloning, A-overhangs were added by incubating 20 µl of the purified PCR product with 9.7 µl MyTaq buffer, 0.2 µl MyTaq polymerase and 0.1 µl (25 µM) dATPs for 20 min at 72°C. After amplification, the probes were cloned into a TOPO vector according to the manufacturer's protocol (TOPO TA cloning kit, Thermo Fisher Scientific).

For the *her4* probe, which recognizes *her4.1* (ENSDART00000079274.4), *her4.2* (ENSDART00000137573.2), *her4.3* (ENSDART00000104209.4), *her4.4* ENSDART00000079265.6) and *her4.5* (ENSDART00000104206.4), a template was generated with primer p159F and p160R (annealing 57°C, 40 s). For the *her15* probe, which recognizes *her15.1* (ENSDART00000055707.6) and *her15.2* (ENSDART00000055706.6), a template was cloned using primers p157F and p158F (annealing 56°C, 40 s). The *her2* (ENSDART00000055709.5) probe template was amplified with p154F and p155R (annealing 56°C, 40 s) and the *her12* (ENSDART00000044080.7) probe template was amplified with p152F and p153R (annealing 56°C, 40 s). The *her6* (ENSDART00000023613.9) probe template was amplified using p147F and p148R (annealing 57°C, 55 s) and the *her9* (ENSDART00000078936.4) probe template was amplified with p149F and p150R (annealing 56°C 40 s). All primers are given in Table S2.

The *her8a* (ENSDART00000123395.4) probe template was amplified as published in Webb et al. (2011), with p322F and p327R (annealing 56°C, 1 min) and the *her8.2* (ENSDART00000101578.4) probe template was amplified with p260F and p259R (annealing 56°C, 1 min). For *her8a* and *her8.2* cDNA was used as a template in the PCR.

*sox2* (ENSDART00000104493.5), *neurod1* (ENSDART00000011837.6), *neurog1* (ENSDART00000078563.5), *shha* (ENSDART00000149395.3), *neurod6b* (ENSDART00000185805.1), *irx1b* (ENSDART00000079114.6; Scholpp et al., 2009), *ascl1a* (ENSDART00000056005.5; Allende and Weinberg, 1994), *ascl1b* (ENSDART00000183550.1; Allende and Weinberg, 1994) probes have been previously published (www.zfin.org) and plasmids were validated by sequencing.

Plasmids were linearized and transcribed with T7 or SP6 RNA Polymerases (Thermo Fisher Scientific). Antisense RNA probes were generated using the DIG- or DNP-based RNA labeling kits from Roche (Merck, Germany). The quality of the RNA probe was checked by agarose gel electrophoresis.

### Whole-mount *in situ* hybridizations

Chromogenic whole-mount *in situ* hybridizations were performed as previously described (Holzschuh et al., 2003). After fixation, storage and rehydration, embryos were washed three times in PBST and treated with 10 µg/ml Proteinase K (AppliChem, 15 min incubation per 24 h of development). Embryos were washed with PBST and fixed in 4% PFA for 20 min at RT. Embryos were washed five times in PBST and incubated in hybridization mix (50% formamide, 5× SSC, 5 mg/ml torula yeast RNA type IV, 50 µg/ml heparin, 0.1% Tween-20) at 65°C for 4 h. Next, embryos were incubated with digoxigenin-labeled antisense RNA probes in hybridization mix (dilution 1:200-1:500) at 65°C overnight. After hybridization, embryos were washed at 65°C for 20 min each with the following solutions: twice with 50% formamide in 2× SSCT, twice with 25% formamide in 2× SSCT for 20 min each, twice in 2× SSCT for 20 min each, three times in 0.2× SSCT for 20 min each. Next, embryos were washed once in 0.1× SSCT in 0.5× PBST for 10 min and twice in PBST for 10 min each at room temperature. Embryos were blocked for 2-3 h with 2% heat inactivated goat serum (Vector Laboratories) and 4 mg/ml BSA (AppliChem) in PBST. Embryos were incubated overnight at 4°C in blocking solution supplemented with the alkaline phosphatase-coupled anti-digoxigenin antibody (Roche, 11093274910, 1:3000). Embryos were washed six times in PBST at room temperature for 20 min each and three times in NTMT [100 mM NaCl, 100 mM Tris-HCl (pH 9.5), 50 mM MgCl_2_, 0.1% Tween-20] for 15 min each. For the staining reaction, 0.18 mg/ml BCIP (5-bromo-4-chloro-3-indolyl-phosphate, AppliChem) and 0.45 mg/ml NBT (4-nitroblue tetrazolium chloride, AppliChem) were added to NTMT. Staining was performed in the dark and stopped by three washes in PBST supplemented with 0.1 mM EDTA. For longer storage, embryos were fixed in 4% PFA for 1 h at room temperature. After three more washes in PBST, embryos were transferred to 80% glycerol in PBST supplemented with 0.1 mM EDTA and stored at 4°C protected from light.

### Fluorescent whole-mount *in situ* hybridizations

Fluorescent *in situ* hybridizations (FISH) were performed as described above (‘Whole-mount *in situ* hybridizations’) until addition of probes. The digoxigenin- and dinitrophenol-labeled antisense probes (see ‘Generation of *in situ* hybridization probes’) together were added to the hybridization mix and the embryos were incubated at 65°C overnight. Washes were performed at 65°C as described above. Embryos were washed in TNT [100 mM Tris-HCl (pH 7.5), 150 mM NaCl, 0.5% Tween-20] and blocked in TNT supplemented with 1% Boehringer Block (Roche) for 2-3 h at RT. The anti-digoxigenin-POD Fab fragment antibody (Roche, 11207733910) was used 1:400 in blocking solution and embryos were incubated overnight at 4°C. Embryos were washed eight times for 15 min each in TNT at room temperature, rinsed in 100 mM borate buffer (pH 8.0) and incubated in staining mix containing 2% dextrane sulfate, 1% tyramide stock solution Alexa Fluor 488 (Thermo Fisher Scientific), 0.0015% H_2_O_2_ and 112.5 µg/ml 4-iodphenole in 100 mM borate buffer (pH 8.0) for 1 h at room temperature. Embryos were washed three times in TNT, incubated in 0.3% H_2_O_2_ in TNT for 30 min and then washed five times in TNT for 5 min each at room temperature. Embryos were blocked in 1% blocking reagent (Roche) in 100 mM maleic acid, 150 mM NaCl (pH 7.5) for 2-3 h at RT. The anti-DNP HRP antibody (Perkin Elmer, FP1129) was used at 1:200 in blocking solution and embryos were incubated overnight at 4°C. Embryos were washed eight times for 15 min each in TNT at room temperature, rinsed in 100 mM borate buffer (pH 8.0) and incubated in staining mix containing 2% dextrane sulfate, 1% tyramide stock solution Alexa Fluor 555 (Thermo Fisher Scientific), 0.0015% H_2_O_2_ and 112.5 µg/ml 4-iodphenole in 100 mM borate buffer (pH 8.0) for 1 h at RT. Embryos were washed three times in TNT and three times in PBST for 5 min each at room temperature. Embryos were transferred to 80% glycerol in PBS and stored at 4°C protected from light.

### Microscopy and cell counts

Imaging of chromogenic whole-mount *in situ* hybridizations was performed with the Zeiss Axioskop2, Axiovision SE64, Rel.4.9.1 with AxioCam ICc1 with a Plan-Neofluar 20×/0.5 or 10×/0.3 objective and DIC optics. Embryos were mounted in 80% glycerol in PBST supplemented with 0.1 mM EDTA or in 80% glycerol in H_2_O supplemented with 1% standard agarose (Bioron). All shown images are single *z*-planes from an image stack.

Imaging of fluorescent stainings was performed with an inverted microscope with LSM-880 Airyscan (Zeiss). A 40× objective LD LCI Plan Apochromat 40×/1.2 autocorr (Zeiss) was used. Embryos were embedded in 80% glycerol in H_2_O supplemented with 1% standard agarose (Bioron). The immersion medium was glycerol. The imaging program ZEN 2.3 SP1 FP3 (black) version 14.0.22.201 was used. If Airyscan was used, image stacks were processed with the ZEN black software using the Airyscan algorithm (v. 2.3 SP1 Zeiss). All images shown are single *z*-planes from an image stack, except for Fig. 2A,B,C; Fig. 4A,B,C,C″,D,D″; Fig. 7A,B,C,C″,D,D″, Fig. 10C,D,E,F and Fig. S3A,B, which show orthogonal projections of an image stack. Fig. 2D,D′ and D″ show single *z*-planes from an image stack of laterally mounted embryos.

Counts of cell nuclei (Fig. 7E-E′; Table S7) were performed on single confocal image planes for consecutive optical sections representing similar anatomical regions of the TPZ for each embryo analyzed. The TPZ regions for analysis were chosen on the dorsal-most plane in which Sox2^low^ cells were visible and Sox2^high^ cells present at the ventricle. The same regions were analyzed also on the following nine more-ventral *z*-planes (optical distance of *z*-planes 2 µm). pH3^+^ nuclei were individually marked and counted using ImageJ2 (v2.9.0/1.53t) with the analyze plugin Cell Counter. pH3^+^ nuclei extending over more than one focal plane were included only once in the count of the central focal plane of an individual nucleus. The Sox2^+^ nuclei are densely packed and assignment of single planes in *z* to a single nucleus is difficult. The average diameter of a nucleus in this region is 4-5 µm. We counted each nucleus in each plane. At 2 µm optical plane distance, we therefore estimate that each nucleus may be counted ∼2-3 times. Sox2^low^ versus Sox2^high^ nuclei were distinguished and counted based on distinctly different relative levels of immunofluorescence intensity in each focal plane.

The assignment of pH3^+^ nuclei to Sox2^low^ versus Sox2^high^ was difficult based on the fact that pH3^+^ nuclei are in mitosis, and during mitosis the nuclear envelope resolves, such that the Sox2 immunoreactivity in the cell does not reveal a distinctly bright nucleus. Therefore, if the Sox2 level could not be identified non-ambiguously, we assigned pH3^+^ nuclei in the Sox2^+^ region of the TPZ to Sox2^low^ or Sox2^high^ based on the following three criteria: (1) nuclei surrounded on three out of four sides by Sox2^low^ nuclei were considered Sox2^low^; (2) nuclei surrounded on three out of four sides by Sox2^high^ nuclei were considered Sox2^high^; (3) nuclei located at the ventricular surface and on both sides at the ventricular wall between Sox2^high^ nuclei were considered Sox2^high^. The counts of Sox2^high^ nuclei may in addition be affected by interkinetic nuclear migration. However, as M-phases typically occur at the apical side, this should not affect our evaluation of pH3 nuclei counts in Sox2^high^ cells. For comparison of mitotic activities, we calculated the fraction of pH3 nuclei among Sox2^high^ and Sox2^low^ nuclei counts. Due to multiple counts of individual Sox2^+^ nuclei (but not of pH3 nuclei, see above), we estimate that the mitotic index may be 2-3 times higher than the pH3/Sox2^+^ count ratio. Statistical analysis and graphs were generated using Prism 9 for macOS Version 9.5.0 (525).

Figures were assembled in Photoshop (version 13.0). Image levels were linearly adjusted except for Fig. 5B-C′,F-G′, Fig. S8 and Fig. S10A-F′ to improve visibility in very dark stained areas.

### Numbers of embryos analyzed

For WISH experiments analyzing WT, compound-treated or heat shock overexpression-treated embryos, shown in Fig. 1G-R′, Fig. 8, Fig. 9, Fig. S1, Fig.S2G-J′ and Fig. S14, for each condition 10-20 larvae were examined for their expression patterns and representative images are shown. When the numbers (*n*) are provided in images or legends, the number indicates how many larvae were imaged. If staining patterns or intensity varied within one experimental condition (Fig. 9B′,E′,J′), one embryo with a staining pattern representative for the majority of embryos analyzed is shown, and the numbers in the bottom right corner indicate the number of embryos with the representative expression pattern and the total number of embryos analyzed for this condition.

For analysis of genetic mutants or fluorescent WISH shown in Fig. 1A-F′, Fig. 2, Fig. 3C-N′, Fig. 4, Fig. 5, Fig. 6C-F′, Fig. 7, Fig. 10, Fig. S2A-D′, Fig. S3, Fig. S8, Fig. S9, the numbers (*n*) of embryos imaged for each condition are provided in the figure legends or in the image panels, and in Table S5.

### qPCR

The tail of each 96 hpf larva was cut and transferred to a new tube for genotyping as described above. The rest of each larva was stored individually in 75 µl RNA later solution (Ambion, AM7024) on ice. Following genotype detection by PCR, total RNA was extracted from 2-3 embryos pooled per genotype using the RNeasy Mini Kit (Qiagen, 74106) and QIAshredder columns (Qiagen, 79656). The sample was homogenized in 1% β-mercaptoethanol in 600 µl RLT buffer (Qiagen, 74106). The suspension was transferred to a QIAshredder column and centrifuged for 1 min at 10,000 ***g***. Then 700 µl 70% ethanol was added to the flow through and transferred to RNeasy Mini spin columns. After centrifugation (15 s, 10,000 ***g***), 350 µl RW1 buffer (Qiagen, 74106) was added to the membrane and centrifuged. A digestion step with DNase I was performed by adding 80 µl of the DNase I incubation mix (consisting of 10 µl DNase I stock solution with a concentration of 2.73 Kunitz units/µl and 70 µl RDD buffer; Qiagen, 74106) to the RNeasy spin column membrane for 15 min on the benchtop to remove DNA from the membrane. After two more washing steps according to the manufacturer's protocol, the column was dried by centrifugation for an additional 2 min. Total RNA was eluted by adding 30 µl H_2_O to the filter membrane. Tubes were incubated for 1 min at RT and centrifuged for 1 min at 10,000 ***g***. The RNA concentrations were determined by NanoDrop measurements.

We used 100 ng total RNA as a template for reverse transcription into cDNA. cDNA was generated with the SuperScript III RT Kit according to the manufacturer's protocol using oligo(dT)_12-18_ (0.5 µg/µl) primers. The qPCR was performed with the SYBR Green Supermix (Sso Advanced Universal, Bio-Rad). The 10 µl qPCR mix contained 5 µl 2× SYBR Green Supermix, 1 µl primer mix (0.5 µM final concentration of each primer) and 2 µl cDNA. The PCR plate was carefully sealed and centrifuged for 10 min at 1077 ***g***. qPCRs were performed with the LightCycler (Roche), and programs are given in Table S4. The data analysis was performed as in Taylor et al. (2019), and *actb2* was used as reference gene. We note that for calculations of relative expression, we excluded one of the three biological replicates for the double mutant, as the CT value for *actb2* control in this replicate was off. Therefore, the number of biological replicates for *her6* and *her9* qPCR in *her6,her9* double mutants is two. For all others the number of biological replicates is three. Statistical analysis was carried out using Microsoft Excel 2016.

## Supplementary Material

10.1242/develop.201301_sup1 Supplementary information
