# Supplementary material for: A network of Notch-dependent and -independent her genes controls neural stem and progenitor cells in the zebrafish thalamic proliferation zone
Source: Development. 2023 Apr 3;150(7):dev201301. doi: 10.1242/dev.201301 (PMC10112928; doi:10.1242/dev.201301)
Supplement: Supplementary information [file develop-150-201301-s1.pdf]

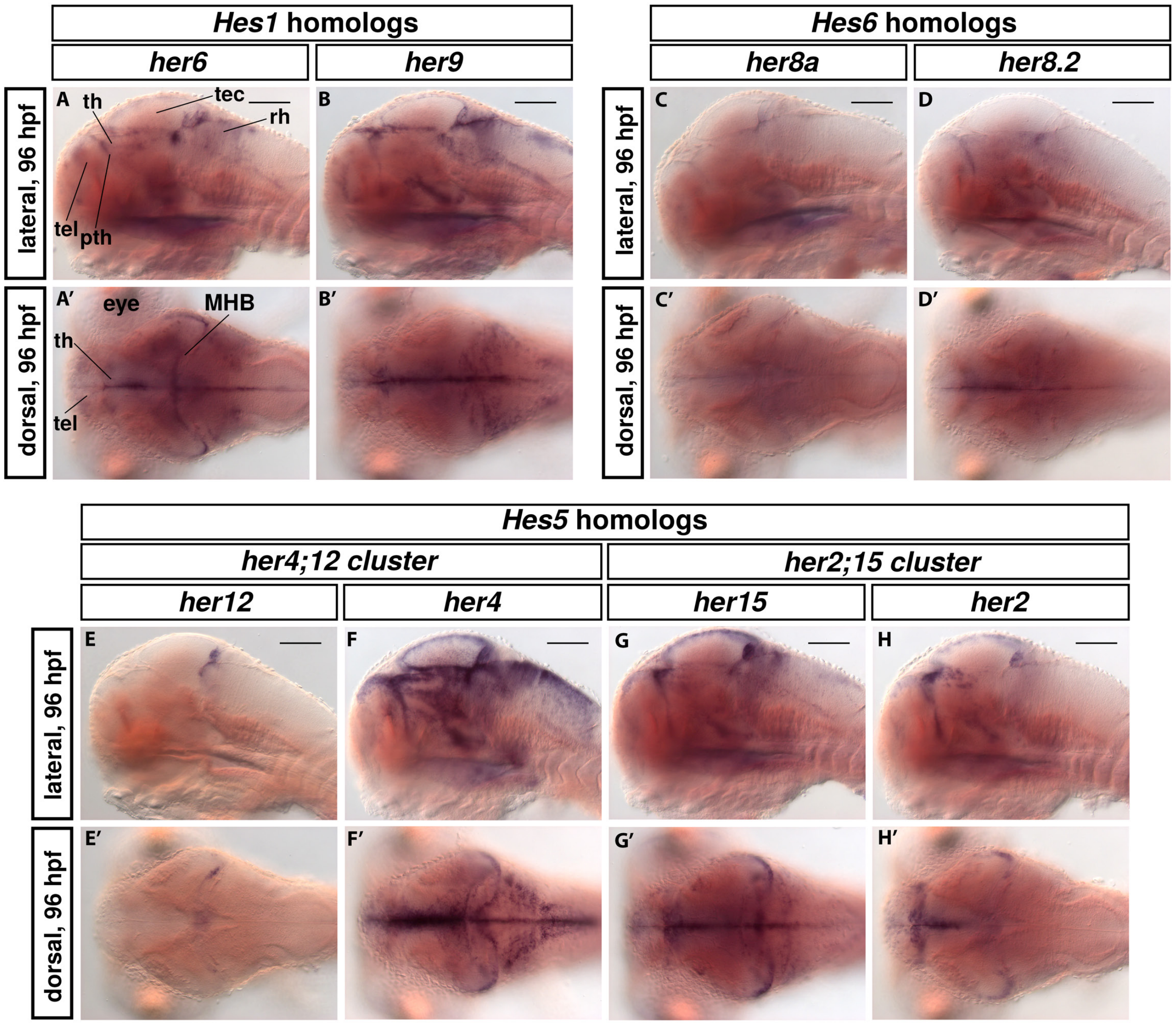

Fig. S1. *her* gene expression in 96 hpf zebrafish larvae.

(A-H') Expression of *her6*, *her9*, *her8a, her8.2, her12, her4, her15* and *her2* in 96 hpf zebrafish larvae visualized by WISH. Top rows show sagittal midline optical sections (A-H). Bottom rows show dorsal views of single horizontal planes at the level of the thalamus (A'-H'). **(A-B')** *Hes1* homologs. **(C-D')** *Hes6* homologs. **(E-H')** *Hes5* homologs. The *her4* probe detects *her4.1-her4.5* and the *her15* probe detects *her15.1* and *her15.2*. For each probe, three larvae were imaged and consistent WISH staining patterns observed. Abbreviations: MHB, midbrain-hindbrain boundary; pth, prethalamus; rh, rhombencephalon; tec, tectum; tel, telencephalon; th, thalamus proper. Scale bars, 100 µm.

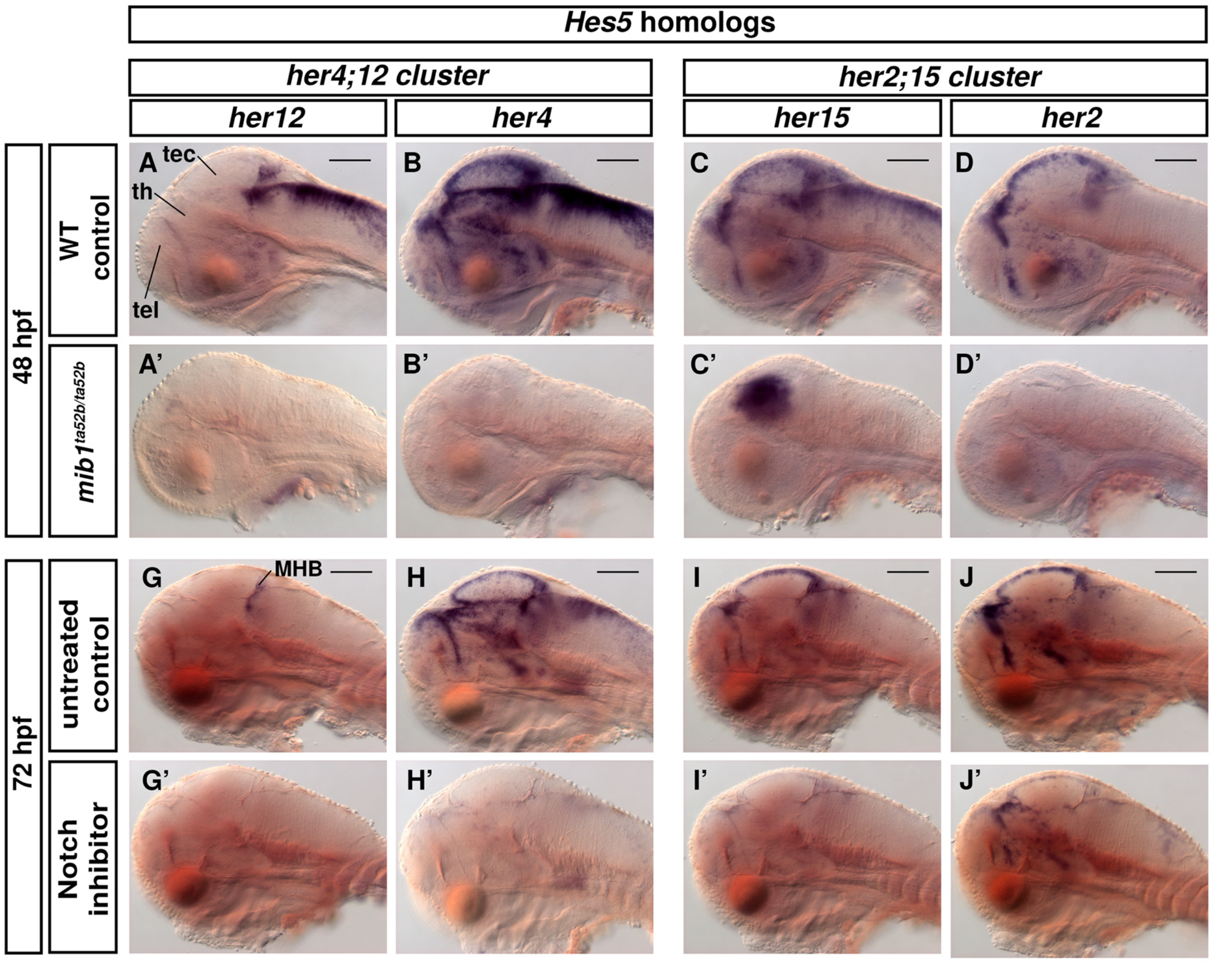

Fig. S2. Expression of *Hes5* homologs after interference with Notch signaling.

(A-J') Expression of *her12, her4, her15* and *her2* visualized by WISH, lateral views. **(A-D')** 48 hpf *mind bomb* mutants ($mib1^{ta52b}$) in comparison with control siblings ($mib1^{+/+}$ or $mib1^{+/ta52b}$). **(G- J')** *her* gene expression after LY-411575 Notch inhibition in comparison with DMSO treated controls. Larvae were treated for 8 hours with 10 µM LY-411575 or 2% DMSO, respectively, and fixed at 72 hpf. **(A-J')** Single sagittal midline image planes are shown. Three larvae per condition were imaged and consistent WISH staining patterns observed. The *her4* probe detects *her4.1-her4.5* and the *her15* probe detects *her15.1* and *her15.2*. Scale bars, 100 µm. Abbreviations: MHB, midbrain-hindbrain boundary; tec, tectum; tel, telencephalon; th, thalamus proper.

Development • Supplementary information

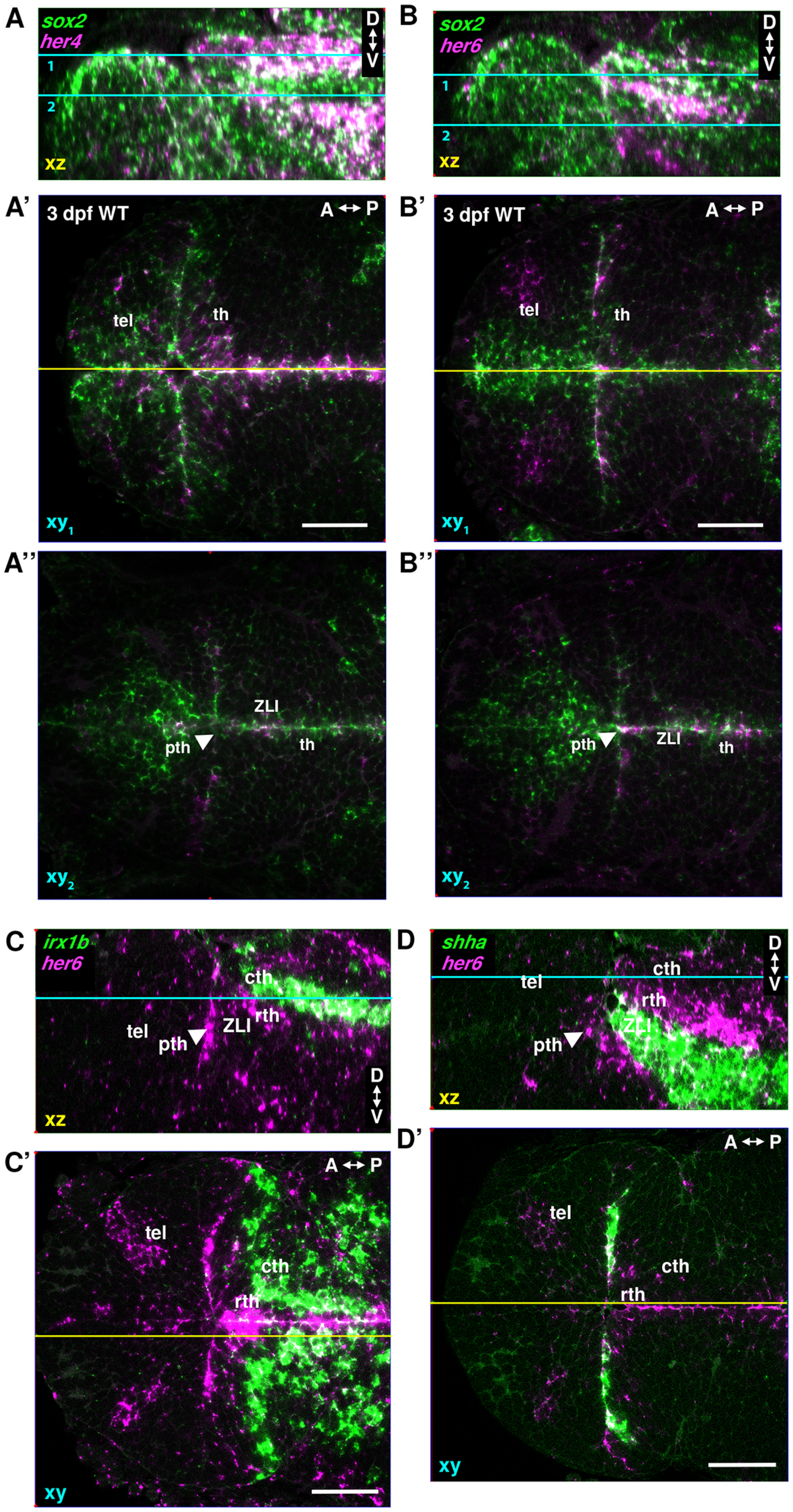

Fig. S3. Expression analysis of *her4* and *her6* in relation to *sox2* and anatomical markers. **(A-A'')** Co-expression of *sox2* (green) and *her4* (magenta), **(B-B'')** co-expression of *sox2* (green) and *her6* (magenta), **(C,C')** expression of *irx1b* as marker for caudal thalamus (green) and *her6*

Development • Supplementary information

(magenta), and **(D,D')** expression of *shha* as marker for ZLI (green) and *her6* (magenta) visualized by double-fluorescent WISH. **(A, B, C D)** Midsagittal planes (orthogonal reconstructions from dorsal view confocal stacks) with cyan lines indicating the horizontal confocal image planes 1 and 2 shown in A', A'' and B', B'', C' and D', respectively. The yellow lines in A' to D' indicate the sagittal planes of A to D. A' and B' are dorsal views of the thalamus proper at the level of line 1 shown in cyan in A and B. A'' and B'' are dorsal views of the prethalamus at the level of line 2 shown in cyan in A and B. The *her4* probe detects *her4.1-her4.5* and the *her15* probe detects *her15.1* and *her15.2*. Scale bars, 50 µm. Numbers of embryos analyzed: A, n=3; B, n=3; C, n2=; D, n=2.

Development • Supplementary information

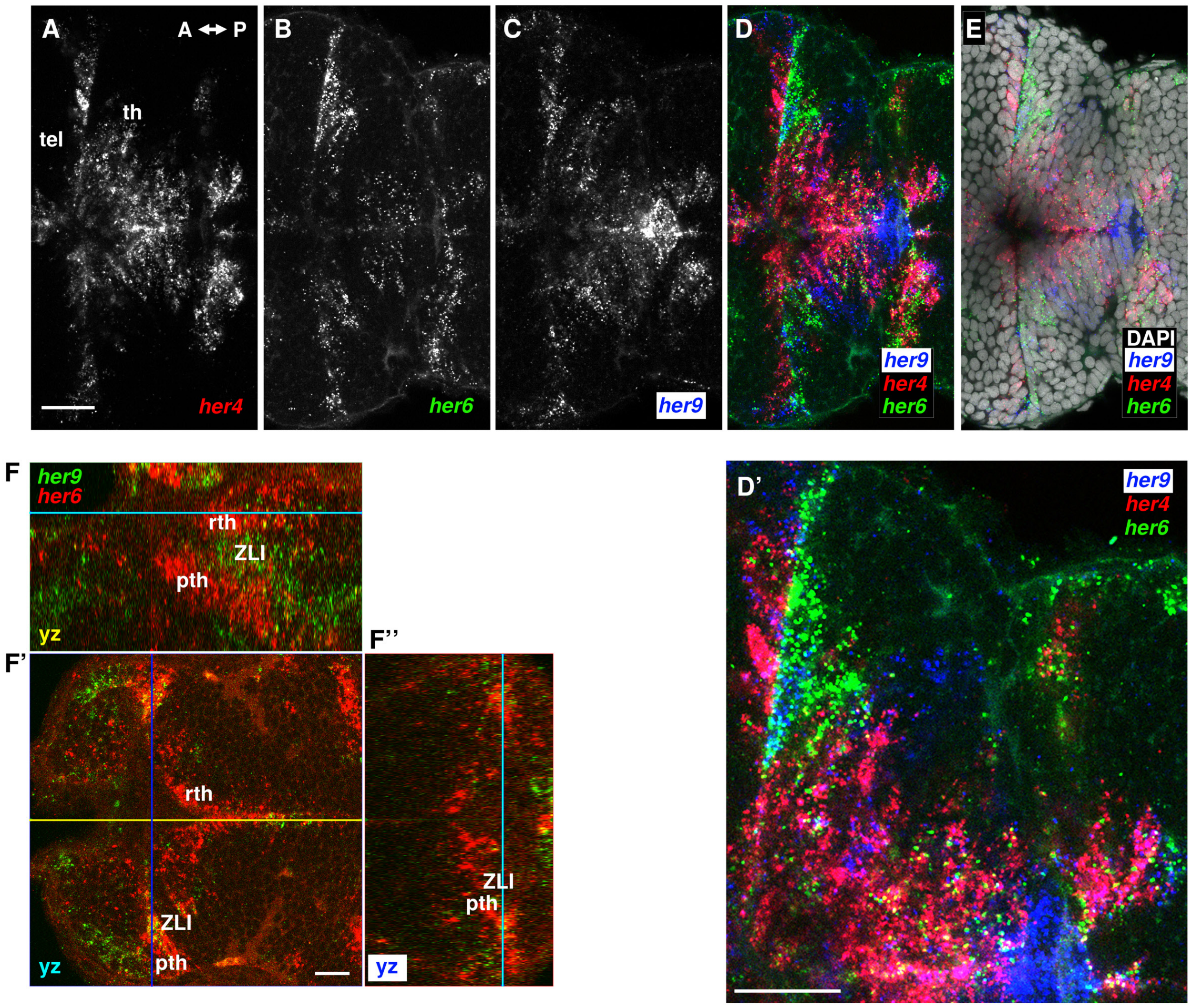

Fig. S4. TPZ expression of *her4, her6* and *her9* in relation to each other.

(A-E) *her4, her6* and *her9* co-detection by fluorescent whole mount hybridization chain reaction (HCR) in WT 72 hpf embryos. Single confocal plane with focus on the dorsal thalamus. A, *her4*; B, *her6*; C, *her9*; D, merge; D' merge magnification of right half of TPZ shown in D; E, merge with nuclear stain (DAPI). Two embryos were analyzed. **(F)** *her6* and *her9* co-detection by HCR in WT 48 hpf embryos. F shows midsagittal orthogonal YZ reconstruction (yellow line in F'), F' a horizontal XY plane (cyan line in F), and F'' a frontal orthogonal reconstruction (blue line in F'). Scale bars in A, D', F' 20 µm. Numbers of embryos analyzed: A-E, D' n=2; F, n=2. Abbreviations: A-P indicates anterior-posterior orientation; tel, telencephalon; th, thalamus proper.

Development • Supplementary information

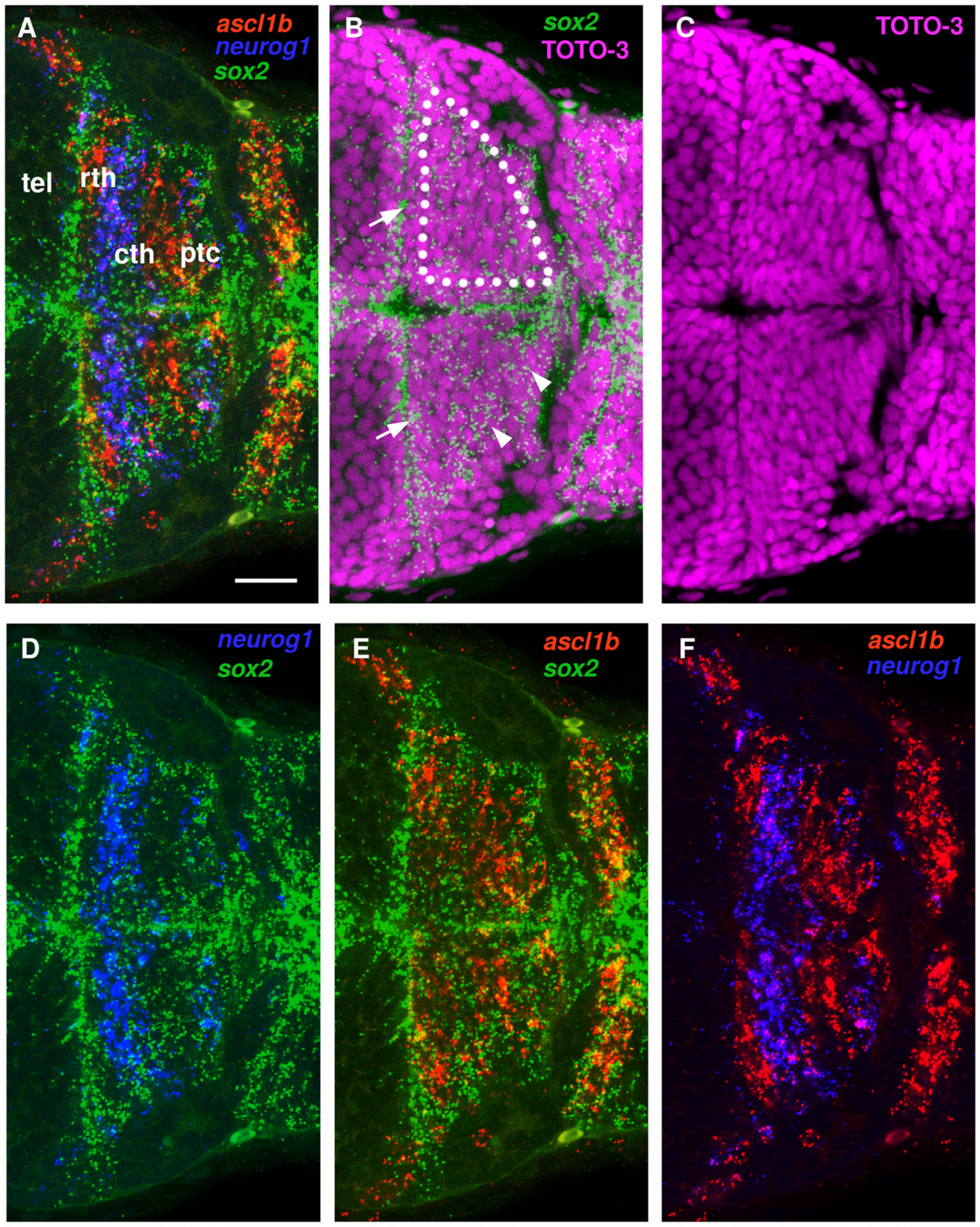

Fig. S5. Analysis of NPC marker expression reveals *sox2*^{low} expressing cells as progenitors.

(A-F) Triple probe HCR-RNA FISH showing *ascl1b* (red), *neurog1* (blue) and *sox2* (green) expression in the thalamus of a 72 hpf WT larva. **(A)** In *sox2^{low}* cells, *sox2* appears co-expressed with *neurog1* or *ascl1b*. **(B)** Nuclear stain with TOTO-3 reveals the location of nuclei relative to *sox2* mRNA. The dotted lines mark the area of *sox2*^{low} expressing cells in the thalamus proper. Arrowheads mark *sox2*^{low} expressing cells. Arrows mark ventricular *sox2*^{high} expressing cells, with most *sox2* HCR signal apical to the nuclear layer at the ventricular surface. **(C)** The extent of the ventricular layer of nuclei (and cells) can be estimated from the TOTO-3 staining. **(D)** *neurog1* and *sox2* are co-expressed in *sox2^{low}* cells in the caudal thalamus. **(E)** *ascl1b* and *sox2* are co-expressed in *sox2^{low}* cells in the rostral thalamus. **(F)** *ascl1b* and *neurog1* are expressed at high levels in largely separated domains, but with partial overlap of low expression in the thalamus proper. Images A-F show the same embryo and horizontal confocal image plane as in Fig. 4C' with color channels linearly adjusted to emphasize distinct and overlapping expression domains (Supplementary Movie M1). Scale bar in A, 20 µm. Three embryos were analyzed. Abbreviations: cth, caudal thalamus; ptc, pretectum; rth, rostral thalamus; tel, telencephalon.

Development • Supplementary information

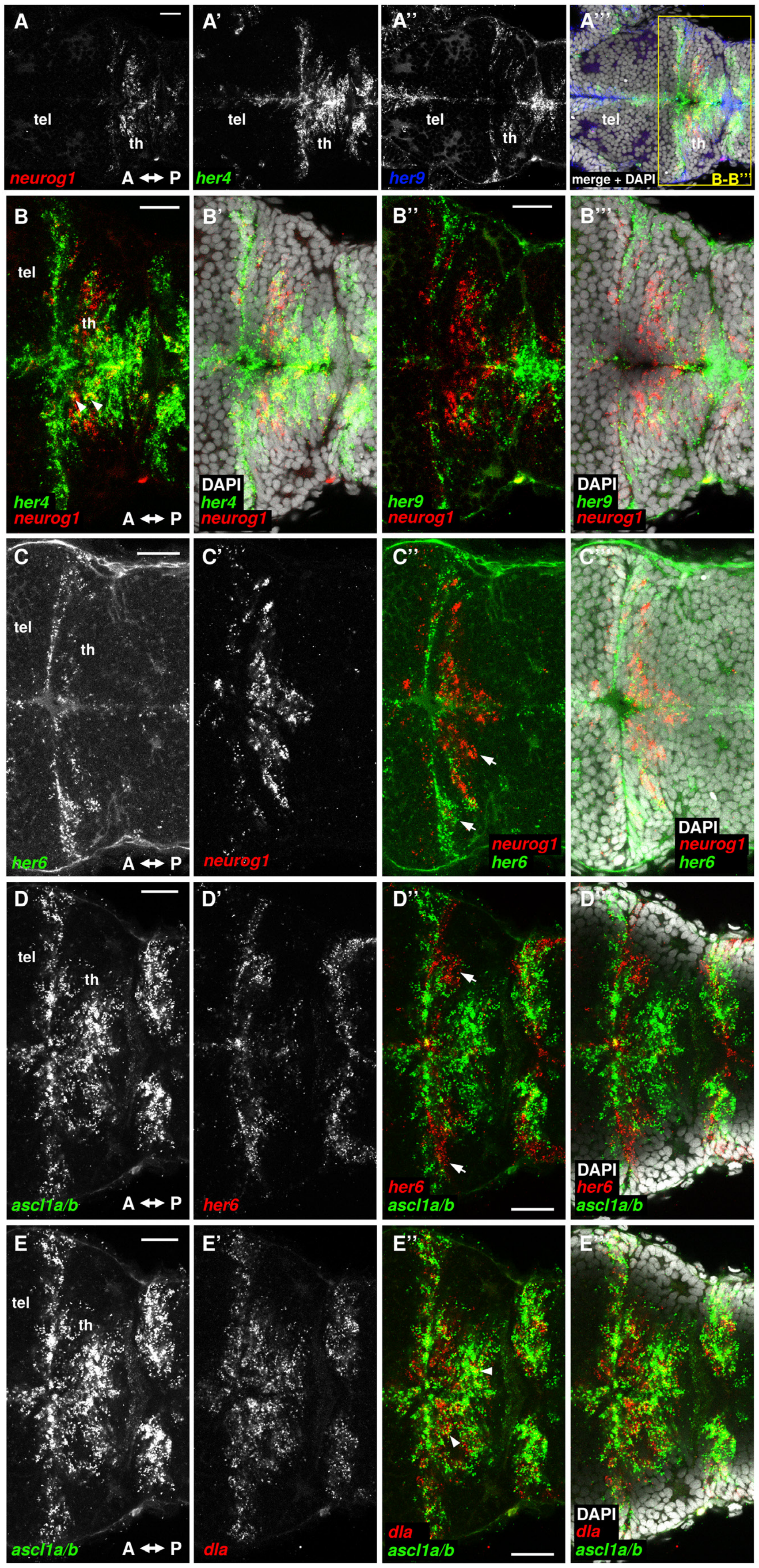

Fig. S6. Expression analysis of *her* genes in relation to proneural genes at 72 hpf.

Development • Supplementary information

(A-E''') Whole mount hybridization chain reaction (HCR), confocal horizontal image planes at level of thalamus proper. **(A-A''')** Overview of triple HCR for *neurog1* (A, red in A'''), *her4* (A', green in A''') and *her9* (A'', blue in A''') and merged with DAPI stain (A'''). **(B'-B''')** Magnifications of the yellow rectangle in A''' with focus on the dorsal thalamus. B and B' show overlapping expression domains of *neurog1* (red) and *her4* (green) in the thalamus (arrowheads). B'' and B''' show largely non-overlapping expression of *neurog1* and *her9*. **(C-C''')** *neurog1* (red) and *her6* (green) show largely non-overlapping expression (arrows). **(D-D''')** *ascl1a/b* (green) and *her6* (red) also show largely non-overlapping expression (arrows). **(E-E''')** *ascl1a/b* (green) and *dla* (red) show overlapping expression domains (arrowheads). DAPI nuclear stain (white) as indicated. Three embryos each were analyzed per HCR combination. Scale bars in A, B, C, D and E, 20 µm. Abbreviations: A-P anterior-posterior orientation; tel, telencephalon; th, thalamus proper.

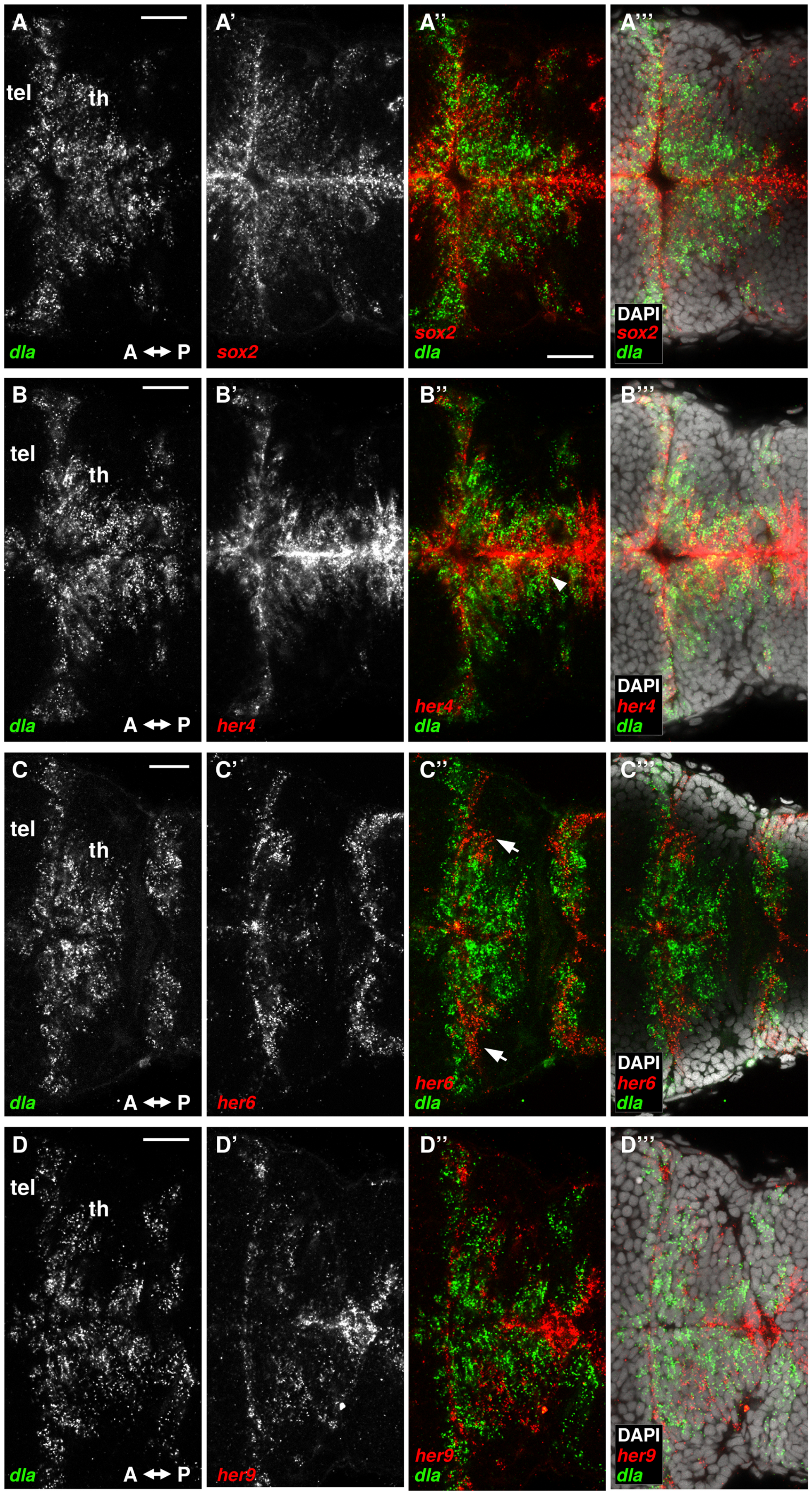

Fig. S7. Expression analysis of *her* genes in relation to *dla*.

(A-D''') Whole mount hybridization chain reaction (HCR) confocal horizontal image planes at level of thalamus proper. **(A-A''')** Overlapping expression domains of *sox2* (red) and *dla* (green). **(B-B''')** Mostly overlapping expression domains of *her4* (red) and *dla* (green) (arrowhead). **(C-C''')** *dla* (green) and *her6* (red) have largely non-overlapping expression (arrows). **(D-D''')** *dla* (green) and *her9* (red) show mostly non-overlapping expression. DAPI nuclear stain (white) as indicated. Numbers of embryos analyzed: A to C n=3 each; D n=2. Scale bars, 20 µm. Abbreviations: A-P anterior-posterior orientation; tel, telencephalon; th, thalamus proper.

Development • Supplementary information

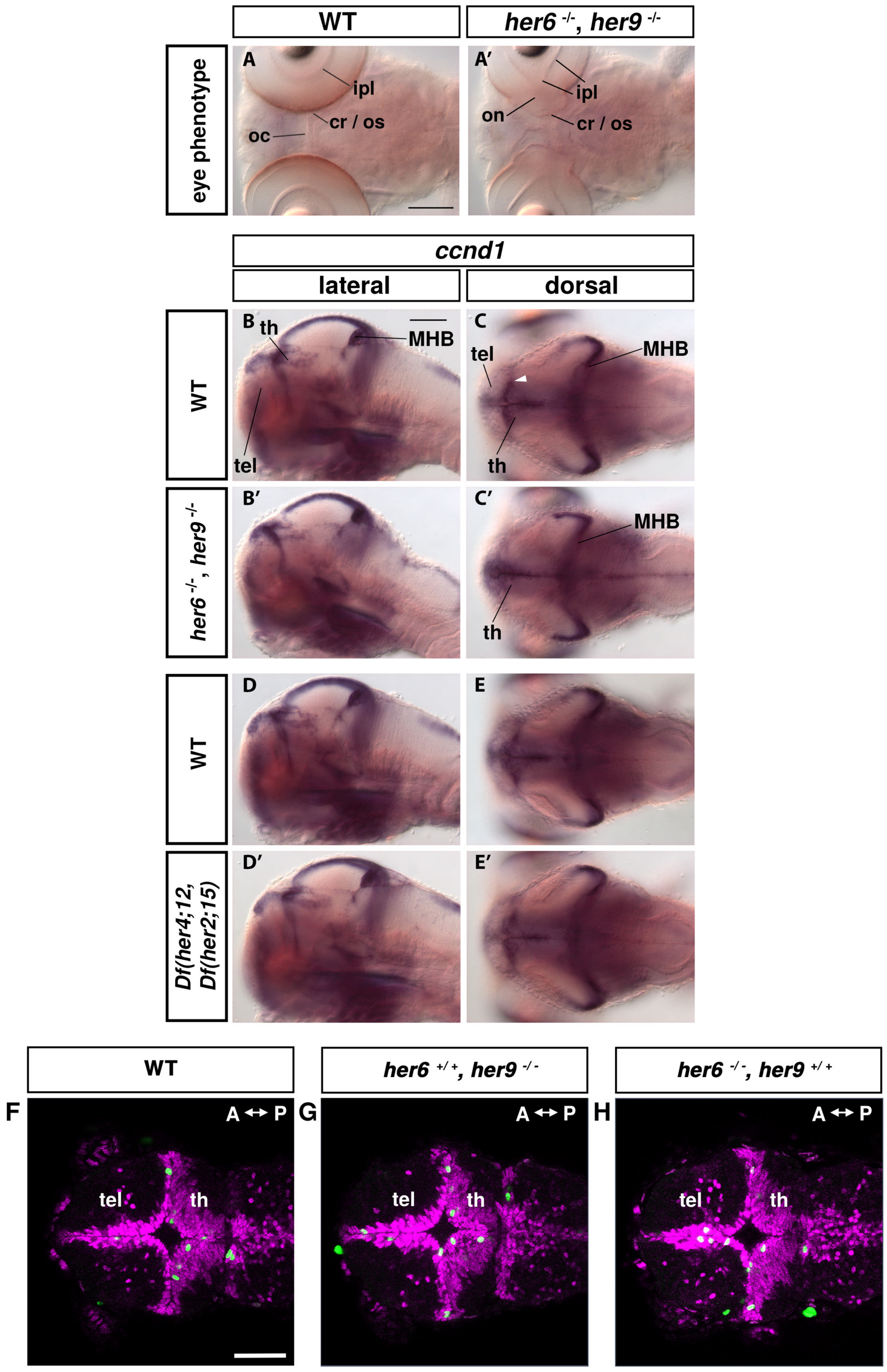

Fig. S8. Morphological, proliferation and neural stem cell phenotypes in *her6, her9* single and double mutants as well as *Df(her4;12)*, *Df(her2;15)* double deficiency mutants.

(A,A'), Morphological eye phenotype in *her6, her9* double mutant embryos. Dorsal views of 4 dpf fixed embryos, DIC optical section. *her6*, *her9* double mutant with malformations at the central retina and optic stalk. Abbreviations: cr / os, central retina / optic stalk; ipl, inner plexiform layer; oc, optic commissure; on, optic nerve. The phenotype has high penetrance and is observed in all double mutant embryos. **(B-E')** *ccnd1* expression in 96 hpf wild type and mutant embryos visualized by WISH, genotypes as indicated. In the lateral views, single midline sagittal DIC optical sections are shown (B, B', D, D'). In the dorsal views, single horizontal DIC optical sections at the level of the thalamus are shown (C, C', E, E'). At least three larvae were imaged per condition and one representative image was selected. Anterior at left, dorsal at top. Scale bar 100 µm for all images. Abbreviations: MHB, midbrain-hindbrain boundary; tel, telencephalon; th, thalamus proper. **(F-H)** Anti-Sox2 and anti-pH3 immunofluorescence in 72 hpf embryos, genotypes as indicated. Scale bar in F (for F-H) 50 µm; Number of embryos analyzed: n=1.

Development • Supplementary information

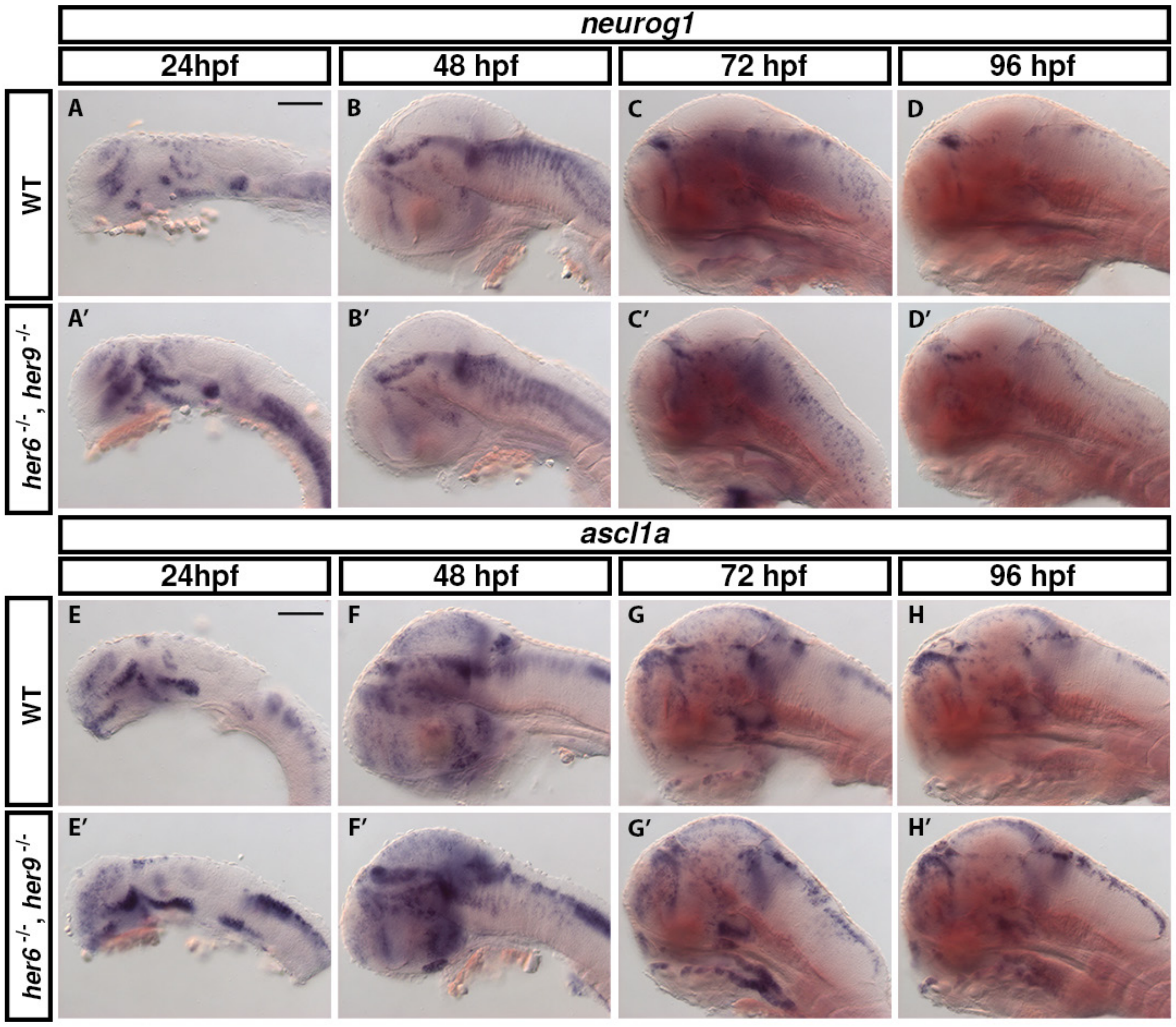

Fig. S9. Proneural gene expression in 24 hpf to 96 hpf *her6,her9* double mutants

(A-H') Expression of proneural genes from 24 hpf to 96 hpf. **(A-D')** *neurog1* expression in WT A-D and double mutants (A'-D'). **(E-H')** *ascl1a* expression in WT (E-F) and double mutants (E'- F'). At least three larvae were imaged per condition and one representative image was selected. Analyzed genotypes showed consistent WISH expression patterns. Sagittal optical sections close to midline, anterior at left, dorsal up; scale bar in A for A-D and in E for E-H, 100 µm.

Development • Supplementary information

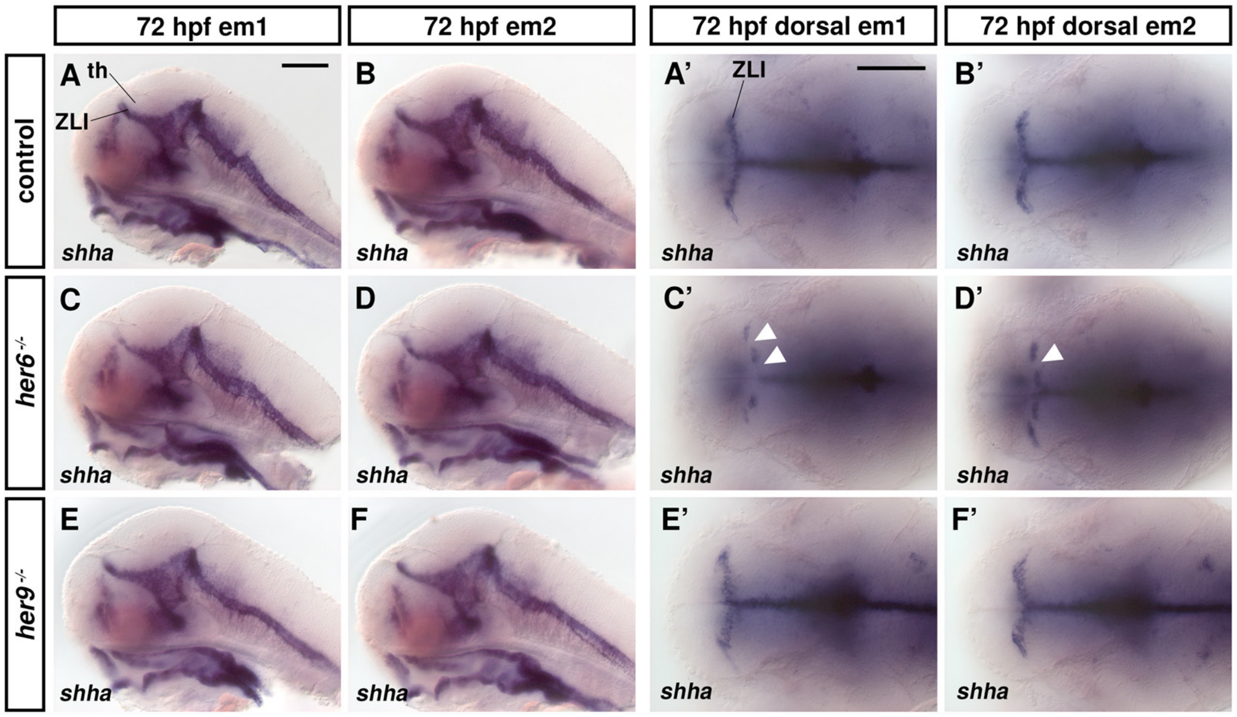

Fig. S10. *shha* expression in *her6* and *her9* single mutants.

(A-F') WISH of *shha* in control (heterozygous for *her9*) and *her6* or *her9* single mutants as indicated. **(A-F)** Lateral view midsagittal optical sections of *shha* expression in 72 hpf larvae. **(A'-F')** Dorsal view horizontal optical sections of *shha* expression in 72 hpf larvae at the level of the thalamus. Three larvae were imaged per condition and two representative images (em1 and em2) were selected since phenotypes of different severity were observed in *her6*$^{-/-}$ single mutant embryos (C' and D'). A and A' as well as B and B' show different embryos each. Anterior at left, dorsal at top. Scale bar in A is 100 µm for A-F; scale bar in A' is 100 µm for A'-F'. th, thalamus proper; ZLI, zona limitans intrathalamica.

Development • Supplementary information

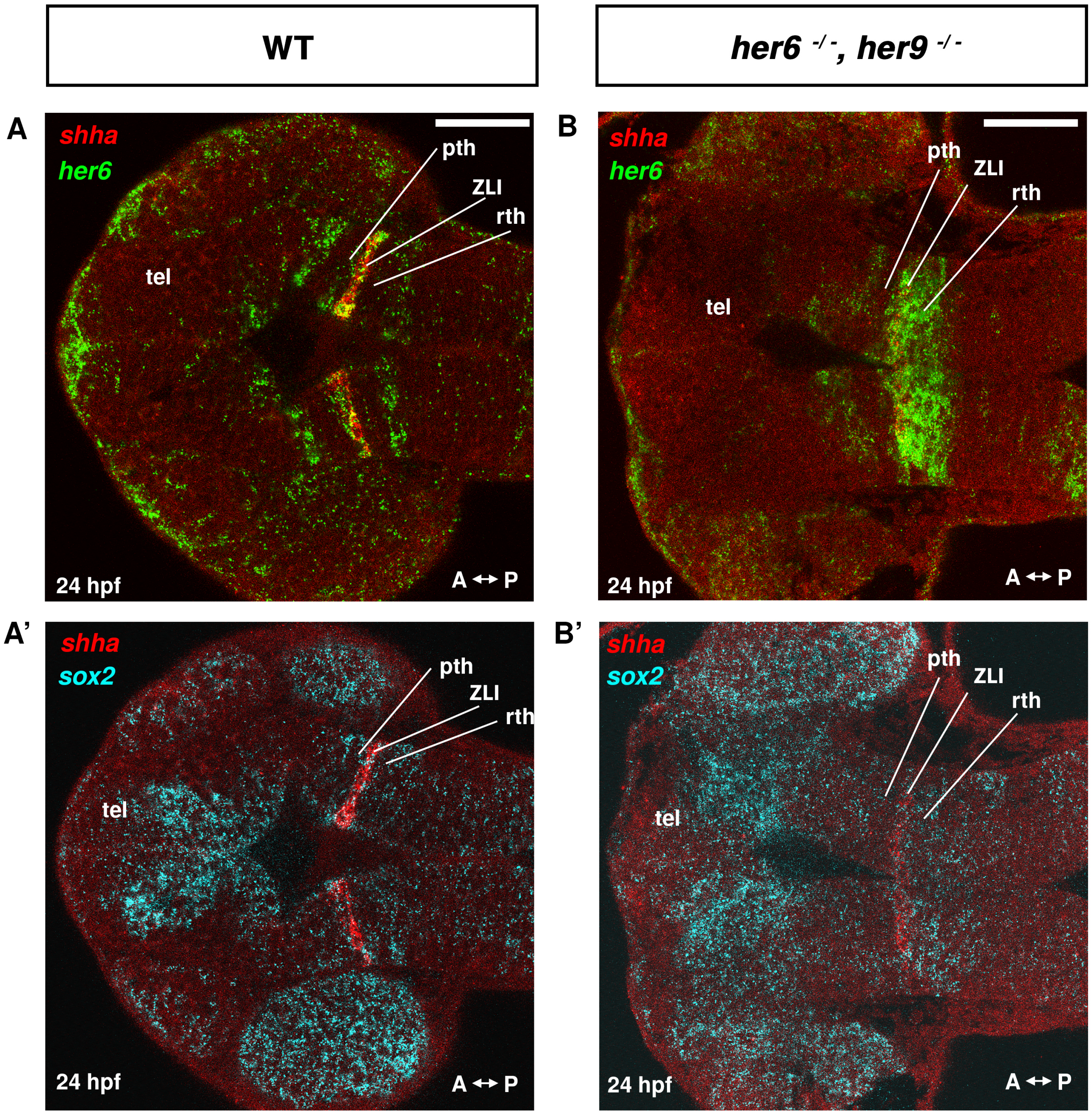

Fig. S11. Loss of *her6* and *her9* activity causes failure of *shha* expression to expand dorsally and establish the full ZLI and TPZ NSCs.

(A-B) Triple FISH for expression of *shha*, *her6* and *sox2* in the 24 hpf TPZ of WT and *her6,her9* double mutant embryos. Single image planes of dorsal view confocal stacks (A, A' and B, B' show different channels in same planes, respectively). B shows the strong upregulation of *her6* transcripts (green) in the absence of negative auto- and cross-regulation in *her6,her9* double mutant embryos. Numbers of embryos analyzed: A, n=2; B, n=2. Scale bar is 50 µm.

Development • Supplementary information

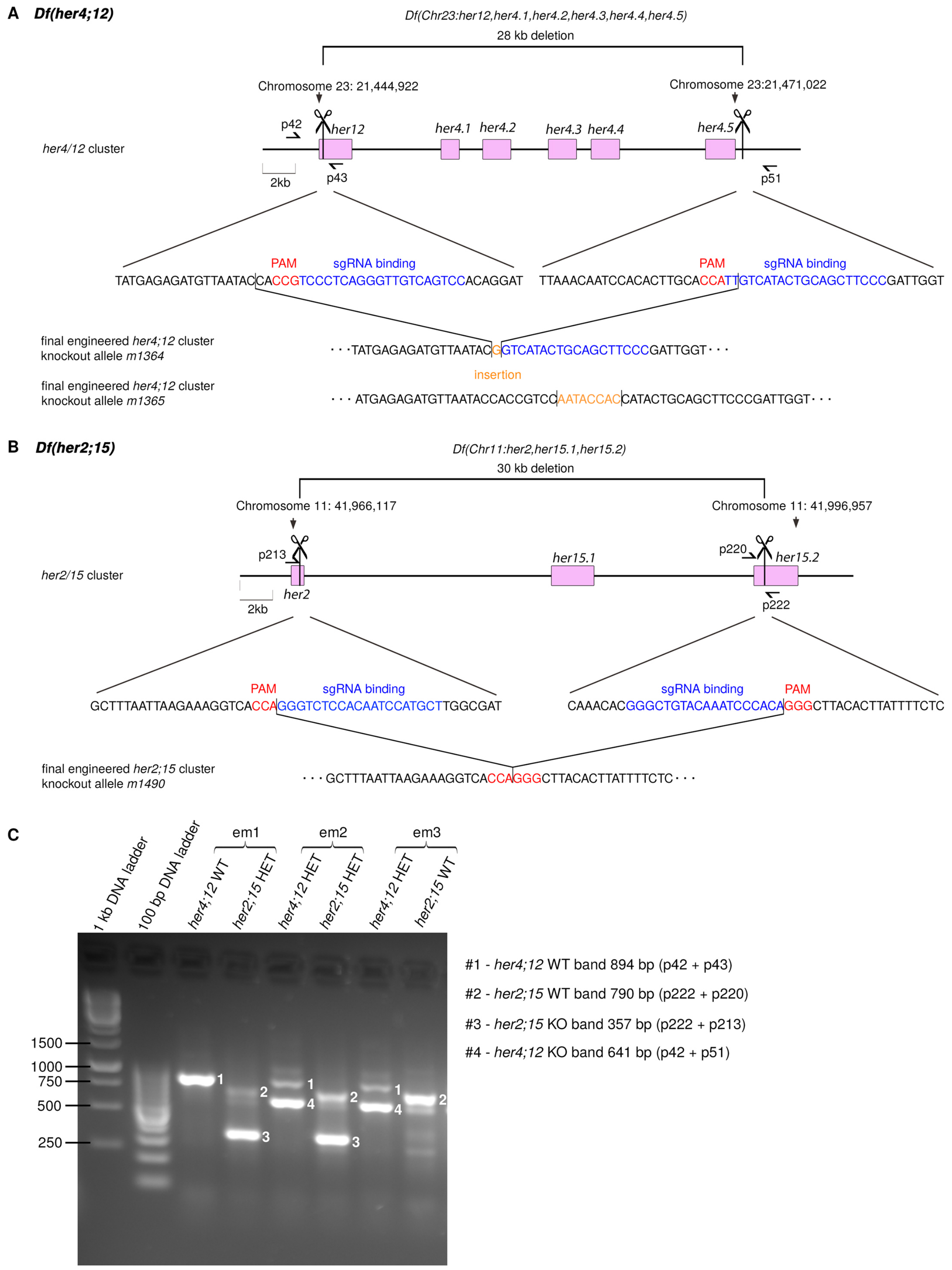

Fig. S12. Strategy for generating *Df(her4;12)* and *Df(her2;15)*.

(A, B) Knockout strategy to generate *Df(her4;12)* and *Df(her2;15)* using the CRISPR/Cas9 system. Scissors indicate where the sgRNAs bind and square brackets indicate the deletion. Half-arrows indicate binding sites for primers which were used in genotyping PCRs (tagged primer names). In the base sequence, PAM sequences are indicated in red, sgRNA binding sites are indicated in blue,

Development • Supplementary information

and insertions are indicated in orange. Three alleles (*Df(her4;12)m1364, Df(her4;12)m1365* and *Df(her2;15)m1490*) were used in this study, and the exact sequence mutation of the final cluster deficiencies are shown. For *her2* only the 3'UTR remains in the genome, and for *her15.2* only a part of the 5'UTR is not deleted. **(C)** Example gel picture of PCR products amplified for WT and *Df(her4;12)* and *Df(her2;15)* deficiency alleles, primers as indicated in (A, B). Primer p42 and p43 amplify a *her4;12* WT genomic fragment, while p42 + p51 amplify a *Df(her4;12)m1365* allele specific genomic fragment linking the deficiency ends. Primer p222 + p220 amplify the indicated genomic sequence of the *her2;her15* WT allele, while p222 and p213 amplify the *Df(her2;15)m1490* specific genomic fragment linking the deficiency ends. p43 and p220 binding sites are deleted in *Df(her4;12)* or *Df(her2;15)* alleles, respectively. The numbers 1 to 4 shown in the gel picture next to gel bands mark the identity of the bands as indicated in the index #1 - #4 at the right. Three embryos (em1 to em3) with the indicated genotypes are shown as examples.

Development • Supplementary information

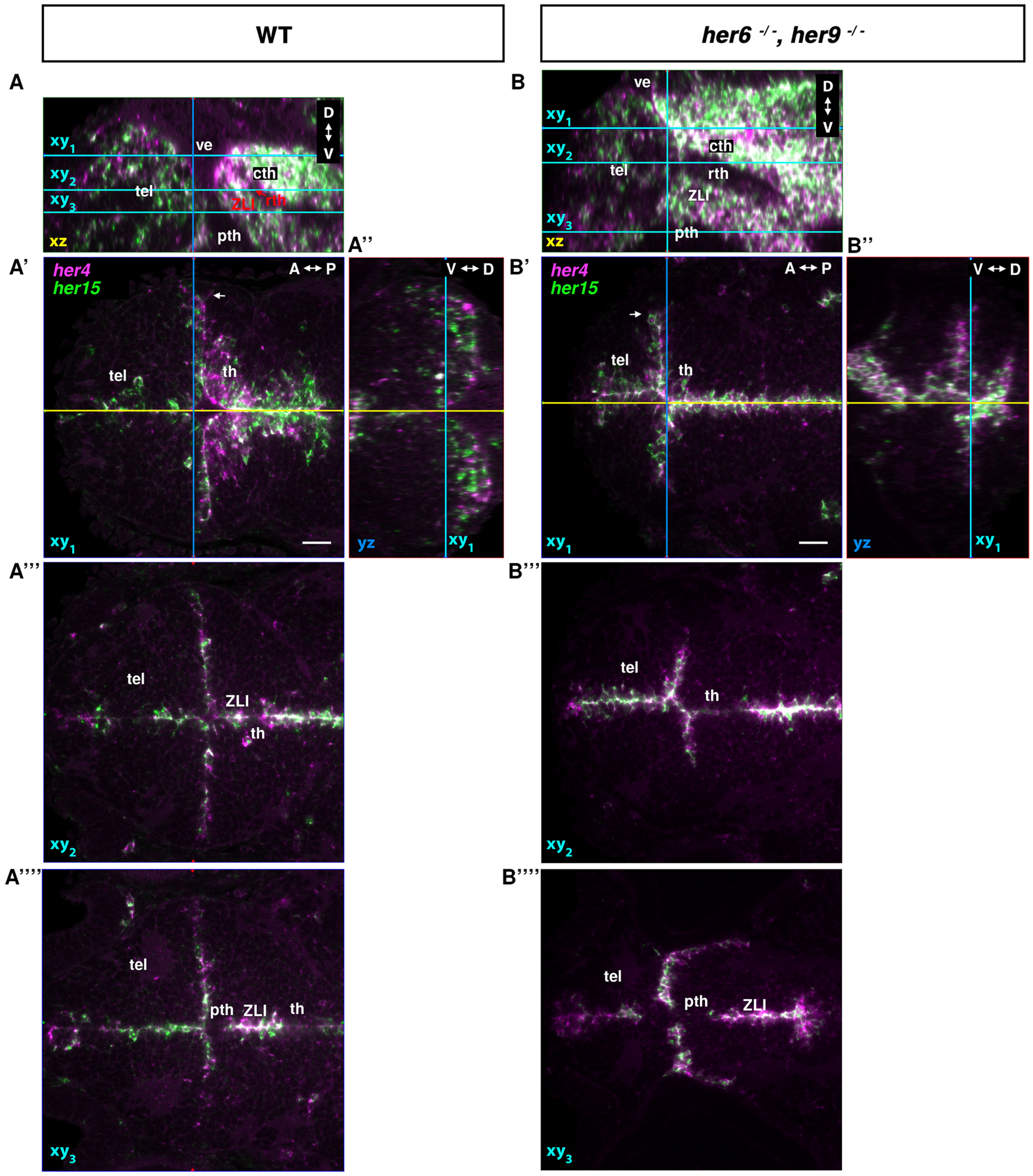

Fig. S13. Expression of *her4* and *her15* does not invade into the rostral thalamic and prethalamic *her6* expression domains in *her6, her9* double mutants.

(A-B'''') Confocal image stacks of FISH for *her4* and *her15* expression in 72 hpf WT **(A)** and *her6, her9* double mutant embryos (B). **(A, B)** Lateral view midline sagittal XZ plane from orthogonal reconstructions, cyan lines indicate the dorsal view horizontal confocal planes 1, 2 and 3 shown in A', A''', A'''', B', B''' and B'''', respectively**.** The yellow lines indicate the midline sagittal planes in A, B. The blue lines indicate the frontal plane in A'' and B''. **(A'' and B'')** Frontal view midline sagittal YZ plane from orthogonal reconstructions, cyan lines indicate the dorsal view horizontal confocal planes shown in A' and B', respectively. Scale bars 20 µm. Numbers of embryos analyzed: A, n=2; B, n=2.

Development • Supplementary information

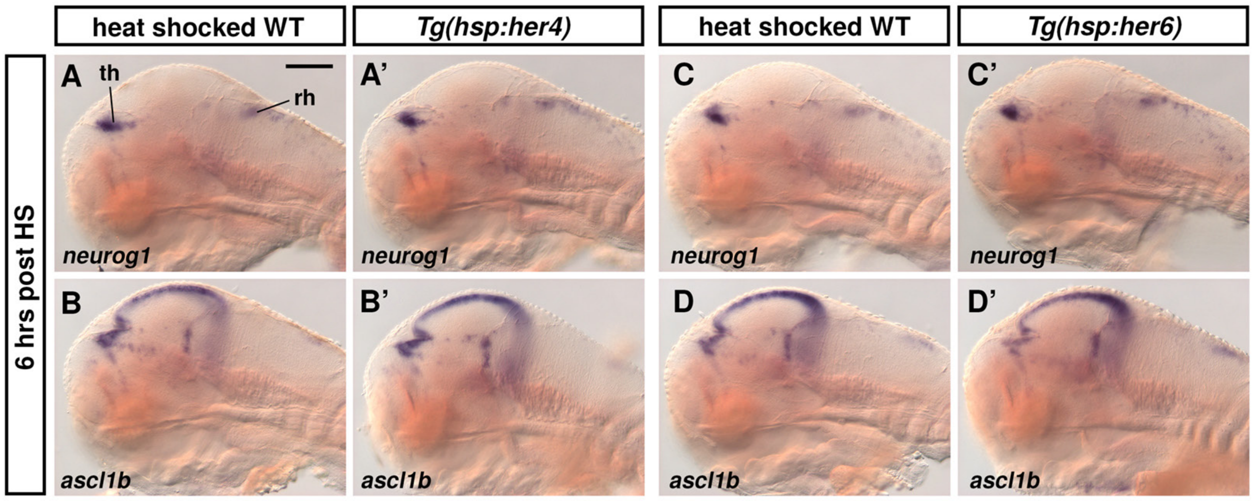

Fig. S14. Proneural gene expression is back to normal 6 hrs after heat shock overexpression of Her4 or Her6.

(A-D') WISH for expression of *neurog1* and *ascl1b* in larvae fixed 6 hrs after heat shock induced overexpression of Her4 or Her6, respectively. **(A', B')** *Tg(hsp:her4-FLAG)* was used to overexpress Her4. **(C', D')** *Tg(hsp:her6-FLAG)* was used to overexpress Her6. Heat shocked wild type siblings serve as control (A, B, C, D). All larvae shown in this panel were heat shocked at 70 hpf for 30 min and were fixed at 76 hpf (6 hrs after heat shock start). Three larvae per condition were imaged and the WISH staining patterns were consistent. th, thalamus proper; rh, rhombencephalon. Lateral views midsagittal optical sections; scale bar 100 µm in A for all panels.

Development • Supplementary information

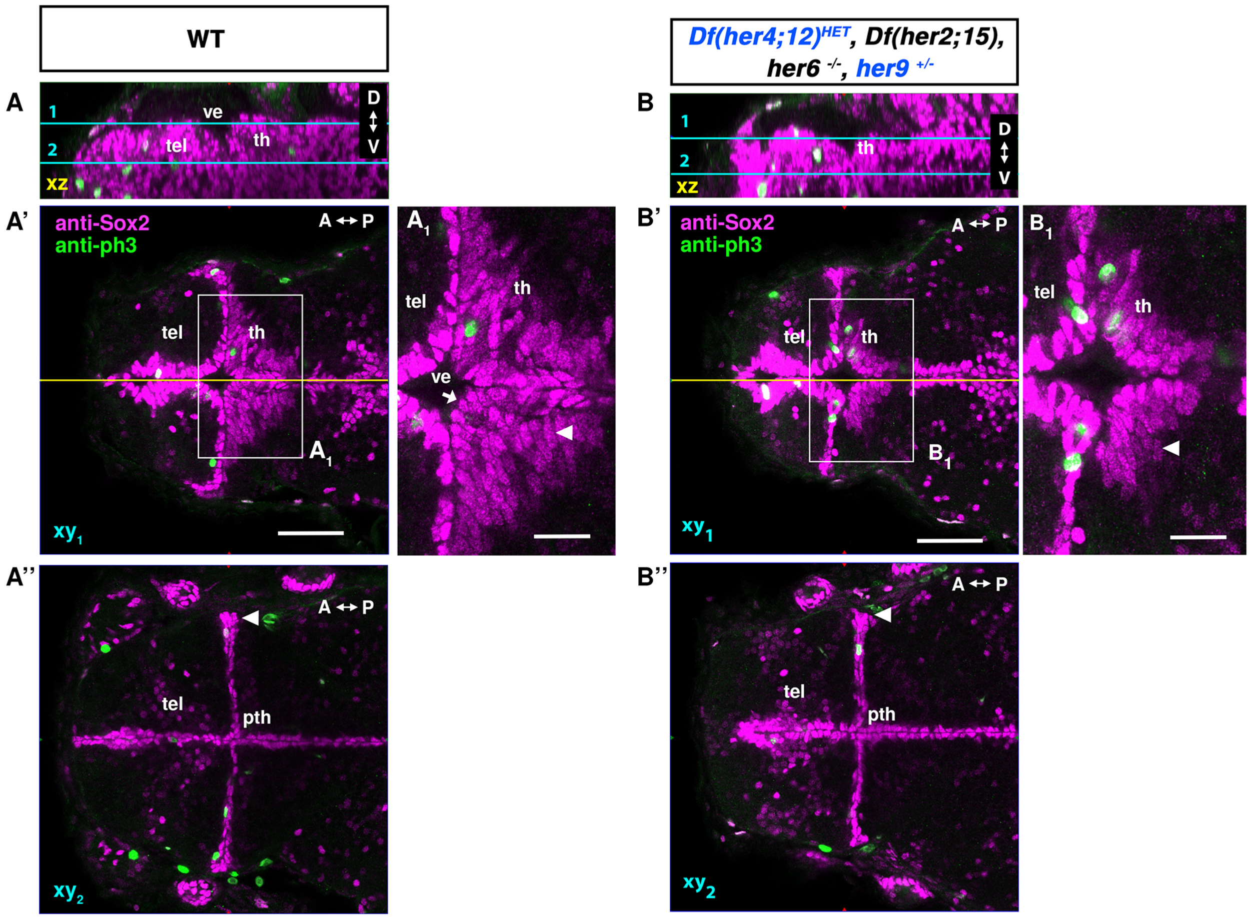

Fig. S15. Incomplete rescue *her*UDM mutant phenotype by single alleles of *her9* and *her4;12*.

(A-B'') Anti-Sox2 and anti-pH3 immunofluorescence in 72 hpf embryos, genotypes as indicated. **(A, B)** Lateral view midline sagittal XZ plane from orthogonal reconstructions, cyan lines indicate the dorsal view horizontal confocal planes 1 and 2 shown in A', B', A'', B'', respectively (A', B' dorsal part of the diencephalon with the thalamus proper; A'', B'' is located more ventrally including the prethalamus). Yellow lines in A', B' indicate the midline sagittal planes in A, B. A_1 and B_1 are magnifications of the boxed areas in A' and B'. Scale bars in A' and B' (for A-B'') 50 µm; in A_1 and B_1 20 µm. Number of embryos analyzed: A, n=1; B, n=1

Development • Supplementary information

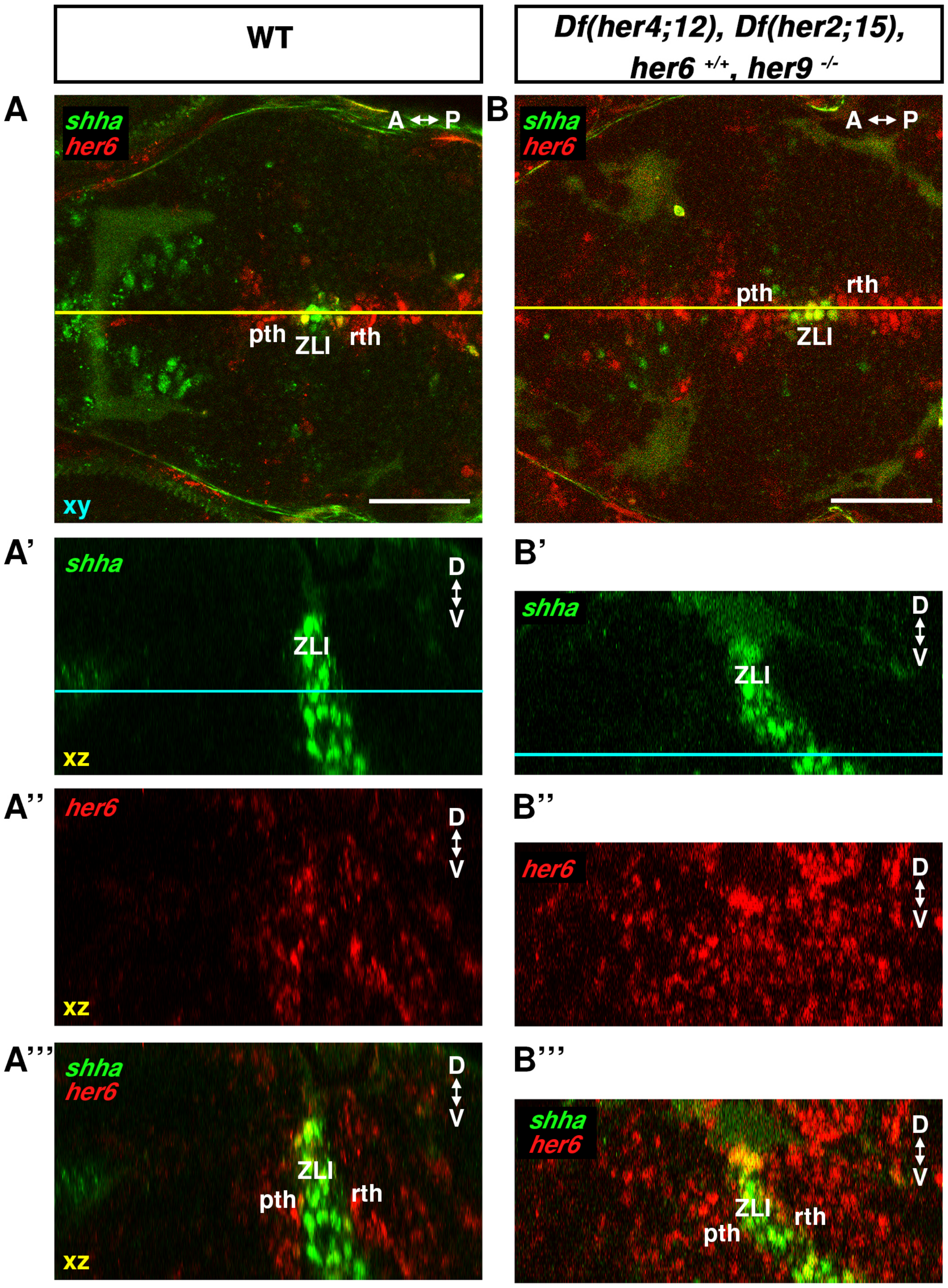

Fig. S16. Expansion of *her6* expression in the TPZ of *her*UDM mutant embryos.

(A-B''') Confocal image stacks of FISH for *her6* and *shha* expression in 72 hpf WT **(A)** and *Df(her4;12),Df(her2;15),her9* combined mutant embryos **(B)**. **(A, B)** Dorsal view single confocal image planes. The yellow lines indicate the midline sagittal planes in A'-B'''. Number of embryos analyzed n=2 for WT control, n=3 for *her*UDM with *her6*$^{+/+}$ rescue, and n=1 for *her*UDM *her6*$^{+/-}$ rescue. **(A'-B''')** Lateral view midline sagittal XZ plane from orthogonal reconstructions, cyan lines indicate the dorsal view horizontal confocal planes shown in A and B, respectively. Scale bars 50 µm.

Development • Supplementary information

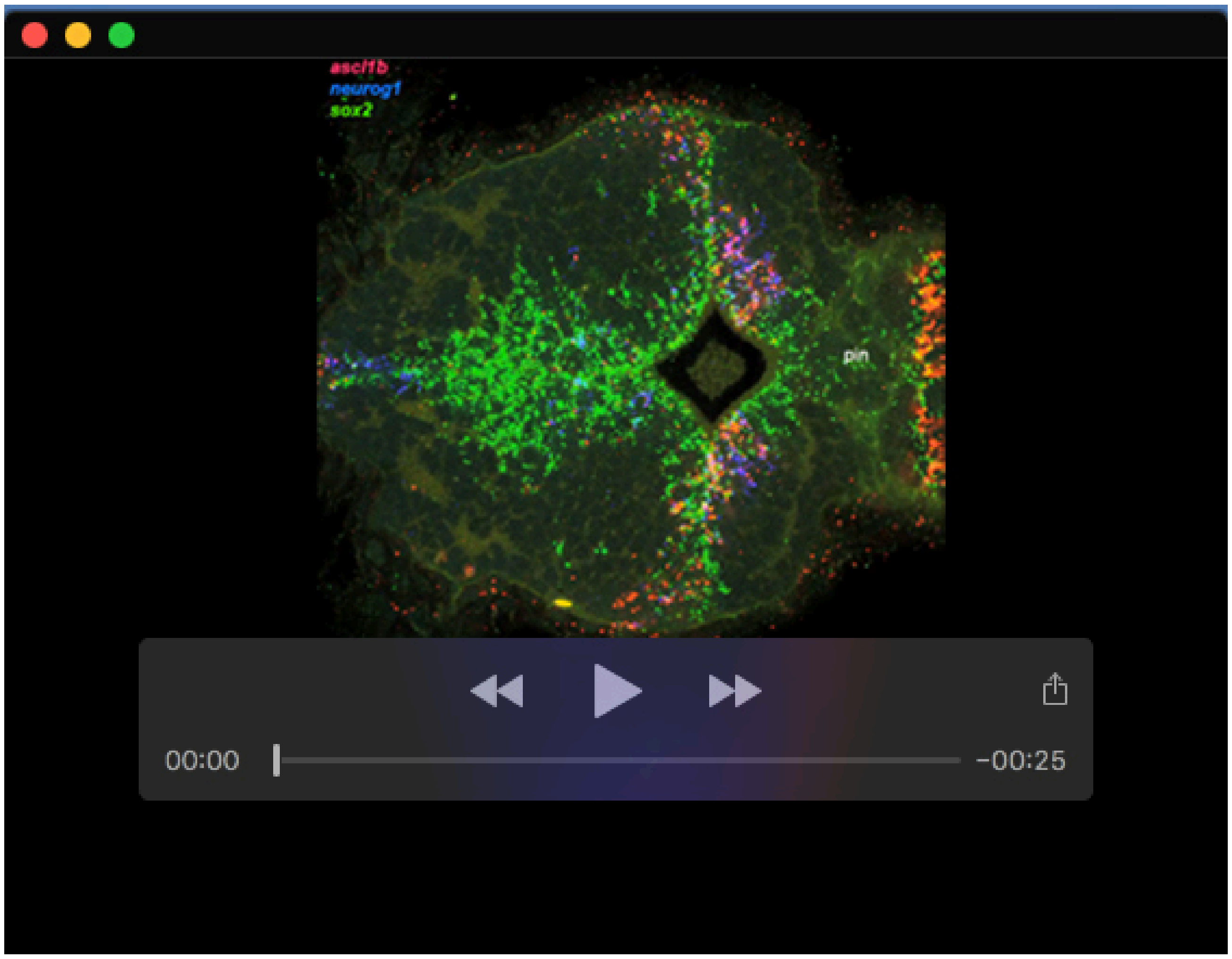

Movie 1. Analysis of NPC marker expression reveals *sox2*^{low} expressing cells as progenitors. Movie from stack of HCR expression analysis shown in Fig. 4C-C'' and Fig. S5 (see legends for details). The movie analyzes *ascl1b* (red), *neurog1* (blue) and *sox2* (green) expression in the thalamus of a 72 hpf WT larva. The movie starts with the dorsal most horizontal plane of the image stack and ends with the ventral most plane. Orientation: anterior to the left. pin, pineal gland; pth, prethalamus; th, thalamus proper.

Development • Supplementary information

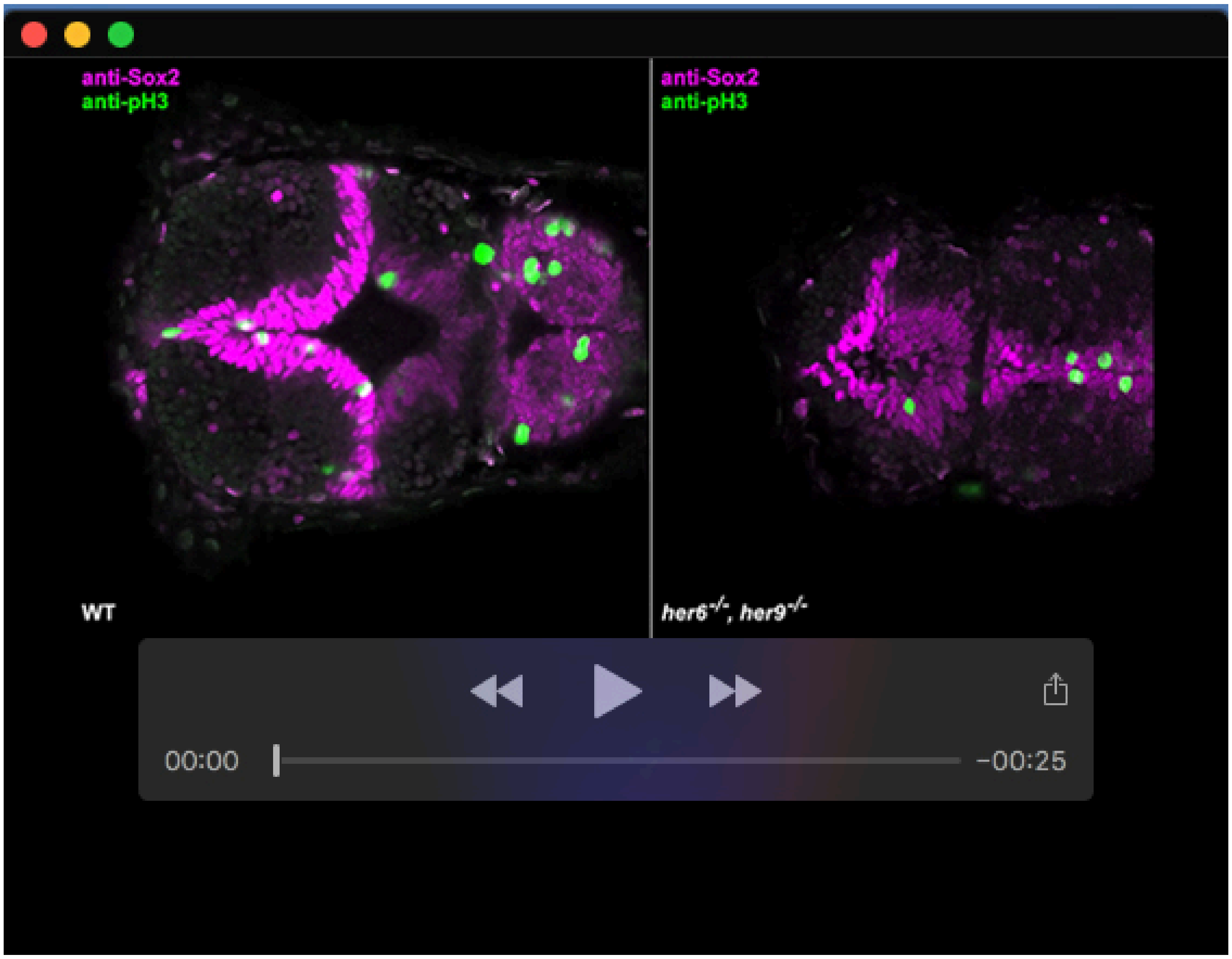

Movie 2. Combined movie of WT (left) and *her6, her9* double mutant larvae (right). Combined movie of larvae shown in Fig. 4A (WT; left half) and Fig. 4B (*her6*$^{-/-}$, *her9*$^{-/-}$double mutant; right half). The movie shows an anti-Sox2 (magenta) and anti-pH3 (green) double immunostaining horizontal image plane confocal stack of 72 hpf WT larvae recorded from a dorsal view. The movie starts with the dorsal-most plane of the image stack and ends with the ventral- most plane. Black slides were inserted when one stack had less z-planes than the other stack (here the ventral slides of the WT part). Since mutant and WT have different phenotypic appearances and also differ in size, the planes were aligned according to the thalamic Sox2 expression. This means that more ventral regions might be shifted. Orientation: anterior to the left.

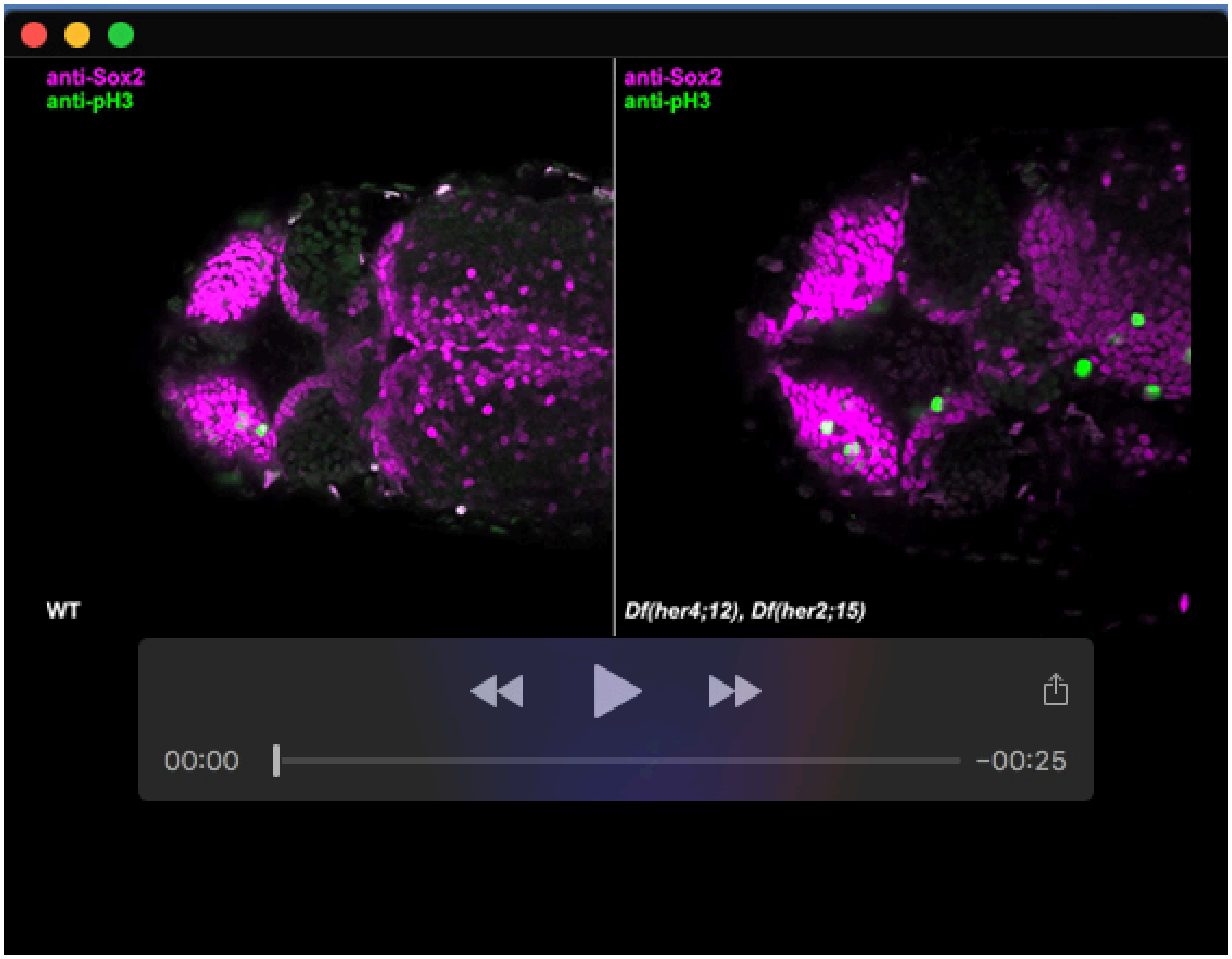

Movie 3. Combined movie of WT (left) and *Df(her4;12), Df(her2;15)* double mutant (right). Combined movie of larvae shown in Fig. 6A (WT; left half) and Fig. 6B [*Df(her4;12), Df(her2;15)* double mutant; right half]. The movie shows an anti-Sox2 (magenta) and anti-pH3 (green) double immunostaining confocal horizontal image stack of 72 hpf WT larvae recorded from a dorsal view. The movie starts with the dorsal-most plane of the image stack and ends with the ventral-most plane. Black slides were inserted when one stack had less z-planes than the other stack. Orientation: anterior to the left.

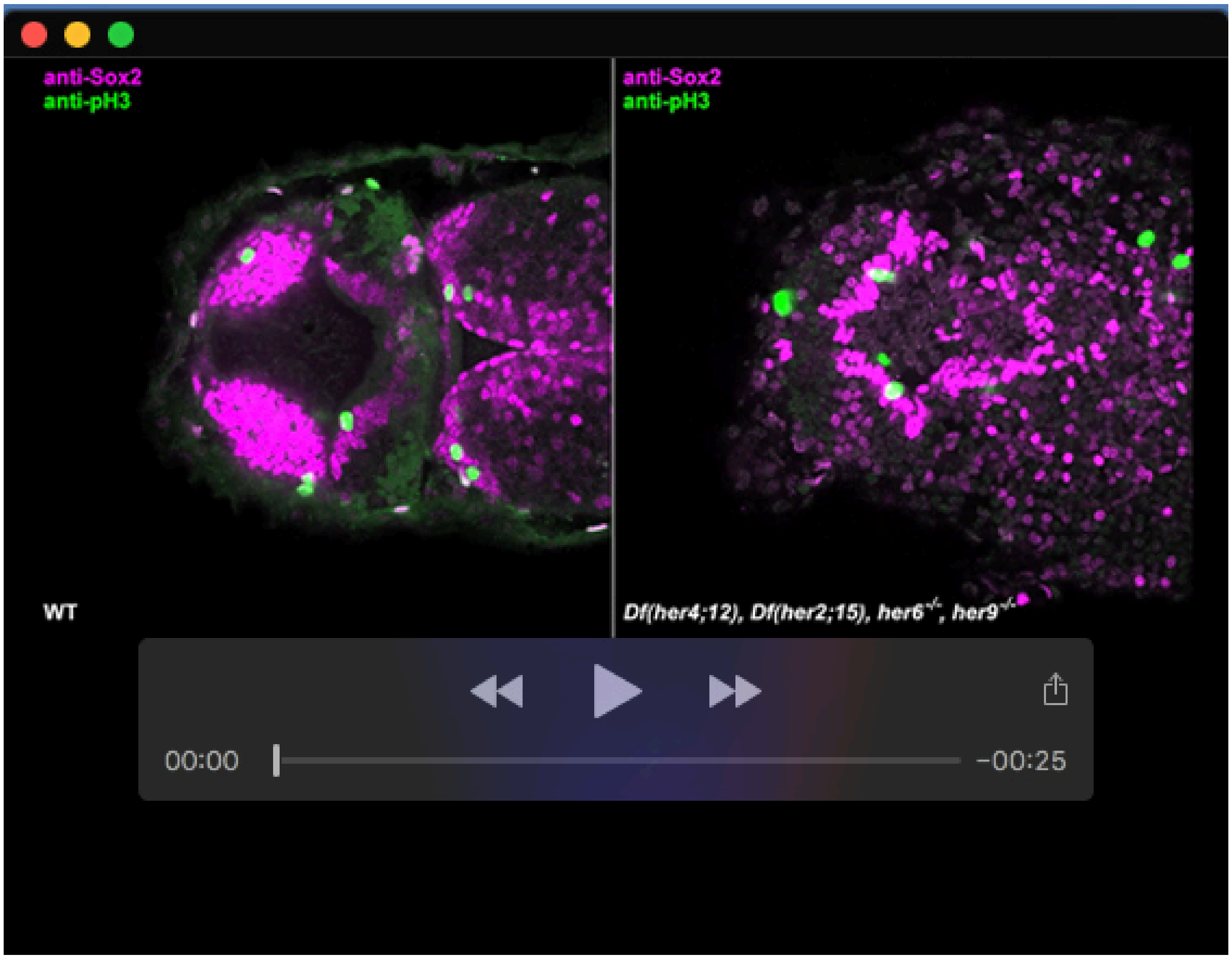

Movie 4. Combined movie of WT (left) and *Df(her4;12), Df(her2;15), her6$^{-/-}$, her9$^{-/-}$* mutant (*her*UDM, right). Combined movie of larvae shown in Fig. 9C (WT; left half) and Fig. 9D [*Df(her4;12), Df(her2;15), her6$^{-/-}$, her9$^{-/-}$* combined mutant; right half]. The movie shows an anti- Sox2 (magenta) and anti-pH3 (green) immunostaining confocal horizontal image stack of 72 hpf WT larvae recorded from a dorsal view. The movie starts with the dorsal most plane of the image stack and ends with the ventral most plane. Since WT and *her*UDM have different phenotypic appearances and also differ in size, black slides were inserted at the beginning and end to align z-slides according to the thalamic Sox2 expression. This means that more ventral and dorsal regions might be shifted. Orientation: anterior to the left.

Development • Supplementary information

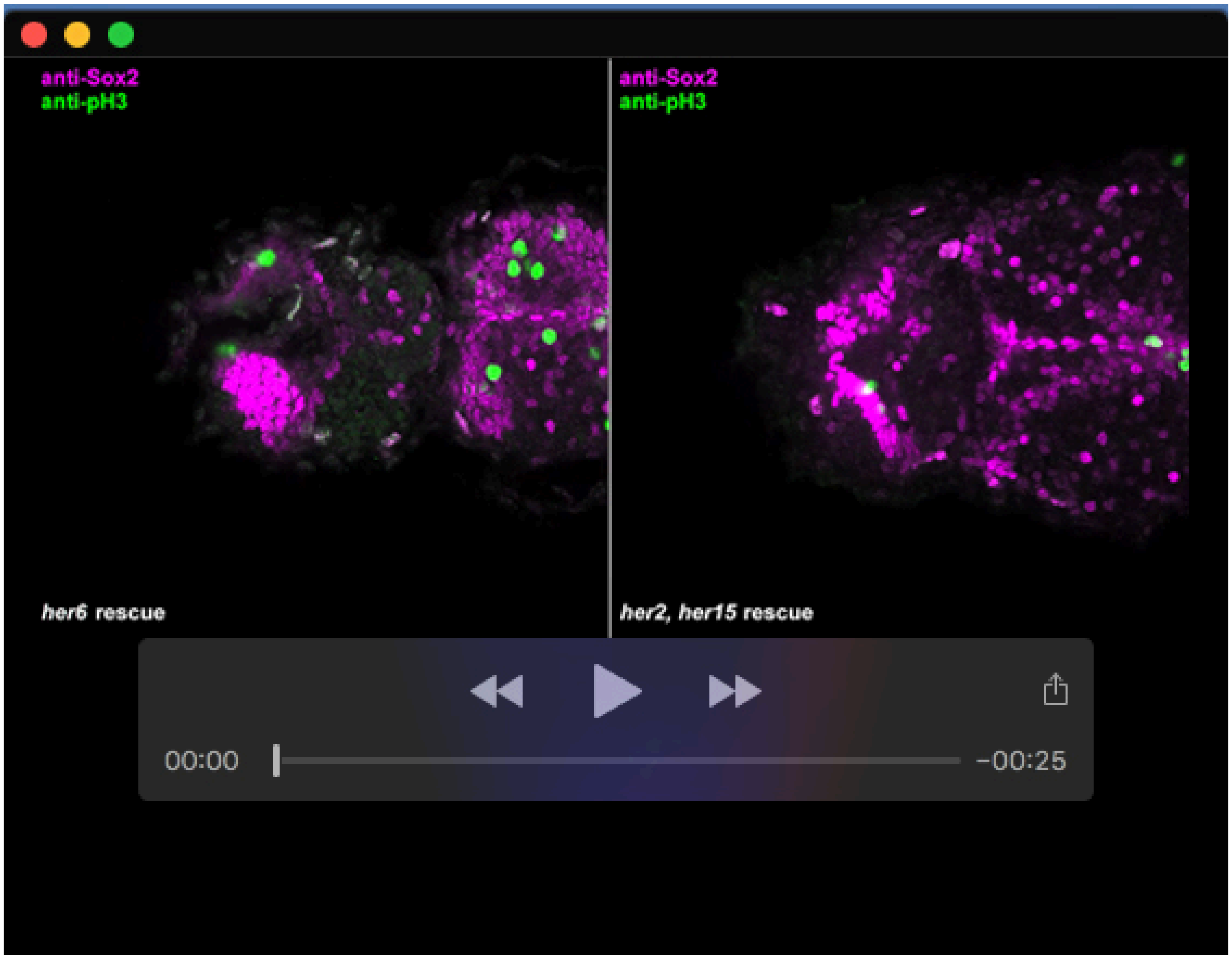

Movie 5. Combined movie of *her6* rescue (left) and *her2, her15* rescue (right) of *her*UDM mutants.

Combined movie of larvae shown in Fig. 9E ("*her6* rescue" of *her*UDM mutant; left half; genotype *Df(her4;12), Df(her2;15), her6*$^{+/-}$, *her9*$^{-/-}$ mutant) and Fig. 9F ("*her2, her15* rescue" of *her*UDM mutant; right half; genotype *Df(her4;12), Df(her2;15) heterozygous, her6*$^{-/-}$, *her9*$^{-/-}$ mutant). The movie shows an anti-Sox2 (magenta) and anti-pH3 (green) immunostaining confocal horizontal image stack of 72 hpf WT larvae recorded from a dorsal view. The movie starts with the dorsal most plane of the image stack and ends with the ventral most plane. Since both embryos have different phenotypic appearances and also differ in size, the planes were aligned according to the thalamic Sox2 expression. Orientation: anterior to the left.

Development • Supplementary information

Abbreviations used in Supplementary Figures

Abbreviations	Anatomical Term
A-P	anterior-posterior
cth	caudal thalamus
CeP	cerebellar plate
D-V	dorsal-ventral
E	epiphysis
Ha	habenula
hyp	hypothalamus
MHB	midbrain-hindbrain boundary
PTd	dorsal part of posterior tuberculum
ptec	pretectum
pth	prethalamus (ventral thalamus)
PTv	ventral part of posterior tuberculum
rh	rhombencephalon
rth	rostral thalamus
T	midbrain tegmentum
tec	tectum opticum
tel	telencephalon
th	thalamus proper (dorsal thalamus), subdivided into rostral thalamus (rth) and caudal thalamus (cth)
ve	ventricle
ZLI	zona limitans intrathalamica

Development • Supplementary information

Table S1. Compilation of information on zebrafish *hes/her* genes (Table modified from Chapouton et al., 2011, Table 1; References listed at end of table)

Gene	Notch regulation (in embryo)	Ensembl number	Embryonic expression	Mouse orthologue	Embryonic function	Expression in TPZ
her1	Positively regulated by Notch (Takke and Campos-Ortega,1999)	ENSDARG00000014722	Forming somites and presomitic mesoderm (Takke and Campos-Ortega,1999; Henry et al.,2002)	Hes7 (Davis and Turner, 2001)	Somitogenesis (Henry et al.,2002), inhibits neurogenesis	Not expressed in TPZ of larvae 1-5 dpf (Thisse et al., 2005) https://zfin.org/action/image/view/ZDB-IMAGE-051103-534
her2	?	ENSDARG00000038205	Proneural clusters (data not shown in Bae et al.,2005)	Hes5 (Ensembl database; Sieger et al., 2004)	?	in this study
her3	Independent of endogenous Notch at neural plate stages, repressed in overexpression conditions (Bae et al..2005; Hans et al..2004)	ENSDARG00000076857	Longitudinal progenitor pools of the presumptive spinal cord (Hans et al.,2004; Bae et al.,2005)	Hes3 (Davis and Turner,2001)	Inhibits neurogenesis (Hans et al.,2004; Bae et al.,2005)	Not expressed in TPZ of larvae 1-5 dpf (Thisse et al., 2005) https://zfin.org/action/image/view/ZDB-IMAGE-051107-264
her4.1	Positively regulated by Notch (Takke et al.,1999)	ENSDARG00000056729	Proneural clusters (Haddon et al.,1998; Takke et al.,1999)	Hes5 (Sieger et al.,2004)	Inhibits neurogenesis (Takke et al.,1999)	In this study
her5	Independent of endogenous Notch at neural plate stages, repressed in overexpression conditions (Geling et al.,2003)	ENSDARG00000008796	Progenitor pool of the midbrain–hindbrain boundary (MHB)	Hes7 (Gajewski and Voolstra,2002)	Inhibits neurogenesis (Geling et al.,2003)	Not expressed in TPZ of larvae 1-5 dpf (Thisse et al., 2005) http://zfin.org/ZDB-IMAGE-060130-195
her6	Positively regulated by Notch in the presomitic mesoderm (Pasini et al.,2004)	ENSDARG00000006514	Presomitic mesoderm (Pasini et al.,2004), later ZLI (Scholpp et al.,2006) and some proneural clusters (this paper)	Hes1 (Davis and Turner,2001)	Inhibits neurogenesis (Cunliffe,2004; Scholpp et al.,2009)	In this study
her7	?	ENSDARG00000017917	Presomitic mesoderm (Henry et al.,2002; Sieger et al.,2006)	Hes7 (Sieger et al.,2006)	Somitogenesis (Henry et al.,2002)	Not expressed in TPZ of larvae 1-5 dpf (Thisse et al., 2005) https://zfin.org/action/image/view/ZDB-IMAGE-030521-93
her8a	Independent of endogenous Notch at neural plate stages, activated in overexpression conditions (Webb et al., 2011)	ENSDARG00000016363	Proneural clusters and progenitor pools (Webb et al., 2011)	Hes6 (Ensembl database; Sieger et al.,2006)	Inhibits neurogenesis (Webb et al., 2011)	In this study

Development • Supplementary information

Gene	Notch regulation (in embryo)	Ensembl number	Embryonic expression	Mouse orthologue	Embryonic function	Expression in TPZ
her9	Independent of endogenous Notch at neural plate stages incl. in the floorplate, unaffected in overexpression conditions (Bae et al.,2005; Latimer et al.,2005)	ENSDARG00000056438	Eye field, longitudinal progenitor pools of the presumptive spinal cord, progenitor pool of the MHB (Bae et al.,2005)	Hes1 (Ensembl database, (Sieger et al.,2004))	Inhibits neurogenesis (Bae et al.,2005); promotes floor plate development (Latimer et al.,2005)	In this study
helt	?	ENSDARG00000056400	Presumptive tegmentum and tectal proliferation zones at 3 dpf (Chapouton et al., 2011)	*Heslike/Helt/Mgn* (Miyoshi et al.,2004; Guimera et al.,2006a; Nakatani et al.,2007)	Unknown in zebrafish; in mouse, modifies the proneural code expressed by neuronal progenitors (Guimera et al.,2006b; Nakatani et al., 2007)	Is expressed at 24 hpf in zli and pretectum (Chapouton et al., 2011)<br>is expressed in rostral thalamus at 48hpf (Peukert et al., 2011)
her11	Independent of endogenous Notch at neural plate stages, repressed in overexpression conditions	ENSDARG00000002707	Progenitor pool of the MHB; expressed in hindbrain (with her5) (Webb et al., 2011)	Hes7 (Gajewski and Voolstra,2002)	Somitogenesis (Sieger et al.,2006); inhibits neurogenesis (Ninkovic et al.,2005)	Not expressed in TPZ of larvae 1-5 dpf (Webb et al., 2011; Ninkovic et al., 2005)
her12	?	ENSDARG00000032963	Proneural clusters (Bae et al.,2005), presomitic mesoderm (Shankaran et al.,2007)	Hes5 (Shankaran et al.,2007)	Somitogenesis (Shankaran et al.,2007)	In this study
her13.1 *(clustered with her8.2)*	?	ENSDARG00000007097	Proneural clusters (Webb et al., 2011)	Hes6 (Sieger et al.,2006)	?	Not expressed in TPZ before 24 hpf (Zhou et al., 2012)
hes6 (was *her13.2*)	Independent of endogenous Notch at neural plate stages (Kawamura et al.,2005)	ENSDARG00000019335	Presomitic mesoderm (Shankaran et al.,2007), later nervous system and the retina (Chapouton et al., 2011)	Hes6 (Sieger et al.,2006)	Somitogenesis (Sieger et al.,2006), function in neural plate to date untested.	Early, not expressed in TPZ, after 24hpf broadly expressed in neural tissues (Thisse et al., 2005) https://zfin.org/action/image/view/ZDB-IMAGE-060216-396
her15 (was *hes5*)	Positively regulated by Notch (Bae et al.,2005)	ENSDARG00000054560	Proneural clusters (Bae et al.,2005)	Hes5 (Shankaran et al.,2007)	Somitogenesis (Shankaran et al.,2007), function in neural plate to date untested	In this study

Development • Supplementary information

References in Table S1:

Bae, Y. K., Shimizu, T., & Hibi, M. (2005). Patterning of proneuronal and inter-proneuronal domains by hairy-and enhancer of split-related genes in zebrafish neuroectoderm. *Development,* 132, 1375–1385.

Chapouton, P., Webb, K. J., Stigloher, C., Alunni, A., Adolf, B., Hesl, B., Topp, S., Kremmer, E. & Bally-Cuif, L. (2011). Expression of hairy/enhancer of split genes in neural progenitors and neurogenesis domains of the adult zebrafish brain. *Journal of Comparative Neurology*, 519, 1748-1769.

Cunliffe, V. T. (2004). Histone deacetylase 1 is required to repress Notch target gene expression during zebrafish neurogenesis and to maintain the production of motoneurones in response to hedgehog signalling. *Development,* 131, 2983–2995.

Davis, R. L., & Turner, D. L. (2001). Vertebrate hairy and Enhancer of split related proteins: transcriptional repressors regulating cellular differentiation and embryonic patterning. *Oncogene*, *20*, 8342-8357.

Gajewski, M., & Voolstra, C. (2002). Comparative analysis of somitogenesis related genes of the hairy/Enhancer of split class in Fugu and zebrafish. *BMC genomics*, *3*, 1-15.

Geling, A., Itoh, M., Tallafuß, A., Chapouton, P., Tannhäuser, B., Kuwada, J. Y., Chitnis, A.B., & Bally-Cuif, L. (2003). bHLH transcription factor Her5 links patterning to regional inhibition of neurogenesis at the midbrain-hindbrain boundary. *Development*, 130, 1591–1604.

Guimera, J., Weisenhorn, D. V., Echevarría, D., Martínez, S., & Wurst, W. (2006). Molecular characterization, structure and developmental expression of Megane bHLH factor. *Gene*, *377*, 65-76.

Haddon, C., Smithers, L., Schneider-Maunoury, S., Coche, T., Henrique, D., & Lewis, J. (1998). Multiple delta genes and lateral inhibition in zebrafish primary neurogenesis. *Development*, *125*, 359-370.

Hans, S., Scheer, N., Riedl, I., Weizsäcker, E. V., Blader, P., & Campos-Ortega, J. A. (2004). her3, a zebrafish member of the hairy-E (spl) family, is repressed by Notch signalling. *Development*, 131, 2957–2969.

Henry, C. A., Urban, M. K., Dill, K. K., Merlie, J. P., Page, M. F., Kimmel, C. B., & Amacher, S. L. (2002). Two linked hairy/Enhancer of split-related zebrafish genes, her1 and her7, function together to refine alternating somite boundaries. *Development,* 129, 3693–3704.

Kawamura, A., Koshida, S., Hijikata, H., Sakaguchi, T., Kondoh, H., & Takada, S. (2005). Zebrafish hairy/enhancer of split protein links FGF signaling to cyclic gene expression in the periodic segmentation of somites. *Genes & development*, *19*, 1156-1161.

Latimer, A. J., Shin, J. and Appel, B. (2005). her9 promotes floor plate development in zebrafish. *Dev Dyn,* 232, 1098-1104.

Miyoshi, G., Bessho, Y., Yamada, S., & Kageyama, R. (2004). Identification of a novel basic helix-loop-helix gene, Heslike, and its role in GABAergic neurogenesis. *Journal of Neuroscience*, *24*, 3672-3682.

Nakatani, T., Minaki, Y., Kumai, M., & Ono, Y. (2007). Helt determines GABAergic over glutamatergic neuronal fate by repressing Ngn genes in the developing mesencephalon. *Development,* 134, 2783–2793.

Ninkovic, J., Tallafuss, A., Leucht, C., Topczewski, J., Tannhäuser, B., Solnica-Krezel, L., & Bally-Cuif, L. (2005). Inhibition of neurogenesis at the zebrafish midbrain-hindbrain boundary by the combined and dose-dependent activity of a new hairy/E (spl) gene pair. *Development*, 132, 75–88.

Pasini, A., Jiang, Y. J., & Wilkinson, D. G. (2004). Two zebrafish Notch-dependent hairy/Enhancer-of-split-related genes, her6 and her4, are required to maintain the coordination of cyclic gene expression in the presomitic mesoderm. *Development,* 131, 1529–1541.

Peukert, D., Weber, S., Lumsden, A., & Scholpp, S. (2011). Lhx2 and Lhx9 determine neuronal differentiation and compartition in the caudal forebrain by regulating Wnt signaling. *PLoS biology*, *9*, e1001218.

Scholpp, S., Wolf, O., Brand, M. and Lumsden, A. (2006). Hedgehog signalling from the zona limitans intrathalamica orchestrates patterning of the zebrafish diencephalon. *Development,* 133, 855-864.

Shankaran, S. S., Sieger, D., Schroter, C., Czepe, C., Pauly, M. C., Laplante, M. A., Becker, T. S., Oates, A. C. and Gajewski, M. (2007). Completing the set of h/E(spl) cyclic gene sin zebrafish: her12 and her15 reveal novel modes of expression and contribute to the segmentation clock. *Dev Biol,* 304, 615-632.

Sieger, D., Ackermann, B., Winkler, C., Tautz, D. and Gajewski, M. (2006). her1 and her13.2 are jointly required for somitic border specification along the entire axis of the fish embryo. *Dev Biol,* 293, 242-251.

Sieger, D., Tautz, D. and Gajewski, M. (2004). her11 is involved in the somitogenesis clock in zebrafish. *Dev Genes Evol,* 214, 393-406.

Stigloher, C., Chapouton, P., Adolf, B. and Bally-Cuif, L. (2008). Identification of neural progenitor pools by E(Spl) factors in the embryonic and adult brain. *Brain Res Bull*, 75, 266-273.

Takke, C., & Campos-Ortega, J. A. (1999). her1, a zebrafish pair-rule like gene, acts downstream of notch signalling to control somite development. *Development*, *126*, 3005-3014.

Takke, C., Dornseifer, P., v Weizsacker, E., & Campos-Ortega, J. A. (1999). her4, a zebrafish homologue of the Drosophila neurogenic gene E (spl), is a target of NOTCH signalling. *Development*, *126*, 1811-1821.

Webb, K. J., Coolen, M., Gloeckner, C. J., Stigloher, C., Bahn, B., Topp, S., Ueffing, M. and Bally-Cuif, L. (2011). The Enhancer of split transcription factor Her8a is a novel dimerisation partner for Her3 that controls anterior hindbrain neurogenesis in zebrafish. *BMC Dev Biol,* 11, 27.

Zhou, M., Yan, J., Ma, Z., Zhou, Y., Abbood, N. N., Liu, J., Su, L., Jia, H. and Guo, A. Y. (2012). Comparative and evolutionary analysis of the HES/HEY gene family reveal exon/intron loss and teleost specific duplication events. *PLoS One,* 7, e40649.

Development • Supplementary information

Table S2. Primer and sgRNA templates

Primers are given with a short description, a short ID and the primer sequence. Oligonucleotide Sequences for the transcription of specific sgRNAs include: The T7 promoter (left) shown in lower case letters, the target specific region of the sgRNA (middle) in capital letters, and the annealing site for the constant oligonucleotide (right, lower case).

Description of primer	Short ID	sequence
Df(her2;15)		
sgRNA for *Df(her2;15)* at *her2* end	p211 - Oligo38	5′- taatacgactcactataGGCATGGATTGTGGAGACCCgttttagagctagaaatagcaag -3′
sgRNA for *Df(her2;15)* at *her15.2* end	p219 - Oligo43	5′- taatacgactcactataGGGCTGTACAAATCCCACAgttttagagctagaaatagcaag -3′
Df(her2;15) control primer F at *her2* end	p213F	5′- AACACCTCTGCAGGCTACAC -3′
Df(her2;15) control primer R at *her15.2* end	p222R	5′- CTTGCATGCAGTTCAATCTCACA -3′
Df(her2;15) control primer F at *her15.2* end	p220F	5′- GGATCCTGCTGCTGGAACTC -3′
Df(her4;12)		
sgRNA for *Df(her4;12)* at *her2* end	p39 - Oligo12	5′- taatacgactcactataGGACTGACAACCCTGAGGGAgttttagagctagaaatagcaag -3′
sgRNA for *Df(her4;12)* at *her4.5* end	p47 - Oligo16	5′- taatacgactcactataGGGAAGCTGCAGTATGACAAgttttagagctagaaatagcaag -3′
Df(her4;12) control primer F at *her12* end	p42F	5′- TATTGTACTTCTTGCACATTTTGCG -3′
Df(her4;12) control primer R at *her12* end	p43R	5′- GAAAAGATGCGTCGGGATCG -3′
Df(her4;12) control primer R at *her15.2* end	p51R	5′- CTCATGGCGAAGGATTGTCG -3′
her9 knockout		
sgRNA for *her9* knockout	p186 - Oligo26	5′-taatacgactcactataGGACTTCTTAACCACCTGTCgttttagagctagaa-3′
sgRNA for *her9* knockout	p187 - Oligo27	5′-taatacgactcactataGGGTATGAGATCCACTGGCAgttttagagctagaa-3′
her9 m1368 knockout control PCR F	p197F	5′-CCGGACTCAACTTTGGTGTTTG-3′
her9 m1368 knockout control PCR R	p198R	5′-GATGGGTAACGTTGAAGGAAGC-3′

Development • Supplementary information

Description of primer	Short ID	sequence
her6 knockout		
sgRNA for *her6* knockout	p3-Oligo04	5′-taatacgactcactataGCGAGAATCAACGAAAGCTTgttttagagctagaaatagcaag-3′
sgRNA for *her6* knockout	p4-Oligo05	5′-taatacgactcactataCGGTACTTCCCAAGAACGGTgttttagagctagaaatagcaag-3′
her6 m1358 knockout control PCR F	p5F	5′-TCAGCGTACTTGACAGCGTT-3′
her6 m1358 knockout control PCR R	p10R	5′-TCACATGTGGACAGGAACCG-3′

qPCR primer		
pPCR *her6* F	p372F	5′- TCCAAACGGCCCTGTTATTCC-3′
pPCR *her6* R	p373R	5′- AACGGAGTCTGACGTGACGG-3′
pPCR *her9* F	p376F	5′- CAACCCAGCGTTTGCTTCTG-3′
pPCR *her9* R	p377R	5′- CTGACACCAACGGGACTGAC-3′

Development • Supplementary information

Description of primer	Short ID	sequence
For cloning of in situ hybridization probes		
ISHprobe recognising *her4.1 ,4.2, 4.3, 4.4, 4.5* F	159F	5′- CTGAGAAAGCCCATGGTGGA-3′
ISHprobe recognising *her4.1 ,4.2, 4.3, 4.4, 4.5* R	160R	5′- CTACCAGGGTCTCCAGATGTGA-3′
ISHprobe *her6* F	p147F	5′- GCTGCCCTAAACACAGATCCC-3′
ISHprobe *her6* R	p148R	5′- TCATTTCAAATGTCATTTATTTGTCTTCCAAAAAG-3′
ISHprobe *her9* F	p149F	5′- CAGCCTTGTCAGCTGACACAA-3′
ISHprobe *her9* R	p150R	5′- CGTTATTTTGATTTATTCAGCATCAAACATAAACAC-3′
ISHprobe *her15.1, her15.2* F	p157F	5′- CAAGTCCATGCTGGAGAAAGAGT-3′
ISHprobe *her15.1, her15.2* R	p158R	5′- CATCTTTCATATAAAGAAACACCAATAAAGCA-3′
ISHprobe *her2* F	p154F	5′- CTGAGGAAACCGGTGGTGG-3′
ISHprobe *her2* R	p155R	5′- TGGTTTCAACTCAACATGAGATACTTAAAC-3′
ISHprobe *her12* F	p152F	5′- CTGAGGAAGCCGATAGTTGAAAAGA-3′
ISHprobe *her12* R	p153R	5′- CTAACAACAAACAAACAATAACACATCATTGG-3′
ISHprobe *her8a* F	p322F	5′- CACTGCTTGGAAGCAAATGA-3′
ISHprobe *her8a* R	p327R	5′- agaaaaatCAGCTTCAATATGAGGTACTAGTC-3′
ISHprobe *her8.2* F	p260F	5′- GAGCCCGACAGAGATACAGC-3′
ISHprobe *her8.2* R	p259R	5′- CAACACAGTATTAGTAGCCAAAGGG-3′

primer for identification of *Tg(UAS:NICD)kca3Tg* zebrafish		
NICD F		5′-CATCGCGTCTCAGCCTCAC-3′
NICD R		5′-CGGAATCGTTTATTGGTGTCG-3′

Development • Supplementary information

Table S3. HCR Oligonucleotides

Oligonucleotide sequences for *olig2, her4, her9*, and *her15* HCR. Upper-case letters indicate gene specific sequence. Spacer 1 and Spacer 2 (aa or ta) are given in lower-case letters adjacent to the gene specific sequences. Initiator 1 and Initiator 2 are given in lower-case letters next to the spacer sequences.

Description	Sequence
**Oligonucleotides for *olig2* HCR probe**	
HCR_Dan_olig2_B1_S1-1	gaggagggcagcaaacggaaCACTCGTGCAGTGTTTGTGTTTGGC
HCR_Dan_olig2_B1_S1-2	GAATTGACTTGTAAGGCTATTCCAGtagaagagtcttcctttacg
HCR_Dan_olig2_B1_S2-1	gaggagggcagcaaacggaaTCCATGGCGTTCAGTGCGCTCTCAG
HCR_Dan_olig2_B1_S2-2	CTGCTGGACACTCGGCTCGTGTCAGtagaagagtcttcctttacg
HCR_Dan_olig2_B1_S3-1	gaggagggcagcaaacggaaCCTCTTAACTCCGGTGGAGAATCGC
HCR_Dan_olig2_B1_S3-2	GCGCTGGAGATGCCCCGCAGATCTGtagaagagtcttcctttacg
HCR_Dan_olig2_B1_S4-1	gaggagggcagcaaacggaaCTCATTCTCGGAGAGAAGTTTACGG
HCR_Dan_olig2_B1_S4-2	GTTGATCTTTAGGCGCATGCTCTGCtagaagagtcttcctttacg
HCR_Dan_olig2_B1_S5-1	gaggagggcagcaaacggaaAGTTGCGCGCGAGCAGCAGAGTGGC
HCR_Dan_olig2_B1_S5-2	CCAGCGAGTTGCTCAGCATAAGGATtagaagagtcttcctttacg
HCR_Dan_olig2_B1_S6-1	gaggagggcagcaaacggaaGACGGCTGAAAGCCCCGTCCCGGAA
HCR_Dan_olig2_B1_S6-2	GGCTTTGAGGAGTCCGTGATGCGGCtagaagagtcttcctttacg
HCR_Dan_olig2_B1_S7-1	gaggagggcagcaaacggaaACGGCGCTCATACCGGGAACGTGAG
HCR_Dan_olig2_B1_S7-2	GAGTCACTGGTCAGCCGTGGCATGCtagaagagtcttcctttacg
HCR_Dan_olig2_B1_S8-1	gaggagggcagcaaacggaaCTAAGGAAGGTTTGCCATTTCCAAC
HCR_Dan_olig2_B1_S8-2	TTGTTCTTTCAGTCCTATAGTCGAGtagaagagtcttcctttacg
HCR_Dan_olig2_B1_S9-1	gaggagggcagcaaacggaaAAGAGCGCTTGGCAAATGAATTCTT
HCR_Dan_olig2_B1_S9-2	GCAGGAATGAACAGGTGCTTCTTTAtagaagagtcttcctttacg
HCR_Dan_olig2_B1_S10-1	gaggagggcagcaaacggaaCGGATAAGCTTGTGCAGAAAGAAGA
HCR_Dan_olig2_B1_S10-2	GTTGATCATTCTTCGCATCGGTAATtagaagagtcttcctttacg

Description	Sequence
**Oligonucleotides for *her4* HCR probe**	
HCR_Dan_her4_B1_S1-1	gaggagggcagcaaacggaaGAAATCAAGCGTCATCTCCAGGATA
HCR_Dan_her4_B1_S1-2	CGCACTGCTTTTCTGAGAGCGTCTCtagaagagtcttcctttacg
HCR_Dan_her4_B1_S2-1	gaggagggcagcaaacggaaTGCACACATCTGGAGCGTCCGTCAC
HCR_Dan_her4_B1_S2-2	CACTGAGACAGAAAGCTGACGGCCTtagaagagtcttcctttacg
HCR_Dan_her4_B1_S3-1	gaggagggcagcaaacggaaTCTTGTGTGGCTCTGCGTCTGCACT
HCR_Dan_her4_B1_S3-2	CTGCATGTGCAGGAAGAGCTTCATCtagaagagtcttcctttacg
HCR_Dan_her4_B1_S4-1	gaggagggcagcaaacggaaTCCACACGTGTGTGCTGGTCTGCAG
HCR_Dan_her4_B1_S4-2	GCGTGTGTTTCAGTGGTCTGAGGATtagaagagtcttcctttacg
HCR_Dan_her4_B1_S5-1	gaggagggcagcaaacggaaGGGCTGGAGTGTGTTGTTTGGCGCT
HCR_Dan_her4_B1_S5-2	TCTACCAGGGTCTCCAGATGTGACTtagaagagtcttcctttacg
HCR_Dan_her4_B1_S6-1	gaggagggcagcaaacggaaCCTCAATGGTACGGCGGGTGCTCTG
HCR_Dan_her4_B1_S6-2	CTCAGACTGGCATCGTGACTCGTGTtagaagagtcttcctttacg
HCR_Dan_her4_B1_S7-1	gaggagggcagcaaacggaaTACTTTCTGAACTTCAGTCCATGCC
HCR_Dan_her4_B1_S7-2	GAAGCTGCAGAGGCGGAAATCAATTtagaagagtcttcctttacg
HCR_Dan_her4_B1_S8-1	gaggagggcagcaaacggaaCTCTGAAAGCAGATTAAATGTCCTG
HCR_Dan_her4_B1_S8-2	CTCACACAGTTTCACTAAACTCTCTtagaagagtcttcctttacg
HCR_Dan_her4_B1_S9-1	gaggagggcagcaaacggaaATGATTGTGTGCATTGAGATCAAAC
HCR_Dan_her4_B1_S9-2	CACTGTAATAATCATTCTACACAGCtagaagagtcttcctttacg
HCR_Dan_her4_B1_S10-1	gaggagggcagcaaacggaaCAAACCCAATATGGGTGAAATGTTC
HCR_Dan_her4_B1_S10-2	GTGGTCATCGTATAGATGAAGAGAAtagaagagtcttcctttacg

Development • Supplementary information

Description	Sequence
**Oligonucleotides for *her9* HCR probe**	
HCR_Dan_her9_B4_S1-1	cctcaacctacctccaacaaATCTCCCACTGCCACACGCCAGCGG
HCR_Dan_her9_B4_S1-2	GGCGCAGCGCTTCAGAGGGCAAGTCattctcaccatattcgcttc
HCR_Dan_her9_B4_S2-1	cctcaacctacctccaacaaATCAGCTTTATGACTTATAGTCGGG
HCR_Dan_her9_B4_S2-2	TCACGAGAGAGAGTGTGAGTCCCTCattctcaccatattcgcttc
HCR_Dan_her9_B4_S3-1	cctcaacctacctccaacaaCCGGTAGAAGCCAAAAAGTGAATAT
HCR_Dan_her9_B4_S3-2	TGGCATGATCAAACACCAAAGTTGAattctcaccatattcgcttc
HCR_Dan_her9_B4_S4-1	cctcaacctacctccaacaaCCTGTTGAGCGGGGGCAGGCTGAGG
HCR_Dan_her9_B4_S4-2	GCACGTGAAGAGGCTGAGCCAAATGattctcaccatattcgcttc
HCR_Dan_her9_B4_S5-1	cctcaacctacctccaacaaACTGGCGTTGACAGTTACTGGAACG
HCR_Dan_her9_B4_S5-2	CGTGGGAGCCGAGCTGGCCTGGACGattctcaccatattcgcttc
HCR_Dan_her9_B4_S6-1	cctcaacctacctccaacaaGAGGTCATTCCCTGGACAGGAGATG
HCR_Dan_her9_B4_S6-2	CTGACGGCTTGTGGAACGCCCGAGAattctcaccatattcgcttc
HCR_Dan_her9_B4_S7-1	cctcaacctacctccaacaaATTGCTTTCTGCTCCGGCACTGACA
HCR_Dan_her9_B4_S7-2	AGTCTACCAGGGTCTCCACACCGGCattctcaccatattcgcttc
HCR_Dan_her9_B4_S8-1	cctcaacctacctccaacaaCGGAACTGAACGTATATTCTTCACC
HCR_Dan_her9_B4_S8-2	ACAAAAGGTCAACAAAGTCCCTTAGattctcaccatattcgcttc
HCR_Dan_her9_B4_S9-1	cctcaacctacctccaacaaACAACATCCAAATATTGGAATCAGC
HCR_Dan_her9_B4_S9-2	GTTGTATTCACAACAGCACATTCATattctcaccatattcgcttc
HCR_Dan_her9_B4_S10-1	cctcaacctacctccaacaaGCTTTGGCATGATACAGTCTTTTAC
HCR_Dan_her9_B4_S10-2	AACACTCATCATGAAAAGAACAAGGattctcaccatattcgcttc

Development • Supplementary information

Description	Sequence
**Oligonucleotides for *her15* HCR probe**	
HCR_Dan_her15_B2_S1-1	cctcgtaaatcctcatcaaaCTCTGAGGCCGCTGGATGAGGCGTC
HCR_Dan_her15_B2_S1-2	GTCCCAGTCAGGATTAGCTTTCCCAaaatcatccagtaaaccgcc
HCR_Dan_her15_B2_S2-1	cctcgtaaatcctcatcaaaAGGAGCTCATTAGCATTACAAAGCC
HCR_Dan_her15_B2_S2-2	GAATGAGGGTCTCGCTGCTCTATTTaaatcatccagtaaaccgcc
HCR_Dan_her15_B2_S3-1	cctcgtaaatcctcatcaaaCTCTGAGCAGAGCGAGGGGTTTGTG
HCR_Dan_her15_B2_S3-2	ATTGCTTCTTCAGGAGAGATGCTGTaaatcatccagtaaaccgcc
HCR_Dan_her15_B2_S4-1	cctcgtaaatcctcatcaaaAAGCTTGGAGTATTCAGTCATATAC
HCR_Dan_her15_B2_S4-2	TCGCAATTTGTGCTTCTCCTTGTTGaaatcatccagtaaaccgcc
HCR_Dan_her15_B2_S5-1	cctcgtaaatcctcatcaaaTGTGGAGTCTTGGGCCGCAGCTGCT
HCR_Dan_her15_B2_S5-2	TGCGAGTAGCCCTCGATCTGAGCGTaaatcatccagtaaaccgcc
HCR_Dan_her15_B2_S6-1	cctcgtaaatcctcatcaaaCAGCCTCGGAGCCCACAGACAGAAA
HCR_Dan_her15_B2_S6-2	GGGCTTCTTGCTGGAGACGTTGAGCaaatcatccagtaaaccgcc
HCR_Dan_her15_B2_S7-1	cctcgtaaatcctcatcaaaGTGTGCTGCTGATGGGGAGCTTCAG
HCR_Dan_her15_B2_S7-2	TGCGCCCGCGGCTCCTGCTTGATGTaaatcatccagtaaaccgcc
HCR_Dan_her15_B2_S8-1	cctcgtaaatcctcatcaaaCATCTAGTGTTCGAGCGTCTCTACC
HCR_Dan_her15_B2_S8-2	TCTCCTACATAAGAGACGGTAAACCaaatcatccagtaaaccgcc
HCR_Dan_her15_B2_S9-1	cctcgtaaatcctcatcaaaATCAGTGATGTACTATAGTAAATGG
HCR_Dan_her15_B2_S9-2	AATCTCTCATTTCTTCACACGATGTaaatcatccagtaaaccgcc
HCR_Dan_her15_B2_S10-1	cctcgtaaatcctcatcaaaCTAAACGTCAGCAGAAGACGAGGCG
HCR_Dan_her15_B2_S10-2	ACACTAATAAACCTCTGATCATAACaaatcatccagtaaaccgcc

Development • Supplementary information

Table S4. PCR conditions

PCR programs for genotyping *Df(her4;12)*, *Df(her2;12)*, *her6* and *her9* mutant zebrafish and zebrafish larvae. *her6* and *her9* qPCR programs and NICD genotyping PCR program. The primer sequences are given in Supplementary Table 1.

Df(her4;12)

			primer
step	Temp in °C	duration	p42
1	95	3min	p43
2	95	30s	p51
3	59	40s	
4	72	50s	
5	35x to 2		
6	72	5min	
7	4	end	

***her6* qPCR**

			primer
step	Temp in °C	duration	p372
1	95	30s	p373
2	95	15s	
3	60	1 min	
4	40x to 2		
5	95	1min	
6	40	1min	
7	from 65 °C to 95 °C with 0.07 °C per second		

Df(her2;15)

			primer
step	Temp in °C	duration	p213 1/4 of normal amount
1	95	3min	p220
2	95	30s	p222
3	60	40s	
4	72	60s	
5	35x to 2		
6	72	5min	
7	4	end	

***her9* qPCR**

			primer
			p376
step	Temp in °C	duration	
1	95	30s	p377
2	95	15s	
3	60	1 min	
4	40x to 2		
5	95	1min	
6	40	1min	
7	from 65 °C to 95 °C with 0.07 °C per second		

Development • Supplementary information

her6 knockout				
				primer
step	Temp in °C		duration	p5
1		95	3min	p10
2		95	30s	
3		56	40s	
4		72	50s	
5	35x to 2			
6		72	5min	
7		4	end	

her9 knockout				
				primer
step	Temp in °C		duration	p197
1		95	3min	p198
2		95	30s	
3		59	40s	
4		72	50s	
5	35x to 2			
6		72	5min	
7		4	end	

NICD genotyping				
Step	Temp in °C		duration	primer
1		95	3min	NICD F
2		95	1min	NICD R
3		80	1min	
4		95	3min	
5		95	1min	
6		62	1min	
7		72	75s	
8	3x to 2			
9		95	1min	
10		62	45s	
11		72	45s	
12	35x to 9			
13		72	5min	
14		8	end	

Table S5. Numbers of embryos documented or analyzed in each experiment

For each figure panel, the numbers of embryos documented or analyzed in each experiment are provided.

Fig.1
three larvae were imaged per condition and one representative image was selected

Fig. 2	
	n numbers
A, A'	3
B, B'	4
C, C'	2
D, D'	2

Fig. 3	
	n numbers
C, C'	3
D, D'	3
E, E'	2
F, F'	4
G, G'	2
H, H'	3
I, I'	3
J, J'	2
K, K'	2
L, L'	4 out of 5
M, M'	2
N, N'	4 out of 6

Fig. 4		
	n numbers	
A-A''	5	(also in Supplementary Movie M1)
B-B''	4	(also in Supplementary Movie M1)
C-C''	3	
D-D''	3	

Fig. 5	
	n numbers
A, A'	3
E, E'	3
B, B'	3
F, F'	3
C, C'	3
G, G'	3
D, D'	3
H, H'	3

Fig. 6	
	n numbers
C	5
C'	4
D	5
D'	2
E, F	2
E', F'	3

Development • Supplementary information

Fig. 7		
	n numbers	
A-A''	4	(also in Supplementary Movie M2)
B-B''	5	(also in Supplementary Movie M2)
C-C''	3	
D-D''	3	

Fig. 8
Three larvae were imaged per condition and one representative image was selected.

Fig. 9	
Three larvae were imaged per condition and one representative image was selected, except for:	
B'	9 out of 14 larvae showed the displayed phenotype
E'	9 out of 15 larvae showed the displayed phenotype
J'	11 out of 15 larvae showed the displayed phenotype

Fig. 10		
	n numbers	
A, B	1	
A', B'	3	
C-C''	4	(also in Supplementary Movie M2)
D-D''	4	(also in Supplementary Movie M2)
E-E''	3*	*in Fig. 10E and Supplementary Movie M4, a *her2, her15* heterozygous larva was used (1 out of 3 larvae). Two more larvae were WT for *her2, her15*.
F-F''	4*	*in Fig. 10F and Supplementary Movie M4, a *her6* heterozygous larva was used (1 out of 4 larvae). 3 more larvae were *her6* WT

Development • Supplementary information

Table S6. Data Analysis Table of *her6* and *her9* qPCR (*her6* and *her9* expression in *her6* and *her9* mutants)

Table 6A shows the qPCR Primary Data. *her6* and *her9* expression were measured in wild type and mutant embryos, respectively, each in three biological replicates, which were each analyzed in three technical replicates (numbers are means of technical replicates, data for technical replicates are not shown). *actb2* was used as reference gene to normalize expression. The mean quantitative cycle (mean Cq) of the technical replicates for each sample/target combination was calculated in column C. The average Cq value for the wild type embryos (control group) is shown in column D. The relative difference (ΔCq) between the average Cq for the control group (column D) and the mean Cq (column C) per individual sample within each target is given in column E. The relative quantities were calculated from the ΔCq (i.e., 2ΔCq) and the reaction efficiency from the standard curve (column F). For each wild type/mutant combination, a normalization factor (column G) is calculated from the geometric mean of the associated reference gene relative quantities. The relative quantity (column F) is divided by the normalization factor (column G) to obtain the relative normalized expression per sample (column H). log transformation of column H results in column I.

Column J and K are the result of calculating the geometric mean for the samples within each genotype. Standard deviation (SD), standard error of the mean (SEM) and confidence intervals are calculated from the log transformed normalized expression (columns L–O). The small table below the main table shows the PCR efficiencies that were determined and used for calculating the relative quantities. **Supplementary Table 6B** shows the normalized expression and error bars in numbers. The bars represent data from the genotype expression (column J) and the error bars are calculated by 2^(column K +/- column M). **Supplementary Table 6C** shows the tests for statistical significance (3 samples for each genotype / 2 degrees of freedom). Significant differences with p< 0.05 are marked in orange. The analysis was performed based on Taylor, S. C., Nadeau, K., Abbasi, M., Lachance, C., Nguyen, M., & Fenrich, J. (2019). The ultimate qPCR experiment: producing publication quality, reproducible data the first time. *Trends in Biotechnology*, *37*(7), 761-774.

Table 6: qPCR Primary Data

A	B	C	D	E	F	G	H	I	J	K	L	M	N	O
Gene	Genotype _sample	Mean Cq of technical triplicates	Control group Avg Cq per gene	ΔCq per sample per gene	RQ (rel. quantity) (1+E)^ΔCq	GEOMEAN (norm. factor) [=RQ(actb2)]	Normalized expression per sample	Log2 norm. Expression	Genotype expression	Log2 (J)	Standard deviation SD (I)	SEM	Confidence interval lower limit	Confidence interval upper limit
her6	WT_1	24.500		-0.177	0.884	0.857	1.032	0.045						
her6	WT_2	24.640	24.323	-0.317	0.801	0.903	0.888	-0.172	1.004	0.005	0.154	0.089	0.947	1.057
her6	WT_3	23.830		0.493	1.412	1.293	1.092	0.127						
her6	dKO_1	21.980		2.343	5.145	0.958	5.367	2.424						
her6	dKO_2	22.370		1.953	3.917				4.640	2.214	0.323	0.186	1.484	2.324
her6	dKO_3	22.090		2.233	4.764	1.218	3.912	1.968						
her6	her6KO_1	23.160		1.163	2.255	1.025	2.199	1.137						
her6	her6KO_2	23.890		0.433	1.354	0.958	1.412	0.498	1.734	0.794	0.331	0.191	0.992	1.587
her6	her6KO_3	23.870		0.453	1.373	0.863	1.590	0.669						
her6	her9KO_1	23.410		0.913	1.893	1.173	1.614	0.691						
her6	her9KO_2	23.620		0.703	1.635	1.130	1.447	0.533	1.327	0.408	0.430	0.248	0.790	1.696
her6	her9KO_3	24.150		0.173	1.129	1.227	0.920	-0.120						
her9	WT_1	24.300		-0.503	0.701	0.857	0.818	-0.290						
her9	WT_2	24.350	23.797	-0.553	0.676	0.903	0.749	-0.417	1.066	0.093	0.615	0.355	0.502	2.177
her9	WT_3	22.740		1.057	2.110	1.293	1.632	0.707						
her9	dKO_1	21.210		2.587	6.223	0.958	6.493	2.699						
her9	dKO_2	21.790		2.007	4.130				5.730	2.519	0.273	0.158	1.559	2.164
her9	dKO_3	21.250		2.547	6.050	1.218	4.968	2.313						
her9	her6KO_1	23.290		0.507	1.431	1.025	1.395	0.481						
her9	her6KO_2	23.920		-0.123	0.917	0.958	0.956	-0.065	1.123	0.168	0.292	0.169	0.867	1.258
her9	her6KO_3	23.980		-0.183	0.878	0.863	1.018	0.025						
her9	her9KO_1	21.790		2.007	4.130	1.173	3.521	1.816						
her9	her9KO_2	22.070		1.727	3.389	1.130	2.999	1.584	2.834	1.503	0.428	0.247	1.169	2.495
her9	her9KO_3	22.540		1.257	2.431	1.227	1.981	0.986						
actb2	WT_1	16.740		-0.207	0.857									
actb2	WT_2	16.670	16.533	-0.137	0.903									
actb2	WT_3	16.190		0.343	1.293									
actb2	dKO_1	16.590		-0.057	0.958									
actb2	dKO_2	excluded (19.000)												
actb2	dKO_3	16.270		0.263	1.218									
actb2	her6KO_1	16.500		0.033	1.025									
actb2	her6KO_2	16.590		-0.057	0.958									
actb2	her6KO_3	16.730		-0.197	0.863									
actb2	her9KO_1	16.320		0.213	1.173									
actb2	her9KO_2	16.370		0.163	1.130									
actb2	her9KO_3	16.260		0.273	1.227									

Development • Supplementary information

PCR efficiency: (E)	
her6	1.011702888
her9	1.027519485
actb2	1.113346713

Table 6C: Statistical Significance of Differences between Genotypes

her6 relative expression values

	p-values for comparisons			
Genotypes	WT	her6 sKO	her9 sKO	dKO
WT				
her6 sKO	0.041			
her9 sKO	0.212	0.268		
dKO	0.007	0.019	0.012	

her9 relative expression values

	p-values for comparisons			
Genotypes	WT	her6 sKO	her9 sKO	dKO
WT				
her6 sKO	0.866			
her9 sKO	0.030	0.022		
dKO	0.006	0.004	0.038	

$p < 0.05$

Development • Supplementary information

Table S7. Quantification of pH3 and Sox2 immunoreactive cell nuclei in the TPZ

Confocal stacks of embryos	Planes analyzed	Em-bryo	*Notch-independent*	Nuclei counts				pH3 count ratio	pH3 count ratio
			WT control (N-indep)	$Sox2^{high}$	$Sox2^{low}$	pH3 $Sox2^{high}$	pH3 $Sox2^{low}$	pH3 $Sox2^{high}$ / $Sox2^{high}$	pH3 $Sox2^{low}$ / $Sox2^{low}$
2020_05_24_sample1_Hes1_pH3_488 Sox2_555_3dpf_her6WT her9WT_B8_view2_AP	z14-z23	em1	WT -right	156	182	3	1	1.92%	0.55%
2020_05_24_sample1_Hes1_pH3_488 Sox2_555_3dpf_her6WT her9WT_B8_view2_AP	z14-z23	em1	WT -left	154	188	3	0	1.95%	0.00%
2020_05_21_sample1_Hes1_pH3_488 Sox2_555_3dpf_her6WT_her9WT_B7__AP	z24-33	em2	WT -right	160	156	5	1	3.13%	0.64%
2020_05_21_sample1_Hes1_pH3_488 Sox2_555_3dpf_her6WT_her9WT_B7__AP	z24-33	em2	WT -left	150	192	3	0	2.00%	0.00%
			average	155.00	179.50	3.50	0.50	2.26%	0.28%
			standard deviation	4.16	16.20	1.00	0.58	0.58%	0.35%
								pH3 count ratio	pH3 count ratio
			her6, her9 DKO	$Sox2^{high}$	$Sox2^{low}$	pH3 $Sox2^{high}$	pH3 $Sox2^{low}$	pH3 $Sox2^{high}$ / $Sox2^{high}$	pH3 $Sox2^{low}$ / $Sox2^{low}$
2020_05_20_sample1_Hes1_pH3_488 Sox2_555_3dpf_her6KO_her9KO_B1__AP	z16-z25	em1	DKO -right	123	25	3	0	2.44%	0.00%
2020_05_20_sample1_Hes1_pH3_488 Sox2_555_3dpf_her6KO_her9KO_B1__AP	z16-z25	em1	DKO -left	114	28	1	0	0.88%	0.00%
2020_05_21_sple1_CS2023_pH3_488 Sox2_555_3dpf_her6KO_her9KO_B3__AP	z63-72	em2	DKO -right	112	29	1	1	0.89%	3.45%
2020_05_21_sple1_CS2023_pH3_488 Sox2_555_3dpf_her6KO_her9KO_B3__AP	z63-72	em2	DKO -left	96	33	1	0	1.04%	0.00%
2020_05_20_sple1_CS2023_pH3_488 Sox2_555_3dpf_her6KO_her9KO_B2_dorsal_AP__	z13-z22	em3	DKO -right	131	53	3	1	2.29%	1.89%
2020_05_20_sple1_CS2023_pH3_488 Sox2_555_3dpf_her6KO_her9KO_B2_dorsal_AP__	z13-z22	em3	DKO -left	127	59	2	0	1.57%	0.00%
			average	117.17	37.83	1.83	0.33	1.56%	0.88%
			standard deviation	12.70	14.43	0.98	0.52	0.70%	1.46%

Development • Supplementary information

Confocal stacks of embryos	Planes analyzed	Em-bryo	Notch dependent	Sox2^{high}	Sox2^{low}	pH3 Sox2^{high}	pH3 Sox2^{low}	pH3 count ratio	pH3 count ratio
			WT control (N-dep)	Sox2^{high}	Sox2^{low}	pH3 Sox2^{high}	pH3 Sox2^{low}	pH3 Sox2^{high} / Sox2^{high}	pH3 Sox2^{low} / Sox2^{low}
2020_05_23_sple5_CS2023_pH3_488 Sox2_555_3dpf_her4WT_her15WT_G9__AP	z13-22	em1	WT -right	171	156	2	1	1.17%	0.64%
2020_05_23_sple5_CS2023_pH3_488 Sox2_555_3dpf_her4WT_her15WT_G9__AP	z15-24	em1	WT -left	177	170	3	2	1.69%	1.18%
2020_05_21_sple5_CS2023_pH3_488 Sox2_555_3dpf_her4WT_her15WT_F12__AP___	z14-23	em2	WT -right	174	192	6	1	3.45%	0.52%
2020_05_21_sple5_CS2023_pH3_488 Sox2_555_3dpf_her4WT_her15WT_F12__AP___	z14-23	em2	WT -left	206	211	1	1	0.49%	0.47%
2020_05_21_sple4_CS2023_pH3_488 Sox2_555_3dpf_her4WT_her15WT_E2__AP	28-37	em3	WT -right	149	265	4	1	2.68%	0.38%
2020_05_21_sple4_CS2023_pH3_488 Sox2_555_3dpf_her4WT_her15WT_E2__AP	28-37	em3	WT -left	139	217	4	2	2.88%	0.92%
			average	169.33	201.83	3.33	1.33	1.97%	0.66%
			standard deviation	23.50	38.77	1.75	0.52	1.13%	0.31%
								pH3 count ratio	pH3 count ratio
			Df(her4;12), Df(her2;15)	Sox2^{high}	Sox2^{low}	pH3 Sox2^{high}	pH3 Sox2^{low}	pH3 Sox2^{high} / Sox2^{high}	pH3 Sox2^{low} / Sox2^{low}
2020_05_21_sple4_CS2023_pH3_488 Sox2_555_3dpf_her4KO_her15KO_E3__AP	z8-z17	em1	DKO -right	205	187	1	2	0.49%	1.07%
2020_05_21_sple4_CS2023_pH3_488 Sox2_555_3dpf_her4KO_her15KO_E3__AP	z12-21	em1	DKO -left	211	223	1	3	0.47%	1.35%
2020_05_21_sple5_CS2023_pH3_488 Sox2_555_3dpf_her4KO_her15KO_H2__AP	z17-26	em2	DKO -right	131	148	2	1	1.53 %	0.68%
2020_05_21_sple5_CS2023_pH3_488 Sox2_555_3dpf_her4KO_her15KO_H2__AP	z17-26	em2	DKO -left	144	169	1	1	0.69%	0.59%
2020_05_21_sple4_CS2023_pH3_488 Sox2_555_3dpf_her4KO_her15KO_D5__AP	z9-z18	em3	DKO -right	205	182	4	1	1.95%	0.55%
2020_05_21_sple4_CS2023_pH3_488 Sox2_555_3dpf_her4KO_her15KO_D5__AP	z9-z18	em3	DKO -left	199	182	5	1	2.51%	0.55%
			average	182.50	181.83	2.33	1.50	1.28%	0.82%
			standard deviation	35.30	24.62	1.75	0.34	0.85%	0.33%

Development • Supplementary information

Table S8. Summary Comparisons Quantification of pH3 and Sox2 immunoreactive cell nuclei

**N-independent**			**p value**
	$Sox2^{high}$ WT	$Sox2^{high}$ *her6,her9* DKO	
average	155.00	117.17	0.000370
SD	4.16	12.70	
	$Sox2^{low}$ WT	$Sox2^{low}$ *her6,her9* DKO	
average	179.50	37.83	0.000008
SD	16.20	14.43	
	pH3 $Sox2^{high}$ / $Sox2^{high}$ WT	pH3 $Sox2^{high}$ / $Sox2^{high}$ *her6,her9* DKO	
average	2.26%	1.56%	0.115781
SD	0.58%	0.70%	
	pH3 $Sox2^{low}$ / $Sox2^{low}$ WT	pH3 $Sox2^{low}$ / $Sox2^{low}$ *her6,her9* DKO	
average	0.28%	0.88%	0.379410
SD	0.35%	1.46%	
**N-dependent**			**p value**
	$Sox2^{high}$ WT	$Sox2^{high}$ *Df(her4;12), Df(her2;15)*	
average	169.33	182.50	0.467067
SD	23.50	35.30	
	$Sox2^{low}$ WT	$Sox2^{low}$ *Df(her4;12), Df(her2;15)*	
average	201.83	181.83	0.315558
SD	38.77	24.62	
	pH3 $Sox2^{high}$ / $Sox2^{high}$ WT	pH3 $Sox2^{high}$ / $Sox2^{high}$ *Df(her4;12), Df(her2;15)*	
average	1.97%	1.28%	0.206500
SD	1.13%	0.85%	
	pH3 $Sox2^{low}$ / $Sox2^{low}$ WT	pH3 $Sox2^{low}$ / $Sox2^{low}$ *Df(her4;12), Df(her2;15)*	
average	0.66%	0.82%	0.558467
SD	0.31%	0.33%	

**pH3 count ratio $Sox2^{high}$ vs $Sox2^{low}$**			**p value**
	pH3 $Sox2^{high}$ / $Sox2^{high}$ WT (N-indep)	pH3 $Sox2^{low}$ / $Sox2^{low}$ WT (N-indep)	
average	2.26%	0.28%	0.002446
SD	0.58%	0.35%	
	pH3 $Sox2^{high}$ / $Sox2^{high}$ WT (N-dep)	pH3 $Sox2^{low}$ / $Sox2^{low}$ WT (N-dep)	
average	1.97%	0.66%	0.029752
SD	1.13%	0.31%	
	pH3 $Sox2^{high}$ / $Sox2^{high}$ WT (all)	pH3 $Sox2^{low}$ / $Sox2^{low}$ WT (all)	
average	2.136%	0.53%	0.000249
SD	0.913%	0.363%	

Development • Supplementary information
